# Changes of upper ocean disturbance caused by tropical cyclones in the Western North Pacific main development region (1993–2021)

**DOI:** 10.1371/journal.pone.0320143

**Published:** 2025-04-24

**Authors:** Yujun Liu, Feiyan Chen, Zhifei Ma, Yating Shao, Junhui Yan

**Affiliations:** 1 School of Geographic Sciences, Xinyang Normal University, Xinyang, China; 2 Henan Key Technology Engineering Research Center of Microwave Remote Sensing and Resource Environment Monitoring, Xinyang, China; 3 Xinyang Key Laboratory of Climate Change & Environmental Evolution, Xinyang Normal University, Xinyang, China; 4 North-south transitional zone typical vegetation phenology observation and research station of Henan Province, Xinyang Normal University, Xinyang, China; 5 College of Geoscience and Surveying Engineering, China University of Mining & Technology, Beijing, China; 6 State Key Laboratory of Resources and Environmental Information System, Institute of Geographic Sciences and Natural Resources Research, Beijing, China; King Abdullah University of Science and Technology, SAUDI ARABIA

## Abstract

During the passage of tropical cyclones (TCs) cause severe disturbances in the upper ocean, especially the TC main development region (MDR) (5°-23°N, 127°-160°E) in the Western North Pacific. The main attributes affecting the upper ocean disturbance were selected and weighted by the CRITIC weight method. An upper ocean disturbance (UOD) index with TCs intensity factors (MSW, V_h_), ocean dynamic factors (SSC, RV, EPV) and ocean thermal factors (ΔSST, ΔMLD) was established. The change of UOD index in different time scales was analyzed by calculating each TC accumulation, monthly accumulation, and Interannual accumulation. The UOD index was closely related to the ENSO (El Niño–Southern Oscillation) climate anomaly, and their interannual correlation was as high as 0.83. Generally, the UOD index was higher in El Niño years and lower in La Niña years. In the past 10 years, the ocean thermal factors of UOD index have been increasing, especially in La Niña years. This is mainly due to the increasing SST, decreasing MLD and seawater salinity in recent years, which have changed upper ocean stratification. These results offer insights for the comprehensive analysis of the response and variation trend of the upper ocean to TC in the MDR of the Western North Pacific in recent years.

## Introduction

Tropical cyclones (TCs) are widely active in the tropical and subtropical oceans. They are mainly driven by the heat transfer in the ocean. As a strong wind weather system, TC has a strong mechanical energy input to the ocean when it passes the sea surface, which will cause various response processes in the upper ocean and affect the transport of water, energy, and momentum at the sea-air interface [1–3]. As a catastrophic weather system, TCs brings great threat to human life, property, and surrounding environment. Additionally, TCs can transport heat from low latitudes to high latitudes regions, promoting global heat circulation and balance, and the abundant precipitation they bring can also play a positive role in alleviating drought [4]. TCs have important influence on the upper ocean and have significant research value.

**Fig 1:**
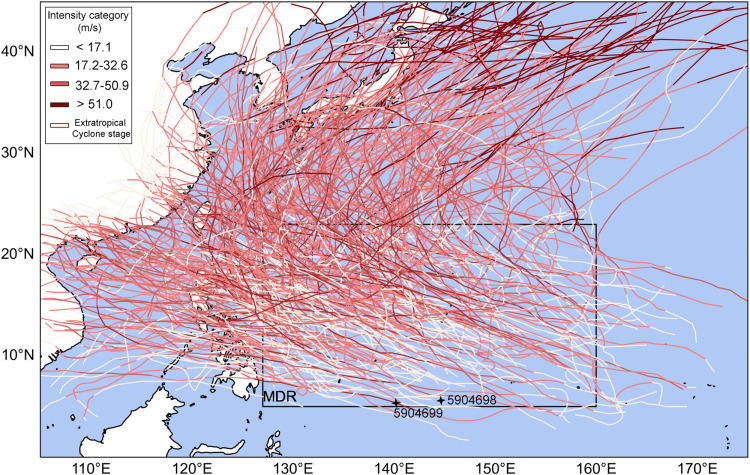
Schematic diagram of TCs main development region (MDR) in the Western North Pacific. The lines represent TC tracks, with color depth indicating TC intensity according to the China Meteorological Administration (CMA) classification standards. Two in situ Argo floats (black asterisks) platform numbers were 5904699 and 5904698, and their corresponding dates were July 16, 2021 and July 21, 2016, respectively.

Global warming caused by human activities and natural low-frequency oscillations may have an impact on TCs. There are interannual, intergenerational and even longer time scale variations in the frequency, intensity, and duration of TCs [5,6]. Under the influence of global warming, sea surface temperature (SST) is expected to increase significantly [7–10]. While the intensity of TCs tends to strengthen, their frequency is likely to decrease [11,12]. The relationship between SST and the intensification of TCs varies greatly among different basins, with changes in the SST gradient having a more significant impact on the formation of TCs [13,14]. Globally, 30.7% of TC occur in the Western North Pacific, the region with the highest frequency of tropical cyclones, where tropical cyclones disturbances are known as typhoons [15]. El Niño-Southern Oscillation (ENSO) is an important factor affecting the interannual variation of TCs activity in the Western North Pacific. During the warm (cold) phase of the ENSO, global warming amplifies cyclonic (anticyclonic) circulation, leading to an increase (decrease) in the frequency of tropical cyclone genesis in the Western North Pacific [16]. At the same time, westerly (easterly) wind anomalies on the southern flank of the anomalous cyclone (anticyclone) generate eastward (westward) current anomalies in the mixed layer, inducing anomalous warm (cold) zonal advection. In addition, wind anomalies can trigger oceanic downwelling (upwelling) Kelvin waves, deepening (shallowing) the thermocline in the eastern equatorial Pacific, resulting in anomalous warm (cold) vertical advection [17]. The average TCs genesis region usually southeastward displacement during El Niño years [18]. In El Niño years, TCs are more frequent, they live longer, and tend to be more intense before encountering continents or moving to cooler mid-latitude waters, while La Niña years have the opposite effect [15,19–21]. There is a main development region (MDR) of TCs in the Western North Pacific (5°-23°N, 127°-160°E), where more than 80% of TCs are generated (Fig 1) [22]. In recent years, the frequency of Central Pacific (CP) El Niño events has increased, and the active TC region has shifted west and northwest [23], bringing it closer to the MDR during CP El Niño events.

In the previous studies, researchers mostly focused on analyzing the impact of a certain TC transit on the sea area and some obvious parameters [24–26]. For example, the maximum sea surface temperature (SST) cooling most often occurs 1 day after TC passage, but it can also vary from 1 day before passage to 7 days after passage [27]. The maximum SST decrease caused by typhoon Kaemi in 2006 was 2°C, which was mainly related to upwelling and rainfall near the typhoon track [28]. The passage of typhoon Chan-hom (2015) increased the mixed layer depth (MLD) from 32.75 m to 81.72 m in the study area [25]. Strong Ekman pumping during the passage of typhoon Matsa (2005) facilitated the growth of phytoplankton chlorophyll-a, increasing primary productivity there [29]. Stronger, slower-moving TCs have a greater impact on the upper ocean [30]. Cold eddies tend to enhance the impact of TCs on the upper ocean, while warm eddies tend to suppress this oceanic response [31]. It is obvious that there are limitations to the analysis of some obvious parameter features and individual TC cases. TCs affect the ocean involves various aspects [32–34], and the analysis of individual TC cases and some obvious characteristics are not conducive to the comprehensively reflect the upper ocean disturbance (UOD) by TCs. At present, there is a lack of an upper ocean disturbance index system that can integrate various influencing factors to indicate TC cases and reflect long-term climate change.

Thus, this study selected the MDR region where TCs frequently generate in the Western North Pacific and integrated the various attributes of TCs affecting the upper ocean in an attempt to establish a comprehensive evaluation index of the upper ocean disturbance by TCs that can reflect individual TC cases and the climate change trend from 1993 to 2021. In Section 2, data and methods are introduced. In Section 3, statistical characteristics of UOD index under different time scales, including each TC accumulation, monthly and interannual accumulations are analyzed. The reasons why the ocean thermal factors in UOD index are higher in La Niña years and their changing trends in the most recent ten years are discussed in Section 4.

## Data and methods

### Data

This study used observation/reanalysis products: 1) daily Optimum Interpolation (OI) SST from Remote sensing system [35,36], combined with microwave and infrared data, with a resolution of 0.25 (https://www.remss.com/measurements/sea-surface-temperature/)°; 2) sea surface wind (SSW) from CCMP (Cross-Calibrated Multi-Platform) wind vector analysis product 2.0 version with a spatial resolution of 0.25° (https://www.remss.com/measurements/ccmp/) [37]; 3) sea surface current (SSC), sea water salinity and MLD data obtained from GLORYS12V1 product provided by the Copernicus Marine Environment Monitoring Service (CMEMS) with a spatial resolution of 0.083˚ (https://doi.org/10.48670/moi-00021); 4) the Niño3.4 index from NOAA Physical Sciences Laboratory (https://psl.noaa.gov/data/correlation/nina34.anom.data), which is used to describe the El Niño (warm) and La Niña (cool) events based on 5 consecutive overlapping 3-month periods at or above the threshold of ± 0.5°C; 5) best track data for the TC obtained from China Meteorological Administration (CMA) (tcdata.typhoon.org.cn) [38,39]; 6) the in situ data came from the Argo program, the Argo platform numbers were 5904698 and 5904699 [40]. The two floats were relatively close to each other on July 21, 2016, and July 16, 2021, which could provide effective temperature and salinity data (http://www.argo.ucsd.edu/).

All products used here were from 1993 to 2021. selected TC whose activity time is longer than 1d in the MDR was selected. All data were processed by daily average, and a total of 2089 ordered samples were collected (S1 Table).

### Methods

In the selection of UOD index attributes, we selected and calculated physical ocean parameters that can well reflect the UOD caused by TC passage: Ekman pumping velocity (EPV) [41,42], Relative Vorticity (RV) [43], ΔSST (the absolute value of the SST difference between the two days before and after TC passage) [44,45], ΔMLD (the MLD difference between the two days before and after TC passage), SSC, Translation Speed (V_h_), and Maximum Surface Wind (MSW). Each attribute of the samples was calculated with the mean value of ocean parameters in the 2°× 2° region of TC’s location center. Before calculating the weights, the sample data were normalized by Min-Max feature scaling.

In this study, the CRITIC weight method was used to calculate the indicator weight [46], which could better reflect the degree of dispersion within the attributes and the correlation between the attributes. There are *m* TC samples and *n* evaluation indexes, which can form a symmetric matrix X=m×n ($x_{ij}$). Let the elements in the matrix after normalization processing be $x_{ij}^{\text{'}}$.


$$\sigma_{j}=\sqrt{\frac{\sum_{i=1}^{m}\left(x_{ij}^{\text{'}}-x_{j}^{\text{'}}\right)}{m-1}}$$ (1)

$$f_{j}=\sum_{i=1}^{m}\left(1-r_{ij}\right)$$ (2)

$$C_{j}=\sigma_{j}f_{j}$$ (3)

Here, $\sigma_{j}$ reflects the contrast intensity between the attributes, $f_{j}$ represents a measure of the conflict between the attributes, and $C_{j}$ reflects the information carrying capacity of the attributes. Then the index weight $w_{j}$ can be expressed as:


$$w_{j}=\frac{C_{j}}{\sum_{j=1}^{n}C_{j}}$$ (4)

Then, the UOD index can be calculated as:


$$UODindex=\sum_{j=1}^{n}100\times w_{j}\times x_{ij}^{\text{'}}$$ (5)

Then the UOD index was accumulated by each TC, monthly and interannually to analyze the trend change of UOD index for different TCs and different time scales. The Mann-Kendall (MK) breakpoint test was used to detect the breakpoint of UOD index (Eq (6)-Eq (7)). This method does not require variables to have normal distribution characteristics, nor is it disturbed by a few outliers, and is often used in trend analysis in hydrology and meteorology [47,48].


$$UF_{k}=\frac{\left|s_{k}-E\left(s_{k}\right)\right|}{\sqrt{\text{var}\left(s_{k}\right)}},k=1,2\cdots ,n$$ (6)

$$UB_{k}=-UF_{k}\left(k=n,n-1,\cdots ,1\right)$$ (7)

Here, $s_{k}$ is the rank sequence constructed for the time sequence. $UF_{k}$ is the standard positive distribution, which is in the time sequence order $x_{1},x_{2},\cdots x_{n}$, and $UB_{k}$ is the reverse order of $UF_{k}$ time sequence. $E\left(s_{k}\right)$ and $\text{var}\left(s_{k}\right)$ are the mean and variance of $s_{k}$. If $UF_{k}$,$UB_{k}$, the two curves intersect at the confidence lines, the intersection is the beginning of the mutation.

In addition, Continuous wavelet transform (CWT), Cross wavelet transform (XWT) and wavelet coherence (WTC) were also used to analyze oscillations in the UOD index and verify the relationship between UOD index and Niño3.4 index [49–52]. And Brunt-Vaisala frequencies were calculated to analyze ocean stratification changes by Argo floats, which represent the stability of sea water to vertical displacements such as those caused by convection [53].

## Results

### Each TC accumulative disturbance in MDR

The weight of each attribute obtained according to Eq (4) can be shown in Table 1. Among these index attributes, ΔSST, MSW and SSC have the largest weights, accounting for 20.37%, 19.91% and 14% respectively, and the total is 54.28% of the whole. MSW, V_h_ and SSW mainly represent TC intensity factors; SSC, RV and EPV represent ocean dynamic factors; ΔSST and ΔMLD reflect ocean thermal factors. Overall, typhoon intensity factors are the most important influencing factors, followed by ocean thermal factors and dynamic factors.

**Table 1:** Weight of UOD index attributes (%).

	MSW (m/s)	V_h_ (m/s)	SSW (m/s)	SSC (m/s)	RV (s-1)	EPV (m/s)	ΔSST (°C)	ΔMLD (m)
Attributes (%)	19.91%	12.80%	8.31%	14.00%	9.47%	5.33%	20.37%	9.80%

Note: MSW: Maximum Surface Wind, V_h_: Translation Speed, SSW: Sea surface wind, SSC: Sea surface current, RV: Relative Vorticity, EPV: Ekman pumping velocity, ΔSST: the SST difference between the two days before and after TC passage, ΔMLD: the MLD difference between the two days before and after TC passage.

**Fig 2:**
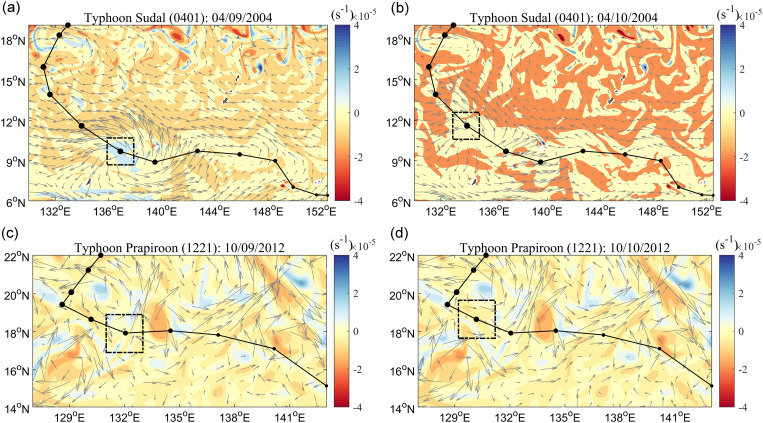
The ocean dynamic environment during the Typhoon Sudal and Prapiroon passing through ocean eddy. (A) and (B) show the ocean relative vorticity fields during and after the passage of typhoon Sudal, respectively. (C) and (D show the ocean relative vorticity fields during and after the passage of Typhoon Prapiroon, respectively. The black box is the study area, and the black dots and lines correspond to the intensity and tracks of TC. The arrows indicate ocean flow fields.

The average daily disturbance (S1 Table) and TC accumulative disturbance on the upper ocean were counted. Table 2 shows the top 10 TCs by UOD index from 1993–2021, where “duration” and “UOD index” are the accumulative results of each TC, and each attribute factor is a calculated daily average. TCs ranked in the top 10 by UOD index generally lasted more than 11 days in the MDR. Generally, TC was stronger, and the translation speed was slower, which caused greater disturbance of the ocean dynamic and thermal factors, and higher UOD index. The initial conditions of the upper ocean also affected the UOD index. The disturbance would be increased when TC passed through the cold eddy, while the opposite was true when TC passed through the warm eddy. Typhoon Sudal (TC ID:0401) and typhoon Prapiroon (TC ID:1221), which ranked the 2nd and 7th in UOD index, are examples of this effect (Fig 2). The intensity of typhoon Sudal was not great compared with other TCs, but due to how Sudal was affected by cold eddy (Fig 2A–2B), the RV and SSC obviously increased during Sudal’s passage, and the dynamic disturbance to the ocean was enhanced. Sudal’s slow translation speed caused it to stay in MDR for a long time, which was also the reason for the larger UOD index. The intensities of Typhoon Prapiroon and Sudal were similar, but the UOD index of Prapiroon only ranked 7th, mainly because Prapiroon was inhibited by the warm eddy, resulting in weaker RV and SSC and small dynamic disturbance to the ocean (Fig 2C–2D).

**Table 2:** Top 10 TCs in UOD index.

TC ID	Average								Accumulative	
MSW (m/s)	V_h_ (m/s)	SSW (m/s)	SSC (m/s)	RV (s^-1^)	EPV (m/s)	ΔSST (°C)	ΔMLD (m)	Duration (day)	UOD index
2102	32.89	5.47	20.19	0.45	3.69×10^6^	5.98×10^4^	2.07	−.86	11	0.910
0401	32.88	3.96	12.74	0.45	5.02×10^6^	2.74×10^4^	1.62	−1.51	13	0.858
9432	31.58	4.88	9.13	0.32	2.25×10^6^	0.77×10^4^	2.02	−3.10	15	0.778
0321	39.85	5.55	13.38	0.38	4.35×10^6^	2.73×10^4^	1.81	−7.46	10	0.682
1902	33.90	3.68	11.06	0.36	2.83×10^6^	1.36×10^4^	1.61	8.77	12	0.670
0922	38.27	2.67	8.03	0.28	1.91×10^6^	0.57×10^4^	2.41	−0.63	12	0.667
1221	31.90	2.76	8.34	0.24	1.02×10^6^	0.78×10^4^	2.48	−7.82	12	0.618
1511	46.92	4.30	11.08	0.30	1.34×10^6^	1.52×10^4^	2.89	−6.39	9	0.607
9722	39.61	5.17	13.39	0.41	3.83×10^6^	1.90×10^4^	1.75	−5.13	9	0.590
9725	39.45	4.72	10.49	0.40	1.57×10^6^	0.84×10^4^	1.95	5.59	10	0.589

### Relationship between monthly change of accumulative UOD index and ENSO

**Fig 3:**
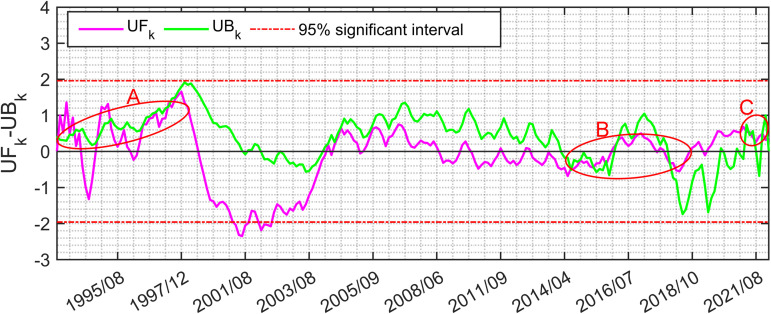
Accumulative monthly trend change of UOD index. The red circles represent three mutation periods: A:1993–1997, B:2014–2018, and C:2021. The area between the two red lines represents the 95% significance interval, while the black line represents the critical threshold.

MK breakpoint test was calculated by constructing positive sequence ($UF_{k}$) and reverse sequence ($UB_{k}$) [54]. The trend changes of monthly accumulative UOD index from 1993 to 2021 showed a fluctuating characteristic (Fig 3). In the three time periods of 1993–1999, 2004–2008, and 2015–2021, the overall $UF_{k}>0$, mainly showed a fluctuating upward trend, and the rest of the time, the overall $UF_{k}<0$ showed a fluctuating downward trend. Circles A, B, and C reflected three obvious mutation periods 1993–1997, 2014–2018, and 2021 in the past three decades. During these time periods, the $UF_{k}$ and $UB_{k}$ curves frequently intersected within the confidence lines, and UOD index fluctuated sharply, which might be affected by ENSO.

**Fig 4:**
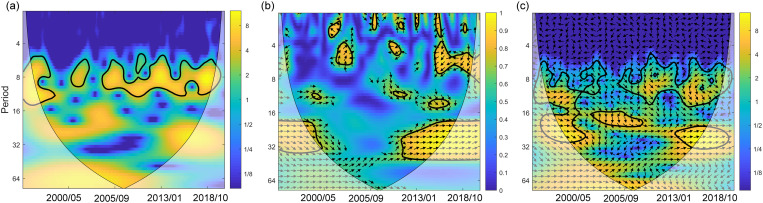
Relationship between UOD index and Nino3.4 index. (A) is CWT of UOD index, (B) and (C) show WTC and XWT between UOD index and Nino3.4 index. The white transparent area represents the region within the COI, and the black contour indicates 95% confidence level. The relative phase relationship is shown as black arrows, where the right pointing arrow denotes in-phase, while the left pointing arrow represents anti-phase. The arrow pointing straight down shows the UOD index leading Nino3.4 index changes by 90°.

ENSO is an important factor affecting the interannual variation of TCs activity in the Western North Pacific. The intensity and number of TCs affected by ENSO will change significantly, which will affect the disturbance of the upper ocean. Fig 4 shows the periodicity of the smoothed UOD index and its correlation with Nino3.4 index. UOD index fluctuated mainly in a period of 6–14 months (Fig 4A). The XWT spectrum can reflect the relationship between sequences in the high power region, while WTC reflects that in the low power region [51,52,55]. WTC and XWT spectra jointly revealed that UOD index and Nino3.4 index had significant resonance large periods (16–32 months) during the two upward trend periods of 1993–1999 and 2015–2021. The XWT spectrum also showed significant high-frequency power within small periods of 6–14 months (Fig 4B–4C). In addition, Fig 4B–4C also show that UOD index and Nino3.4 index had stable in-phase relationship in the large periods, and UOD index was ahead of Nino3.4 index in the small periods.

### Interannual change of accumulative UOD index

**Fig 5:**
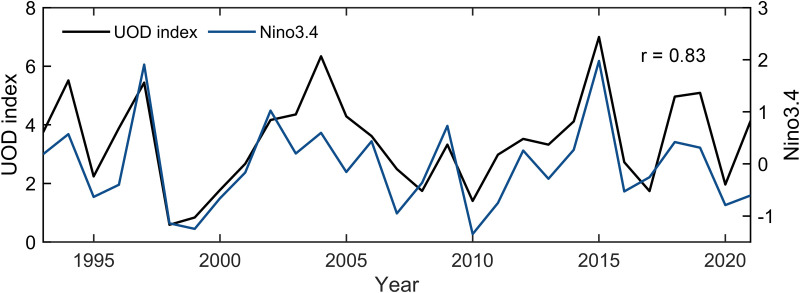
The temporal changes of UOD index and Nino3.4 index.

To better reflect the long-term trend in UOD index, this study adopted the method proposed by [56] to determine the ENSO year. In tropical cyclones active season (June to November), the years in which the monthly mean value of Nino3.4 index was greater than or equal to 0.5 were defined as El Niño years, and less than or equal to −0.5 were defined as La Niña years, the rest of the years were defined as neutral years. According to the method above, it can be determined that from 1993 to 2021, there were 6 El Niño years, 10 La Niña years, and the remaining 13 years were defined as neutral years, as shown in Table 3. The frequent occurrence of El Niño and La Niña events in the three mutation periods led to drastic fluctuations in UOD index (Fig 3). Fig 5 shows a significant interannual correlation between annual cumulative UOD index and Nino3.4 index, up to 0.83. In El Niño years, UOD index was higher, and in La Niña years, UOD index was lower. An interesting finding is that in recent years, the UOD index is significantly higher — especially in La Niña years—than the UOD index in years with a similar level of La Niña intensity in the past.

**Table 3:** ENSO events from 1993–2021.

Year	UOD index	Nino3.4	Year	UOD index	Nino3.4	Year	UOD index	Nino3.4
1993	3.74	0.19	2003	4.36	0.20	2013	3.32	−0.29
1994^+^	5.52	0.57	2004^+^	6.34	0.60	2014	4.12	0.27
1995^−^	2.24	−0.63	2005	4.29	−0.16	2015^+^	7.00	1.98
1996	3.89	−0.40	2006	3.62	0.44	2016^−^	2.73	−0.53
1997^+^	5.44	1.91	2007^-^	2.49	−0.95	2017	1.74	−0.26
1998^-^	0.59	−1.14	2008	1.74	−0.36	2018	4.96	0.42
1999^-^	0.84	−1.25	2009^+^	3.33	0.73	2019	5.09	0.31
2000^-^	1.78	−0.66	2010^-^	1.40	−1.35	2020^-^	1.96	−0.79
2001	2.67	−0.17	2011^-^	2.98	−0.75	2021^-^	4.14	−0.61
2002^+^	4.17	1.02	2012	3.52	0.26			

Note: “+” corresponds to El Niño, and “-” corresponds to La Niña. Nino3.4 index in the table is monthly average from June to November.

## Discussion

### Environmental factors response to ENSO events

**Fig 6:**
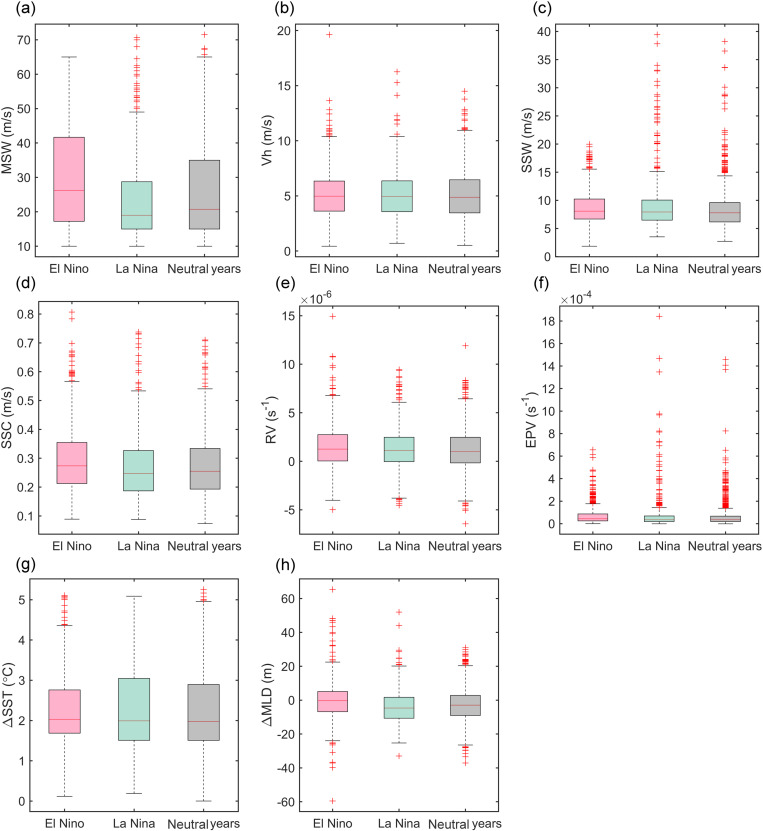
Distribution of environmental factors from 1993 to 2021. Pink bars, green bars and gray bars show the distribution of environmental factors in El Niño years, La Niña years and neutral years, respectively. The red line represents the median, and the “+” represents outliers. (A)-(C) are typhoon intensity attributes, (D)-(F) are ocean dynamic factors, and (G)-(H) are ocean thermal factors.

**Fig 7:**
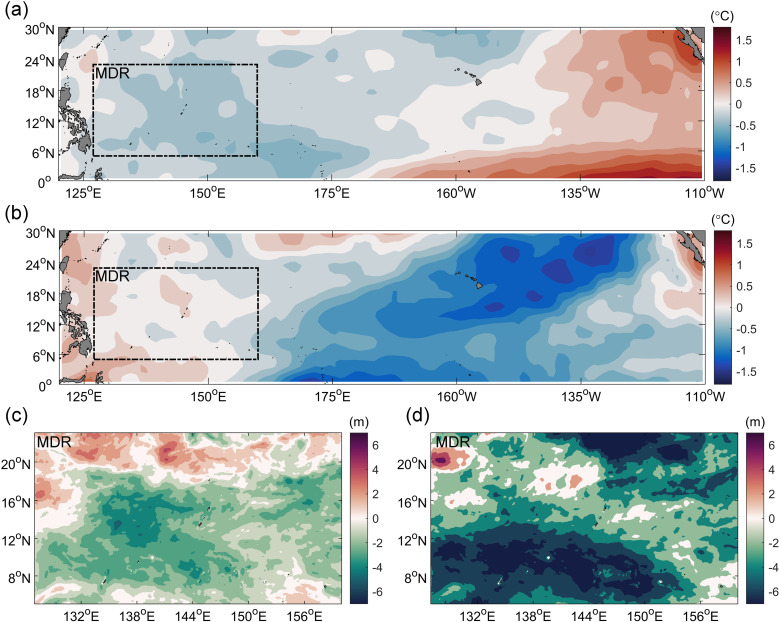
The difference of ocean thermal environment between ENSO years (June–November) and neutral years (June–November). (A) and (B) respectively show the difference distribution of SST in El Niño years, La Niña years and neutral years; (C) and (D) respectively show the difference distribution of MLD in El Niño years, La Niña years and neutral years. The black box represents the MDR area.

There was a significant difference of UOD index in ENSO events, UOD index was larger in El Niño years and smaller in La Niña years. The distribution of each index attribute was shown in Fig 6. The largest variability of TC intensity, ocean dynamic and thermal environmental factors were MSW, SSC and △SST, respectively. The changes of these three environmental factors had a greater impact on UOD index. In El Niño years, the median values of MSW, SSC and △SST were 27 m/s, 0.28 m/s and 2.1°C, respectively, while in La Niña years, the median values of MSW, SSC and △SST were 19 m/s, 0.25 m/s and 2°C, respectively. The high outliers of the environmental factors showed that the maximum values of El Niño years mainly occurred in 1997,2002,2004 and 2015, while in La Niña years, they were mainly concentrated in 2010, 2020 and 2021, and in normal years they appeared most often in the last 10 years. Generally speaking, the values of the environmental factors in El Niño years were higher than those in La Niña years. However, the variability of △SST was abnormally larger in La Niña years, which indicates that the ocean thermal factors played more important roles in La Niña years (Fig 6H) compared with El Niño or neutral years. This was confirmed by the differing distribution of the ocean thermal environment between ENSO years and neutral years shown in Fig 7. In the MDR, compared with the neutral years, the SST was 0.25°C lower and the MLD was 0.64 m lower in the El Niño years, and the SST was 0.07°C higher and the MLD was 2.92 m lower in the La Niña years. The higher SST and shallower MLD in La Niña years led to greater seawater cooling during the passage of TCs, and the ocean thermal environment played a greater role.

### The effect of oceanic thermal factors in recent ten years

**Fig 8:**
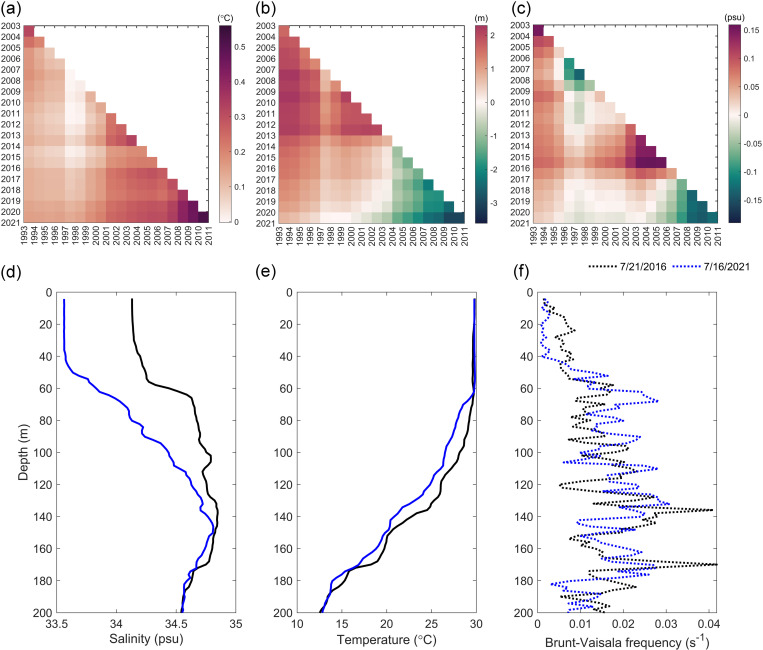
The trend matrices of the annual mean SST (a), MLD (b) and salinity (c) from 1993–2021 with a time interval of at least ten years used to calculate trends (hypotenuse). **(d)-(f) display changes in temperature, salinity, and buoyancy frequencies of Argo floats.** The x-axis and y-axis indicate the start and end year, respectively, and the colors of the rectangles indicate the intensity of the trend (a)-(c).

The role of ocean thermal factors has been gradually increasing in the past 10 years. Sea temperature and salinity are closely related to upper ocean stratification. The trends in SST, MLD and salinity from 1993 to 2021 were analyzed (Fig 8A–8C). These trends were uneven and varied over time. Among them, the trend of SST was increasing, and the maximum increase of SST was 0.6°C during 2011–2021. MLD and salinity showed different trends in different time periods. Before 2004, the MLD trend was deepening, and the trend of MLD became shallower after 2004. The MLD became shallower by 3.5 m from 2011–2020. The salinity of seawater has also been decreasing by about 0.13 psu from 2011 to 2021.The higher temperature and lower salinity of the upper ocean has also changed the upper ocean stratification. Based on Argo floats in two adjacent regions (Fig 8), the thermocline on July 21, 2016, was 97.94 m, the halocline was 57.94 m. Comparatively, the thermocline on July 16, 2021, was 68.12 m, and the halocline was 52.22 m, the MLD became shallower. The ocean barrier layer occurs when the top of the thermocline is deeper than the bottom of the halocline [57–59]. We found that the barrier layer in July 2021 was thinner than that in July 2016, and the thinner barrier layer was conducive to ocean mixing. During the passage of strong TC, the entrainment and vertical mixing with the subsurface cold water at the bottom of the mixed layer strengthened the cooling of SST. Additionally, there has been a weakening trend in ocean stratification above 60 m over the past decade (Fig 8F). Therefore, the higher SST and shallower MLD and barrier layer in the last 10 years led to greater SST cooling than before, especially in La Niña years.

## Conclusions

This paper examined MDR, a key region of TC development in the Western North Pacific, as the research area, selected the TCs that developed in this region (> 1d) from 1993 to 2021, calculated several important attributes, used CRITIC weight method to weight the attributes and established a comprehensive index system, the UOD index which reflected the upper ocean disturbance caused by TCs. The UOD index consisted of TC intensity factors (MSW, V_h_, SSW), ocean dynamic factors (SSC, RV and EPV) and ocean thermal factors (ΔSST, ΔMLD). Based on the analysis of UOD index at different time scales, the upper ocean disturbance changes caused by the TCs from 1993 to 2021 in the MDR were comprehensively displayed. And the increasing role of ocean thermal factors in the past 10 years has also been shown.

The accumulated UOD index of TCs showed that the top 10 TCs generally had a stronger intensity, slower translation speed and a longer stay in the MDR. Significant differences in marine environmental fields also affected UOD index. TCs were affected by cold eddies (warm eddies) tended to strengthen (inhibit) the disturbance of the upper ocean. The monthly and interannual changes of accumulative UOD index were obviously influenced by ENSO. Generally, the UOD index was higher in El Niño years and lower in La Niña years. The M-K breakpoint test of monthly accumulated UOD index showed that the trend of UOD index fluctuated between 1993–2021 (Fig 3) and there were three obvious mutation periods: 1993–1997, 2014–2018 and 2021. In these periods, the events of El Niño and La Niña frequently alternated. WTC and XWT analyses of UOD index and Nino3.4 index showed significant resonance frequencies (16–32 months), which had stable in-phase relationship, and UOD index was ahead of Nino3.4 index in the small periods of 6–14 months (Fig 4). There was a significant interannual correlation between the interannual accumulative UOD index and Nino3.4 index, up to 0.83 (Fig 5). The effect of ocean thermal factors has been increasing gradually in the past 10 years, especially in La Niña years (Fig 6H). Continuously increasing SST, decreasing MLD and seawater salinity have changed the upper ocean stratification. The Argo floats in two adjacent areas showed that in July 2021, compared with July 2016, MLD became shallower and ocean barrier layer became thinner, which was conducive to the SST cooling by entrainment and vertical mixing with the subsurface cold water at the bottom of the mixed layer during the passage of TCs (Fig 8). Therefore, during La Niña years, the influence of ocean thermal factors on ocean disturbances gradually increased. This was due to higher SST and shallower MLD in the MDR region, which made TC-induced forcing more likely to cause significant SST cooling.

## Supporting information

S1 Table Sample of TC activity and UOD index in MDR region from 1993 to 2021. (PDF)

S2 Data Argo float and UOD index data. (ZIP)
