# Supplementary material for: Changes of upper ocean disturbance caused by tropical cyclones in the Western North Pacific main development region (1993–2021)
Source: PLoS One. 2025 Apr 24;20(4):e0320143. doi: 10.1371/journal.pone.0320143 (PMC12021217; doi:10.1371/journal.pone.0320143)
Supplement: S1 Table — (PDF) [file pone.0320143.s001.pdf]

Sample	TC ID	Date	Latitude (°N)	Longitude (°E)	SSW (m/s)	MSW (m/s	EPV (m/s)	V_h (m/s)	RV (s^{-1})	ΔMLD (m)	ΔSST (℃)	SSC (m/s)	UOD index
1	9301	19930313	8.23	157.68	10.89	17.50	0.0001601	8.27	3.67E-06	12.36609	1.08	0.26	0.04013
2	9301	19930314	11.95	152.98	13.47	25.00	7.98E-05	5.90	1.60E-06	4.442669	1.85	0.48	0.05411
3	9301	19930315	14.20	149.98	10.83	25.00	7.14E-05	4.74	9.21E-07	30.95457	2.25	0.23	0.044
4	9301	19930316	17.00	150.90	9.61	25.00	1.22E-05	2.53	2.69E-06	26.92445	2.43	0.26	0.04528
5	9301	19930317	17.73	151.73	6.54	18.33	1.72E-05	1.89	3.57E-07	3.262461	2.68	0.23	0.04045
6	9302	19930616	5.73	157.10	5.94	12.00	7.16E-05	3.31	3.46E-06	5.135693	0.38	0.23	0.02969
7	9302	19930617	6.88	155.63	4.04	15.00	1.96E-05	3.18	2.63E-06	9.112637	0.44	0.17	0.02609
8	9302	19930618	7.60	153.43	3.36	15.00	1.42E-05	4.34	1.01E-06	-0.80217	0.73	0.17	0.026
9	9302	19930619	7.63	149.58	4.42	15.00	3.14E-05	4.86	1.45E-06	-5.25252	0.44	0.19	0.02699
10	9302	19930620	7.30	146.50	4.70	15.00	4.12E-05	2.69	1.08E-06	-3.82956	0.80	0.28	0.03259
11	9302	19930621	8.05	143.38	4.32	16.25	2.38E-05	7.17	2.14E-06	4.192586	1.31	0.28	0.03287
12	9302	19930622	10.40	138.45	4.16	25.00	2.42E-05	6.71	-4.78E-08	-11.3189	1.86	0.37	0.0429
13	9302	19930623	12.43	133.73	5.13	40.00	2.43E-05	5.86	1.33E-06	-4.86336	1.94	0.25	0.04909
14	9302	19930624	13.85	129.23	4.85	56.25	2.37E-05	6.40	2.21E-06	-3.56314	1.25	0.23	0.05477
15	9304	19930719	13.80	149.70	7.09	13.50	9.37E-06	5.10	-2.39E-07	-3.39916	2.25	0.14	0.03187
16	9304	19930720	15.55	146.98	8.75	20.00	1.55E-05	6.33	-1.73E-07	-7.71626	2.20	0.17	0.03743
17	9304	19930721	18.65	142.58	8.13	25.00	1.58E-05	6.13	1.85E-06	-4.40717	3.16	0.44	0.05365
18	9304	19930722	21.25	140.43	6.37	25.00	8.89E-06	2.57	7.18E-07	-2.95038	4.05	0.24	0.05056
19	9304	19930723	22.50	140.00	7.91	25.00	1.45E-05	2.26	3.05E-06	-2.82089	4.56	0.32	0.05802
20	9305	19930724	16.80	138.50	9.58	12.00	8.90E-06	8.83	-2.82E-06	-13.8251	2.49	0.37	0.03854
21	9305	19930725	20.75	136.98	10.71	16.25	1.57E-05	8.45	-3.96E-06	-3.04448	3.32	0.33	0.04277
22	9307	19930801	7.43	153.20	5.50	14.25	3.50E-05	6.41	2.21E-06	-7.17159	0.77	0.27	0.03128
23	9307	19930802	9.28	148.05	5.69	17.50	2.83E-05	7.44	1.84E-06	-12.7884	0.90	0.27	0.03325
24	9307	19930803	10.30	143.18	9.68	26.25	9.739E-05	4.65	3.04E-06	-10.5938	1.71	0.23	0.04664
25	9307	19930804	10.98	140.73	12.55	30.00	0.0001514	2.94	5.14E-06	-17.7238	1.82	0.43	0.06193
26	9307	19930805	13.73	139.38	9.57	31.25	9.55E-05	5.78	2.18E-06	-12.5391	2.03	0.19	0.04868
27	9307	19930806	17.10	135.98	11.48	36.25	1.31E-04	6.58	-9.28E-07	-9.70897	1.51	0.31	0.05311
28	9307	19930807	20.58	132.30	11.95	43.75	0.0001239	5.83	3.86E-06	-0.34519	2.31	0.25	0.06147
29	9308	19930805	12.20	152.50	8.72	10.00	9.15E-06	2.95	-2.48E-07	7.765944	1.96	0.14	0.02967
30	9308	19930806	13.20	150.88	3.34	11.50	1.26E-06	4.37	-1.90E-07	8.929314	2.25	0.15	0.02808
31	9308	19930807	14.90	147.75	3.49	15.00	6.26E-06	4.55	-8.10E-07	-1.70827	2.00	0.13	0.029
32	9308	19930808	15.60	144.35	4.67	20.00	4.94E-06	4.60	1.95E-06	-4.74441	1.88	0.16	0.03468
33	9308	19930809	16.18	140.55	4.93	25.00	1.36E-05	4.39	1.52E-06	-0.64825	2.23	0.14	0.03833
34	9308	19930810	16.95	137.38	6.44	30.00	8.97E-06	4.56	-3.18E-06	9.936001	2.23	0.40	0.04687
35	9308	19930811	19.15	134.33	5.85	25.00	1.63E-05	5.35	3.41E-06	0.767201	2.73	0.33	0.04776
36	9308	19930812	22.17	131.60	6.39	16.67	1.38E-05	6.07	-3.58E-07	-2.75885	3.32	0.29	0.04239
37	9309	19930814	9.38	137.63	3.77	10.00	1.07E-05	11.06	-5.95E-07	-3.24724	1.49	0.24	0.02525
38	9309	19930815	11.55	130.73	3.29	11.50	8.07E-06	8.78	-1.81E-06	-11.2402	1.47	0.37	0.03058
39	9309	19930816	15.53	127.77	4.56	14.00	1.29E-05	4.79	-7.14E-08	-10.6942	1.11	0.10	0.02511
40	9311	19930821	18.70	153.50	6.55	15.00	1.55E-05	1.42	3.96E-07	-9.98744	2.93	0.18	0.03926
41	9311	19930822	19.45	152.95	7.88	18.75	1.83E-05	2.46	-6.93E-07	-9.6483	3.29	0.18	0.04265
42	9311	19930823	21.85	151.05	9.46	22.50	2.08E-05	5.30	2.09E-06	-1.19849	4.86	0.38	0.05897
43	9313	19930829	19.60	136.18	4.21	13.50	7.03E-06	4.93	1.86E-06	-13.6173	3.01	0.18	0.03659
44	9313	19930830	20.58	132.38	6.68	20.00	2.57E-05	6.20	-4.10E-07	-8.48558	2.33	0.19	0.03768
45	9313	19930831	20.63	128.17	6.25	23.33	2.96E-05	9.78	-1.41E-06	-8.78845	3.06	0.20	0.04091
46	9314	19930905	20.25	130.53	4.08	12.50	8.32E-06	2.48	5.41E-07	-18.6621	2.57	0.20	0.0353
47	9314	19930906	21.40	128.58	5.65	17.50	6.64E-06	3.60	-9.60E-07	2.812101	3.28	0.19	0.03916
48	9316	19930913	13.87	129.77	4.28	12.00	1.20E-05	4.91	2.08E-06	-2.0796	1.48	0.23	0.03041
49	9316	19930914	15.25	127.60	5.54	15.00	1.10E-05	4.73	-1.71E-06	6.370993	1.10	0.18	0.02682
50	9317	19930923	13.78	149.75	5.74	16.75	1.81E-05	1.47	6.47E-07	-8.0045	2.25	0.15	0.03578
51	9317	19930924	15.98	148.28	8.25	26.25	4.59E-05	7.23	-2.99E-07	-5.05675	2.26	0.18	0.0415
52	9317	19930925	21.23	145.55	10.03	41.25	6.70E-05	6.55	2.99E-06	8.160788	4.06	0.33	0.06581
53	9319	19930930	13.18	145.33	4.50	14.25	1.80E-05	4.12	2.42E-06	2.098685	1.75	0.31	0.03612
54	9319	19931001	14.30	142.33	5.53	23.75	2.96E-05	3.78	5.21E-07	7.165227	1.87	0.20	0.03798
55	9319	19931002	15.93	139.18	6.02	36.25	3.10E-05	5.66	1.22E-06	-5.82209	2.27	0.26	0.04978
56	9319	19931003	18.10	135.45	8.66	40.00	4.10E-05	4.34	-4.72E-07	-11.9899	1.69	0.20	0.04925
57	9319	19931004	19.70	132.95	9.55	48.75	5.40E-05	2.82	-1.97E-06	-16.4244	2.54	0.39	0.06511
58	9319	19931005	21.90	133.00	12.07	51.25	1.06E-04	3.81	1.59E-06	-7.60623	3.15	0.41	0.07389
59	9320	19931001	16.43	129.03	4.81	12.75	1.62E-05	3.38	-1.78E-06	-5.92753	1.06	0.16	0.02594
60	9320	19931002	16.60	127.30	4.49	20.00	1.49E-05	3.46	2.12E-06	-3.1406	1.25	0.18	0.03336
61	9320	19931007	22.60	127.60	10.61	25.00	6.87E-05	3.46	-1.54E-08	-3.62672	3.33	0.26	0.0519
62	9321	19931006	9.00	142.60	8.32	10.00	3.67E-05	7.58	1.10E-06	-10.0411	1.65	0.13	0.02887
63	9321	19931007	11.25	139.05	6.40	12.25	3.49E-05	8.02	6.04E-07	7.592751	1.76	0.28	0.03252
64	9321	19931008	15.18	135.68	7.07	15.00	3.87E-05	7.49	2.69E-06	9.446963	1.69	0.24	0.03425
65	9321	19931009	20.25	133.00	7.83	20.00	2.58E-05	7.49	-1.23E-06	3.43321	2.65	0.25	0.03964
66	9322	19931019	13.05	159.75	4.31	12.00	1.48E-05	3.11	-1.47E-07	-7.01468	2.59	0.23	0.03506
67	9322	19931020	13.85	157.78	5.51	15.00	1.84E-05	4.00	1.12E-06	-13.1402	2.85	0.16	0.03731
68	9322	19931021	15.90	156.03	7.55	15.00	2.81E-05	3.18	1.26E-06	-17.6936	2.72	0.15	0.03859
69	9322	19931022	19.88	155.13	8.15	20.00	2.46E-05	8.44	-7.07E-07	-15.4924	2.93	0.28	0.04335
70	9323	19931027	12.50	145.25	3.93	12.00	1.58E-05	7.56	5.02E-07	-2.52998	1.69	0.18	0.0276
71	9323	19931028	13.00	140.30	6.17	18.75	2.34E-05	8.48	5.66E-07	3.447066	1.99	0.28	0.03681
72	9323	19931029	13.48	134.33	5.04	27.50	2.44E-05	7.18	3.88E-07	8.18463	1.98	0.29	0.04166
73	9323	19931030	14.70	129.00	7.86	36.25	5.55E-05	5.31	1.36E-06	-1.75965	1.27	0.26	0.04716
74	9324	19931106	12.35	148.50	4.94	15.00	8.25E-06	6.14	-1.56E-06	-2.89315	1.80	0.18	0.02965
75	9324	19931107	13.43	144.55	4.10	15.00	1.08E-05	6.15	1.38E-06	-1.73467	1.78	0.18	0.03061
76	9324	19931108	14.78	140.13	4.96	15.00	1.53E-05	8.06	-3.86E-08	10.67565	1.89	0.17	0.029
77	9324	19931109	18.30	136.20	8.34	20.00	1.59E-05	3.10	8.02E-07	25.9153	1.95	0.28	0.03929
78	9324	19931110	20.80	135.95	10.29	23.75	1.37E-05	4.29	-4.98E-07	23.26878	3.70	0.30	0.04955
79	9324	19931111	22.05	138.60	7.72	23.75	7.44E-06	4.55	1.20E-06	17.69093	3.90	0.34	0.05132
80	9324	19931112	22.33	141.47	7.80	18.33	6.42E-06	2.70	2.83E-06	17.68802	4.71	0.15	0.04729
81	9326	19931201	7.20	145.50	4.22	10.00	1.55E-05	8.69	3.70E-07	-3.06101	0.93	0.09	0.01978
82	9326	19931202	9.15	141.35	5.66	13.00	2.35E-05	10.93	2.17E-06	-3.09848	1.81	0.33	0.03439
83	9326	19931203	11.93	133.25	6.45	22.50	2.44E-05	8.79	-6.32E-07	3.142389	1.89	0.41	0.04213
84	9326	19931204	13.60	128.70	6.38	30.00	3.49E-05	7.66	4.92E-07	12.50199	1.16	0.21	0.03749
85	9327	19931204	8.10	143.35	5.40	12.00	3.66E-05	8.68	1.09E-06	1.404328	1.34	0.23	0.02899
86	9327	19931205	9.70	138.18	7.16	26.25	3.33E-05	8.67	1.53E-06	7.802389	1.74	0.47	0.04709
87	9327	19931206	12.88	132.98	6.43	30.00	2.61E-05	6.79	3.11E-07	12.75843	1.92	0.26	0.04242
88	9327	19931207	15.80	129.90	8.33	30.00	3.62E-05	2.93	3.50E-07	1.81813	1.47	0.21	0.04258
89	9327	19931208	16.13	129.85	7.31	35.00	2.60E-05	3.59	2.99E-07	-9.86633	1.38	0.20	0.04424
90	9327	19931209	14.30	128.10	8.39	37.50	1.88E-05	5.86	-4.99E-07	9.445903	1.05	0.31	0.04539
91	9328	19931223	7.50	139.50	5.00	12.00	2.72E-05	4.85	1.53E-06	2.71102	1.71	0.23	0.03161
92	9328	19931224	8.10	136.40	7.36	13.50	4.33E-05	7.85	9.45E-07	4.664405	1.24	0.44	0.03762
93	9328	19931225	8.73	130.33	8.48	22.50	6.09E-05	7.80	6.68E-08	6.042506	1.28	0.50	0.04536
94	9401	19940331	10.95	134.35	6.38	13.50	1.91E-05	4.74	-6.26E-07	9.234234	1.74	0.38	0.0365
95	9401	19940401	11.48	131.95	5.87	18.75	1.85E-05	3.09	-6.49E-07	-3.56632	1.65	0.36	0.03949
96	9401	19940402	11.45	129.58	8.40	20.00	3.19E-05	3.17	4.99E-07	-0.84297	1.41	0.38	0.0423
97	9401	19940403	10.67	127.63	8.48	25.00	5.56E-05	3.04	2.07E-06	10.36914	1.31	0.42	0.04711
98	9402	19940511	11.70	140.50	6.75	15.00	2.04E-05	6.14	-3.77E-07	-0.29094	1.86	0.30	0.03582
99	9402	19940512	12.40	137.93	6.01	15.00	3.06E-05	5.13	1.33E-06	-8.90309	1.84	0.24	0.03557

100	9402	19940513	14.68	135.45	7.43	18.75	3.66E-05	3.36	2.60E-06	-11.9353	1.81	0.22	0.03974
101	9402	19940514	17.28	135.90	7.42	26.25	3.00E-05	4.68	2.83E-06	-0.44241	1.48	0.33	0.04468
102	9402	19940515	20.90	137.63	6.48	35.00	2.59E-05	5.55	-8.01E-07	0.203457	3.11	0.17	0.04817
103	9406	19940707	13.80	130.73	10.38	15.00	8.67E-06	4.50	1.19E-06	-8.39789	1.82	0.12	0.03339
104	9406	19940708	16.45	128.85	7.63	25.00	2.85E-05	4.28	5.83E-07	-9.20297	1.04	0.17	0.03624
105	9408	19940714	11.67	128.00	5.71	15.00	2.34E-05	10.82	2.05E-06	-7.13523	1.37	0.28	0.03238
106	9408	19940717	16.57	128.83	6.91	26.67	4.32E-05	4.60	8.37E-07	-12.8071	1.03	0.23	0.03936
107	9408	19940718	19.03	131.10	7.46	35.00	3.50E-05	5.13	2.90E-06	-10.7225	2.41	0.35	0.05496
108	9408	19940719	21.70	133.10	6.63	48.33	2.87E-05	4.91	3.96E-06	-0.15127	3.07	0.36	0.06505
109	9412	19940726	15.40	128.87	5.95	15.00	2.75E-05	3.80	6.50E-07	-26.4284	1.21	0.31	0.03621
110	9412	19940727	16.00	127.40	6.77	15.00	3.31E-05	10.94	2.74E-06	-11.832	1.12	0.25	0.03183
111	9412	19940728	18.10	127.00	6.52	15.00	2.38E-05	4.25	1.74E-09	-8.55764	1.52	0.16	0.03109
112	9412	19940729	20.10	128.25	5.21	20.00	1.94E-05	5.44	-1.18E-06	4.511908	2.60	0.33	0.04139
113	9412	19940730	22.70	129.00	4.89	20.00	1.61E-05	19.63	-1.50E-07	-1.85553	2.94	0.34	0.03782
114	9414	19940801	14.87	143.33	3.20	12.00	6.98E-06	3.75	9.13E-08	-7.80977	1.87	0.17	0.02911
115	9414	19940802	15.25	140.90	5.65	15.00	1.61E-05	2.82	-1.71E-06	-16.5286	1.93	0.19	0.03328
116	9414	19940803	15.43	138.53	8.10	23.75	6.606E-05	4.36	3.81E-06	-13.5376	2.22	0.19	0.04503
117	9414	19940804	15.15	134.55	12.31	35.00	0.0001206	5.07	-1.02E-06	-0.37857	1.75	0.34	0.05453
118	9414	19940805	16.10	130.33	15.75	48.75	0.0002402	6.58	3.67E-06	2.953302	1.73	0.43	0.0732
119	9414	19940806	17.50	127.30	15.54	50.00	0.0002605	5.99	3.43E-06	8.811457	1.52	0.55	0.0772
120	9417	19940813	17.40	148.00	3.74	12.00	1.08E-05	4.94	-8.97E-07	-0.61164	2.58	0.23	0.0329
121	9417	19940814	17.68	145.43	5.11	15.00	2.15E-05	5.40	1.37E-06	3.769778	2.85	0.18	0.03626
122	9417	19940815	18.30	141.15	6.28	18.75	2.88E-05	4.26	2.67E-06	-0.59765	3.28	0.25	0.04499
123	9417	19940816	17.75	137.83	7.64	28.75	4.12E-05	5.12	8.33E-07	4.427219	2.52	0.31	0.04855
124	9417	19940817	18.25	133.58	9.44	36.25	6.21E-05	5.02	4.72E-06	7.738986	1.96	0.44	0.05845
125	9417	19940818	19.88	130.23	8.00	42.50	4.30E-05	4.94	8.26E-07	4.176691	2.42	0.17	0.05169
126	9417	19940819	21.50	127.50	7.41	52.50	4.17E-05	5.70	-1.51E-07	-3.71203	3.31	0.24	0.06306
127	9418	19940821	22.60	157.30	1.85	10.00	-2.13E-08	5.70	-4.78E-07	4.291136	3.37	0.23	0.03345
128	9418	19940828	22.37	143.10	8.90	18.33	9.44E-06	6.06	1.80E-07	-2.85846	5.01	0.34	0.05399
129	9418	19940829	21.63	138.65	10.07	15.00	1.86E-05	7.08	-5.00E-06	3.750174	3.69	0.47	0.04772
130	9418	19940830	22.03	132.40	7.85	22.50	2.14E-05	8.03	3.55E-06	6.1898	3.10	0.28	0.04629
131	9418	19940831	22.30	128.10	7.66	30.00	3.07E-05	9.56	-4.02E-06	5.2255	3.29	0.31	0.04756
132	9424	19940906	13.75	132.00	2.93	12.00	8.37E-06	5.07	1.31E-06	-8.46754	2.00	0.19	0.03024
133	9424	19940907	14.95	130.03	5.07	15.00	1.48E-05	3.39	-1.66E-07	-5.3675	1.71	0.25	0.03375
134	9424	19940908	16.27	128.23	5.82	18.33	1.32E-05	3.82	1.46E-06	-3.03596	1.06	0.44	0.04005
135	9425	19940911	10.25	159.05	6.75	15.00	5.45E-05	5.72	2.20E-06	-3.90713	2.13	0.22	0.03718
136	9425	19940912	11.85	157.05	4.07	22.50	1.73E-05	3.34	1.87E-06	-6.10927	2.27	0.32	0.04345
137	9425	19940913	13.70	158.00	6.29	26.25	3.62E-05	3.15	8.42E-07	-0.73855	2.81	0.14	0.04323
138	9425	19940914	15.83	159.33	8.73	40.00	5.08E-05	4.23	3.04E-06	-1.35717	2.77	0.21	0.05599
139	9425	19940915	19.68	159.50	11.99	52.50	8.41E-05	6.46	8.87E-07	-8.58918	2.96	0.26	0.06669
140	9425	19940916	22.80	158.40	11.19	60.00	7.83E-05	7.46	9.58E-07	-0.57779	3.84	0.48	0.08029
141	9426	19940915	14.73	143.63	5.89	12.75	2.24E-05	5.67	8.82E-07	-5.52221	1.82	0.12	0.02911
142	9426	19940916	14.85	148.40	9.05	18.75	3.88E-05	6.36	-5.54E-07	4.616455	2.05	0.23	0.03789
143	9426	19940917	17.63	154.20	8.13	20.00	1.63E-05	9.64	8.56E-07	-3.62301	2.62	0.16	0.0373
144	9426	19940918	21.77	155.97	6.79	20.00	1.41E-05	4.95	-3.79E-06	3.295045	2.96	0.24	0.03953
145	9427	19940917	13.73	136.27	6.19	14.00	1.05E-05	6.79	3.50E-07	9.798508	1.86	0.21	0.03102
146	9427	19940918	12.48	140.38	7.38	15.00	1.70E-05	4.96	-8.88E-08	-3.18722	1.95	0.33	0.03816
147	9427	19940919	13.08	142.50	4.97	17.50	1.81E-05	3.86	-4.34E-07	-11.1194	1.70	0.33	0.03783
148	9427	19940920	16.05	141.95	5.44	21.25	1.85E-05	4.90	-6.40E-07	-4.75156	2.17	0.15	0.03557
149	9427	19940921	18.58	140.98	6.06	26.25	2.64E-05	3.94	5.41E-07	-6.55963	3.41	0.17	0.04625
150	9427	19940922	17.68	138.48	7.56	30.00	2.48E-05	2.30	6.59E-07	4.778319	2.64	0.21	0.04697
151	9427	19940923	17.70	137.83	7.29	37.50	4.39E-05	2.58	-3.12E-07	-7.18112	2.51	0.20	0.05123
152	9427	19940924	19.30	136.83	7.96	51.25	4.35E-05	1.85	-2.27E-06	-5.15424	2.81	0.22	0.0603
153	9427	19940925	21.00	136.45	8.44	55.00	5.61E-05	3.31	3.79E-06	-8.5228	3.61	0.49	0.07843
154	9427	19940926	22.50	135.60	8.60	55.00	5.19E-05	3.89	5.68E-06	-11.5569	4.26	0.26	0.07499
155	9429	19940923	19.00	154.00	10.99	12.00	1.18E-05	5.22	2.57E-06	-6.3686	2.87	0.21	0.03998
156	9429	19940924	21.38	154.43	6.29	15.00	2.47E-05	6.02	-3.43E-06	-7.42326	3.12	0.22	0.03704
157	9430	19941002	11.90	158.75	4.05	12.00	7.57E-06	12.43	7.76E-07	8.256783	2.41	0.25	0.03025
158	9430	19941003	10.93	153.13	2.55	18.75	8.00E-06	7.80	1.78E-06	3.010524	1.37	0.37	0.03556
159	9430	19941004	11.45	146.83	4.65	23.75	1.60E-05	8.01	-3.80E-07	3.006285	1.01	0.18	0.03064
160	9430	19941005	12.70	140.28	6.58	26.25	4.57E-05	9.23	2.15E-06	7.835204	1.97	0.24	0.04105
161	9430	19941006	14.68	133.78	8.44	35.00	6.23E-05	8.10	-3.67E-08	10.48332	1.92	0.28	0.04756
162	9430	19941007	16.93	128.57	11.19	45.00	9.315E-05	7.16	2.39E-06	-4.05748	1.11	0.37	0.05809
163	9431	19941016	15.20	145.33	4.27	10.00	8.14E-06	8.93	-1.03E-06	-0.84165	1.98	0.19	0.02645
164	9431	19941017	16.23	139.33	5.34	16.25	1.25E-05	6.51	1.93E-06	-1.73865	2.31	0.20	0.03516
165	9431	19941018	15.53	133.80	7.70	23.75	2.34E-05	6.98	-2.01E-06	-5.34153	1.86	0.32	0.04111
166	9431	19941019	15.53	128.87	10.44	31.67	2.86E-05	6.31	-8.73E-07	7.91203	1.19	0.32	0.04446
167	9432	19941016	11.40	158.70	4.09	15.00	1.08E-05	11.09	6.80E-07	-3.42433	2.29	0.25	0.03283
168	9432	19941017	13.33	154.13	8.00	15.00	1.20E-05	7.81	2.51E-06	4.300143	2.28	0.33	0.03941
169	9432	19941018	14.28	148.38	6.64	18.75	2.07E-05	7.69	-1.25E-06	7.636781	2.02	0.27	0.03565
170	9432	19941019	14.78	142.73	5.78	26.25	3.56E-05	7.27	1.08E-06	6.20861	1.88	0.11	0.03573
171	9432	19941020	16.08	136.78	6.09	35.00	3.45E-05	6.53	-3.22E-06	-6.49499	1.84	0.21	0.04286
172	9432	19941021	17.48	132.65	6.91	40.00	3.88E-05	3.62	3.86E-06	-2.20941	2.00	0.24	0.05277
173	9432	19941022	18.43	131.30	8.78	40.00	5.07E-05	1.45	-1.92E-06	-2.2465	2.21	0.34	0.05622
174	9432	19941023	18.55	130.23	9.92	45.00	7.51E-05	1.23	-1.68E-06	-10.5482	1.99	0.26	0.05764
175	9432	19941024	17.43	129.70	10.90	45.00	0.0001145	2.28	3.34E-06	-8.52478	1.30	0.47	0.06575
176	9432	19941025	16.00	129.45	13.55	45.00	0.0001953	1.59	6.24E-06	2.669352	1.24	0.48	0.07153
177	9432	19941026	15.25	128.85	14.48	40.00	0.0001661	2.13	5.23E-06	-0.9331	1.22	0.43	0.06574
178	9432	19941027	16.10	129.70	13.41	32.50	0.0001808	3.50	1.08E-05	-1.2822	1.31	0.55	0.06837
179	9432	19941028	18.48	130.95	10.70	26.25	1.05E-04	2.93	1.75E-06	0.136168	2.17	0.28	0.05047
180	9432	19941029	20.80	132.08	9.45	25.00	7.47E-05	5.76	2.23E-06	-17.2607	2.37	0.29	0.04943
181	9432	19941030	23.00	134.40	8.19	25.00	4.50E-05	8.41	4.11E-06	-14.5266	4.10	0.26	0.05399
182	9433	19941021	14.63	159.00	8.28	23.33	1.23E-05	4.06	-1.44E-06	1.7922	2.99	0.23	0.04382
183	9433	19941022	16.00	156.05	9.13	31.25	9.85E-06	5.85	1.71E-06	3.914762	2.71	0.23	0.04829
184	9433	19941023	16.83	151.45	6.95	42.50	2.08E-05	5.37	7.98E-07	0.316047	2.41	0.18	0.05086
185	9433	19941024	15.93	148.00	5.34	45.00	2.67E-05	2.34	1.68E-06	14.55809	2.27	0.13	0.04996
186	9433	19941025	16.03	147.40	9.11	45.00	9.999E-05	2.48	2.86E-06	-9.12085	2.35	0.26	0.06151
187	9433	19941026	18.53	149.10	10.79	40.00	0.0001289	4.25	2.78E-06	-7.05502	2.56	0.22	0.0594
188	9433	19941027	20.80	150.73	12.86	40.00	0.0001341	3.56	5.15E-07	-9.9768	3.97	0.28	0.06798
189	9433	19941028	22.65	151.55	10.31	40.00	8.05E-05	2.61	2.92E-06	-4.2763	5.02	0.30	0.07146
190	9435	19941030	11.30	159.20	7.84	15.00	1.83E-05	7.86	9.95E-07	2.561753	2.39	0.25	0.03692
191	9435	19941031	10.40	155.13	7.86	16.25	2.28E-05	5.70	4.36E-07	2.854223	1.55	0.35	0.03782
192	9435	19941101	10.03	151.25	5.40	25.00	4.00E-05	4.63	2.69E-06	10.25295	0.72	0.27	0.03707
193	9435	19941102	12.73	148.53	6.70	30.00	4.74E-05	6.38	-1.75E-07	34.94052	1.95	0.41	0.04666
194	9435	19941103	16.63	145.28	8.76	40.00	6.52E-05	6.16	-2.11E-06	8.216685	2.41	0.17	0.0491
195	9435	19941104	18.40	140.78	8.78	52.50	6.23E-05	6.30	1.75E-06	8.496968	3.40	0.26	0.06557
196	9435	19941105	20.45	135.93	11.90	58.75	9.25E-05	6.73	3.13E-07	19.84233	3.56	0.22	0.06941
197	9435	19941106	22.70	133.70	11.58	55.00	0.000105	5.00	4.68E-06	-10.211	3.75	0.45	0.08136
198	9436	19941215	7.00	147.00	11.20	15.00	4.25E-05	4.22	1.98E-06	-0.08472	0.69	0.34	0.03754
199	9436	19941216	7.68	144.58	10.42	15.00	2.60E-05	6.02	1.53E-06	4.43896	1.08	0.42	0.03945

200	9436	19941217	9.15	140.18	12.33	20.00	1.81E-05	5.13	-2.51E-08	12.6178	1.85	0.26	0.04006
201	9436	19941218	10.48	136.75	12.93	27.50	5.30E-05	4.21	-4.76E-07	15.93066	1.72	0.54	0.05443
202	9436	19941219	11.33	133.28	12.45	35.00	5.59E-05	5.35	1.19E-06	5.653607	1.84	0.40	0.0556
203	9436	19941220	11.20	129.10	11.33	42.50	3.64E-05	5.46	-1.62E-07	5.646189	1.39	0.52	0.05966
204	9437	19941219	7.40	159.80	11.43	20.00	0.0002376	6.23	2.97E-06	-4.77752	1.15	0.26	0.04651
205	9437	19941220	10.20	159.23	10.35	20.00	0.0001257	6.30	2.19E-06	13.43874	2.16	0.36	0.04783
206	9437	19941221	13.00	154.93	10.54	20.00	7.36E-05	8.08	1.88E-06	22.20247	2.26	0.31	0.04338
207	9437	19941222	14.85	149.18	7.47	25.00	5.54E-05	5.72	6.82E-07	47.03295	2.12	0.26	0.04043
208	9437	19941223	16.40	144.98	9.25	25.00	6.08E-05	6.33	-1.07E-06	47.08461	2.29	0.19	0.03864
209	9437	19941224	17.98	140.23	11.21	23.75	3.77E-05	6.39	3.00E-06	39.93024	3.14	0.34	0.04976
210	9437	19941225	19.55	136.18	6.89	16.25	2.00E-05	3.66	-2.05E-06	39.22953	2.98	0.22	0.03614
211	9437	19941226	21.50	135.23	7.39	12.00	1.89E-05	10.27	2.15E-06	-30.7683	4.01	0.20	0.04209
212	9501	19950502	10.65	158.70	7.78	10.00	1.94E-05	4.90	5.37E-07	28.66939	2.11	0.30	0.03336
213	9501	19950503	11.88	156.80	7.00	10.00	1.14E-05	3.34	1.31E-07	1.322469	2.26	0.22	0.03301
214	9501	19950504	12.30	155.20	6.64	10.00	2.12E-05	3.56	-1.19E-06	-2.68573	2.16	0.18	0.03078
215	9503	19950716	17.00	141.55	5.48	13.50	6.66E-06	3.32	-1.48E-06	-1.44429	2.59	0.30	0.03755
216	9503	19950717	18.28	140.05	7.82	16.50	1.28E-05	3.44	5.81E-07	3.21769	3.21	0.27	0.0432
217	9503	19950718	19.00	137.33	6.95	22.75	1.96E-05	3.62	2.13E-06	-1.16377	2.70	0.25	0.04467
218	9503	19950719	20.45	134.08	5.64	30.00	2.05E-05	5.55	-1.23E-06	-10.6198	3.39	0.31	0.05107
219	9503	19950720	22.10	130.63	5.56	30.00	2.00E-05	5.73	7.36E-06	-3.74037	3.29	0.31	0.05503
220	9505	19950807	16.03	131.93	6.26	12.00	8.35E-06	6.79	1.70E-06	2.341077	2.62	0.12	0.03159
221	9505	19950808	18.30	128.63	7.06	15.00	1.27E-05	7.72	1.58E-06	-0.92403	1.32	0.17	0.02955
222	9507	19950820	20.20	131.00	4.68	12.00	1.22E-05	2.47	2.60E-06	-6.00579	2.48	0.37	0.04123
223	9507	19950821	20.05	130.08	4.85	16.50	1.74E-05	1.73	1.92E-06	-8.50809	2.48	0.25	0.04009
224	9507	19950822	21.05	129.35	5.73	20.00	2.11E-05	4.48	6.61E-07	-3.45135	2.94	0.28	0.04337
225	9509	19950824	11.00	132.50	3.61	10.00	1.53E-05	5.67	-6.13E-07	1.534227	1.70	0.37	0.03246
226	9509	19950825	12.95	130.33	5.03	11.50	1.98E-05	5.65	2.51E-07	-1.04775	1.61	0.21	0.02917
227	9509	19950826	15.43	127.97	6.11	19.33	2.35E-05	4.43	4.34E-07	-5.28166	1.12	0.19	0.03243
228	9512	19950912	16.38	146.93	8.28	15.00	1.79E-05	3.66	3.76E-07	-7.22775	2.46	0.15	0.03618
229	9512	19950913	19.00	144.23	10.08	23.75	3.98E-05	7.04	-2.52E-06	-4.59227	3.12	0.39	0.05023
230	9512	19950914	21.25	139.83	9.06	38.75	4.84E-05	5.19	2.49E-06	-3.48979	3.90	0.32	0.06322
231	9512	19950915	23.00	138.00	9.21	50.00	4.65E-05	4.59	6.56E-06	-2.95907	4.14	0.45	0.0776
232	9513	19950916	18.68	130.38	7.80	23.00	3.91E-05	9.00	9.35E-07	-6.70003	2.12	0.15	0.03757
233	9513	19950917	19.78	136.90	10.72	25.75	5.00E-05	5.18	-6.60E-07	-1.80382	2.97	0.19	0.04678
234	9513	19950918	22.00	137.97	12.69	33.33	6.43E-05	5.21	4.75E-06	1.739301	3.51	0.52	0.06859
235	9515	19950927	7.93	134.38	3.81	12.25	1.23E-05	7.36	1.49E-06	-5.50442	1.40	0.26	0.02985
236	9515	19950928	9.50	129.78	6.31	17.00	5.41E-05	5.57	9.36E-07	-14.1764	1.39	0.26	0.03629
237	9515	19950929	10.70	127.30	6.25	23.00	3.73E-05	6.42	1.93E-06	4.718183	1.27	0.20	0.03551
238	9517	19951009	20.05	145.65	5.07	13.50	8.33E-06	3.95	2.43E-06	-5.14232	3.62	0.13	0.03832
239	9517	19951010	22.05	143.80	7.77	15.00	1.04E-05	5.99	2.57E-06	9.507099	5.09	0.18	0.04711
240	9517	19951014	22.30	137.95	4.20	15.00	1.09E-05	3.92	2.72E-06	-6.06688	3.68	0.27	0.04375
241	9518	19951016	12.90	151.97	8.21	14.00	2.11E-05	8.79	-7.13E-07	-4.80137	2.14	0.28	0.03538
242	9518	19951017	14.28	146.08	8.32	20.25	1.83E-05	8.31	3.53E-07	3.081463	1.81	0.16	0.034
243	9518	19951018	16.50	140.05	7.39	36.25	2.58E-05	8.00	2.54E-07	11.55609	2.37	0.23	0.04689
244	9518	19951019	20.28	135.30	9.40	43.75	1.80E-05	6.16	1.07E-06	13.52193	3.57	0.26	0.05929
245	9518	19951020	22.80	134.20	10.91	50.00	2.24E-05	4.71	-4.12E-06	12.16475	4.02	0.21	0.06195
246	9520	19951025	7.55	135.80	3.76	13.00	2.35E-05	4.48	1.99E-06	-2.1408	1.42	0.24	0.03119
247	9520	19951026	8.50	132.83	6.60	15.00	3.22E-05	3.93	1.61E-06	-0.43896	1.40	0.44	0.04054
248	9520	19951027	10.08	129.08	5.90	21.50	1.41E-05	7.12	7.21E-07	7.902758	1.36	0.37	0.03843
249	9521	19951026	10.78	143.88	5.22	13.50	6.98E-06	4.93	-2.80E-07	2.087021	1.70	0.19	0.02949
250	9521	19951027	12.78	140.50	6.35	20.00	6.47E-06	4.04	5.57E-08	2.238787	1.99	0.10	0.03294
251	9521	19951028	13.08	138.30	5.75	21.25	1.18E-05	1.50	2.65E-07	-3.74885	1.86	0.19	0.03746
252	9521	19951029	12.48	137.88	4.92	25.00	1.71E-05	3.17	1.46E-06	-5.44618	1.84	0.14	0.0374
253	9521	19951030	11.88	135.60	6.18	28.75	3.88E-05	3.78	1.31E-06	-3.54698	1.82	0.24	0.04379
254	9521	19951031	12.28	131.90	7.83	35.75	5.21E-05	5.26	2.23E-06	-5.45651	1.75	0.38	0.05362
255	9521	19951101	13.33	128.90	9.50	53.33	9.915E-05	5.28	2.22E-06	-4.66467	1.22	0.27	0.05996
256	9522	19951101	20.53	154.68	8.97	10.50	7.90E-06	8.60	1.40E-06	12.25721	3.14	0.23	0.03636
257	9522	19951102	23.00	151.30	8.33	15.00	5.00E-06	8.48	2.53E-06	6.681265	5.07	0.17	0.04596
258	9523	19951226	10.20	130.40	7.86	15.00	4.81E-05	1.52	1.40E-06	-3.42353	1.53	0.36	0.04089
259	9523	19951227	9.95	129.15	12.97	18.25	0.0001146	3.46	1.40E-06	-1.75826	1.35	0.48	0.05024
260	9523	19951228	10.93	127.83	12.39	23.75	0.0002291	2.76	8.63E-06	-4.2355	1.36	0.49	0.06201
261	9523	19951229	13.25	129.10	10.78	30.00	1.37E-04	5.73	3.74E-06	-10.8915	1.26	0.27	0.0502
262	9523	19951230	17.88	133.78	10.95	27.50	6.08E-05	14.12	-1.15E-06	-22.8126	1.86	0.31	0.0445
263	9523	19951231	21.97	144.00	6.73	16.67	1.98E-05	15.28	2.32E-06	-19.0156	5.01	0.27	0.04821
264	9601	19960401	9.00	145.00	7.57	12.00	2.40E-05	1.27	2.66E-06	-2.20319	1.25	0.17	0.03154
265	9601	19960402	9.23	143.88	8.51	15.00	8.30E-06	2.64	2.20E-06	2.133117	1.51	0.21	0.03457
266	9601	19960403	9.83	141.60	8.38	12.75	1.39E-06	3.86	2.08E-06	4.547046	1.82	0.30	0.03654
267	9601	19960404	8.85	137.83	6.68	14.25	1.44E-05	7.66	2.76E-06	3.522921	1.39	0.19	0.03031
268	9601	19960405	9.78	132.18	10.16	15.75	2.39E-05	6.01	3.73E-06	11.1578	1.52	0.68	0.05059
269	9601	19960406	10.40	128.40	9.56	18.00	2.92E-05	5.67	3.34E-06	21.53065	1.33	0.61	0.04808
270	9602	19960509	8.30	139.70	6.77	12.00	7.17E-05	4.33	5.82E-06	1.151597	1.79	0.42	0.04363
271	9602	19960510	9.48	136.80	4.69	16.50	3.11E-05	7.97	5.17E-06	1.641365	1.36	0.47	0.04128
272	9602	19960511	10.73	131.23	6.23	19.50	2.56E-05	4.83	1.44E-06	-15.5901	1.52	0.29	0.03887
273	9602	19960512	12.95	128.45	8.80	27.00	7.902E-05	3.97	2.23E-06	-18.007	1.25	0.36	0.04865
274	9602	19960513	14.30	127.30	12.22	33.00	0.0001762	3.25	3.50E-06	-20.9012	1.17	0.43	0.06067
275	9602	19960516	20.15	128.15	16.63	37.50	7.50E-05	7.19	5.06E-06	-2.6362	2.67	0.71	0.07581
276	9602	19960517	21.80	130.95	15.66	31.50	8.06E-05	10.15	1.33E-06	-0.1457	3.21	0.45	0.06226
277	9604	19960705	19.93	147.88	5.71	13.50	1.12E-05	2.26	-3.34E-06	-4.42942	3.20	0.35	0.04139
278	9604	19960706	20.78	145.93	6.78	15.75	1.64E-05	4.07	2.52E-06	-3.68156	3.84	0.41	0.05076
279	9604	19960707	21.88	142.85	6.32	22.50	1.83E-05	3.51	6.02E-06	-0.86814	4.66	0.39	0.05959
280	9604	19960708	22.90	141.40	7.40	30.00	2.35E-05	14.50	-5.12E-07	0.094576	5.07	0.47	0.06062
281	9605	19960714	20.73	141.13	7.91	17.00	9.23E-06	4.62	-3.37E-06	0.791044	4.08	0.34	0.04676
282	9605	19960715	22.53	138.00	10.70	29.33	3.07E-05	5.34	1.57E-06	3.778446	3.85	0.33	0.0572
283	9607	19960721	12.23	131.67	3.79	15.00	1.52E-05	4.06	-4.03E-07	-18.3593	1.69	0.24	0.03334
284	9607	19960722	13.75	128.78	7.29	17.25	4.26E-05	5.23	-2.74E-08	-12.1657	1.16	0.32	0.03712
285	9608	19960723	16.85	153.10	8.01	15.00	-1.18E-06	6.10	-1.63E-06	-8.47604	2.51	0.24	0.03652
286	9608	19960724	19.73	149.05	9.02	17.75	1.14E-05	5.80	1.65E-06	-8.73645	2.80	0.44	0.04866
287	9608	19960725	20.48	144.30	8.22	22.00	2.02E-05	6.69	-3.96E-07	-1.46897	4.16	0.41	0.05392
288	9608	19960726	20.60	138.63	8.39	32.75	2.82E-05	6.55	-2.11E-06	-4.49009	3.15	0.34	0.05294
289	9608	19960727	19.23	134.33	7.46	43.75	3.75E-05	4.19	4.38E-06	0.214583	2.78	0.31	0.06082
290	9608	19960728	18.45	131.38	9.42	45.00	8.18E-05	3.10	8.29E-06	-3.00231	2.20	0.42	0.06808
291	9608	19960729	19.48	129.10	11.68	45.00	0.0001336	4.15	2.85E-06	-10.7607	1.77	0.37	0.06467
292	9608	19960730	21.50	127.45	10.26	47.50	9.16E-05	12.70	-1.29E-06	-2.928	3.30	0.54	0.06949
293	9615	19960905	14.30	133.70	4.20	11.75	1.53E-05	5.55	8.83E-07	0.758194	1.94	0.18	0.02952
294	9615	19960906	16.78	129.93	7.78	22.00	2.85E-05	8.54	5.00E-06	2.072716	1.34	0.42	0.0441
295	9616	19960912	16.55	130.03	5.97	14.25	2.95E-05	1.78	1.64E-06	-0.16849	1.46	0.30	0.0365
296	9616	19960913	16.38	129.15	7.13	25.00	4.48E-05	1.55	2.65E-06	-1.86078	1.09	0.23	0.04064
297	9616	19960914	17.00	128.25	9.67	35.00	8.675E-05	2.67	4.62E-06	-4.06311	1.17	0.33	0.05354
298	9616	19960915	18.25	127.15	10.62	42.50	0.0001075	7.89	5.48E-06	-8.97905	1.48	0.40	0.06152
299	9616	19960916	21.00	127.10	14.96	45.00	0.0002301	2.85	3.43E-06	-3.82677	3.17	0.36	0.07605

300	9616	19960917	21.98	128.55	14.94	41.25	0.0002183	3.53	-2.65E-07	-6.32703	3.31	0.24	0.06807
301	9616	19960918	22.90	130.00	14.27	40.00	0.000187	2.83	3.04E-06	-13.037	2.98	0.54	0.07675
302	9617	19960912	17.30	148.00	6.78	12.00	1.26E-05	6.15	-1.18E-06	0.048067	2.57	0.16	0.03168
303	9617	19960913	19.25	146.10	8.60	18.25	1.75E-05	4.87	-1.17E-06	-3.12713	3.39	0.21	0.04233
304	9617	19960914	19.98	143.40	5.56	23.75	1.89E-05	2.13	-6.08E-07	-4.37935	4.09	0.36	0.05347
305	9617	19960915	21.28	144.35	7.15	31.25	3.33E-05	3.14	1.23E-06	-0.85145	4.58	0.41	0.06308
306	9617	19960916	22.37	146.00	8.92	35.00	4.94E-05	4.07	3.44E-06	-1.44581	4.48	0.18	0.05974
307	9619	19960923	16.20	155.48	6.00	23.25	1.63E-05	5.60	3.08E-06	5.067609	2.60	0.21	0.04175
308	9619	19960924	16.18	150.78	4.54	35.00	1.01E-05	6.08	5.54E-07	-13.8541	2.34	0.20	0.04572
309	9619	19960925	16.08	146.13	5.22	40.00	1.34E-05	4.92	1.09E-06	-14.5207	2.37	0.20	0.04991
310	9619	19960926	17.18	142.75	7.25	40.00	1.79E-05	4.67	-2.32E-07	-12.0017	2.34	0.34	0.05502
311	9619	19960927	19.83	139.93	7.10	40.00	2.46E-05	4.45	-2.17E-07	-8.42147	3.39	0.22	0.05573
312	9619	19960928	21.83	137.93	8.15	40.00	3.57E-05	3.28	1.78E-06	-7.59492	3.39	0.16	0.05624
313	9620	19960924	15.53	136.83	11.01	15.00	2.62E-05	7.69	3.74E-06	6.326763	1.86	0.34	0.04107
314	9620	19960925	17.18	131.80	9.34	22.25	2.05E-05	6.32	-4.59E-06	17.28535	2.28	0.34	0.0407
315	9620	19960926	19.70	128.23	9.34	32.75	2.91E-05	4.50	-1.01E-06	-1.17491	2.21	0.28	0.04892
316	9621	19961013	15.55	133.55	3.79	12.00	9.83E-06	4.73	-1.17E-06	9.364573	1.92	0.26	0.03047
317	9621	19961014	16.08	131.05	3.81	15.00	1.08E-05	3.35	5.88E-06	-0.22094	2.28	0.48	0.04604
318	9621	19961015	17.25	129.18	4.69	15.00	1.69E-05	3.24	-3.78E-06	-1.21464	1.10	0.42	0.03426
319	9621	19961016	17.50	127.60	6.25	18.00	1.81E-05	3.48	4.86E-07	-0.17879	1.44	0.67	0.0487
320	9622	19961021	19.08	148.85	9.22	14.25	2.60E-05	2.91	1.36E-06	-5.48459	2.61	0.21	0.03978
321	9622	19961022	19.40	147.08	7.62	19.75	1.94E-05	2.51	-2.50E-06	7.048928	3.15	0.22	0.04157
322	9622	19961023	20.63	145.03	7.09	28.75	3.03E-05	3.85	7.62E-06	8.580417	4.00	0.47	0.0638
323	9622	19961024	22.60	144.60	10.02	35.00	3.78E-05	5.15	1.04E-06	11.15727	5.17	0.36	0.06635
324	9623	19961104	10.20	152.20	9.19	12.00	3.14E-05	0.51	-1.74E-08	2.256039	0.97	0.14	0.02903
325	9623	19961105	10.45	151.98	11.30	15.75	6.32E-05	1.21	9.60E-07	-2.69103	1.04	0.23	0.0372
326	9623	19961106	11.38	150.18	11.81	20.75	0.0001123	4.97	5.25E-06	-3.80528	1.55	0.35	0.04941
327	9623	19961107	11.50	146.53	15.23	30.00	0.0003136	5.63	4.76E-06	-0.43692	1.06	0.49	0.06492
328	9623	19961108	11.88	141.75	18.02	36.25	0.0005416	6.10	6.86E-06	-5.09888	1.71	0.46	0.08112
329	9623	19961109	14.15	137.08	17.95	48.75	0.0005665	8.35	4.49E-06	-0.03391	1.88	0.54	0.08969
330	9623	19961110	18.08	132.55	18.32	50.00	0.0004391	5.96	5.11E-06	4.71315	1.86	0.37	0.08183
331	9623	19961111	21.70	131.27	15.60	48.33	0.0002863	5.79	4.65E-06	-4.5285	3.13	0.59	0.08717
332	9625	19961223	9.55	140.23	9.71	15.00	5.19E-05	2.27	3.01E-06	3.488212	1.87	0.28	0.04127
333	9625	19961224	9.20	138.78	8.46	19.50	7.57E-05	2.53	3.08E-06	4.762819	1.70	0.33	0.04467
334	9625	19961225	9.60	137.90	11.23	25.00	0.0001993	2.20	3.44E-06	5.919264	1.66	0.33	0.05365
335	9625	19961226	11.35	138.50	10.84	26.25	0.0001966	2.84	6.10E-06	5.909147	1.81	0.35	0.05649
336	9625	19961227	13.53	139.00	9.79	30.00	0.0001208	2.72	2.38E-06	-11.4348	1.99	0.27	0.05275
337	9625	19961228	15.75	140.58	10.34	27.50	0.0001186	6.29	2.33E-06	-17.5166	2.03	0.20	0.04854
338	9625	19961229	18.63	145.65	9.51	21.25	3.44E-05	8.62	-3.99E-06	-11.2346	3.18	0.20	0.04123
339	9625	19961230	19.80	150.90	7.28	13.50	1.87E-05	8.97	3.72E-06	-12.7566	3.41	0.24	0.04184
340	9701	19970412	7.48	157.90	7.88	13.00	7.95E-05	2.25	2.17E-06	-2.87449	0.91	0.33	0.03746
341	9701	19970413	8.68	156.85	9.48	17.25	8.93E-05	3.43	3.96E-06	1.986087	1.15	0.37	0.04345
342	9701	19970414	10.08	153.75	10.02	21.50	7.46E-05	6.19	2.16E-06	14.87345	1.23	0.42	0.04478
343	9701	19970415	10.15	148.83	6.85	26.25	3.27E-05	4.96	2.31E-06	6.648638	0.79	0.34	0.04075
344	9701	19970416	10.73	145.50	11.05	33.00	0.0001414	4.34	2.94E-06	2.004049	1.18	0.31	0.05251
345	9701	19970417	12.15	142.38	12.37	38.75	0.0001771	4.30	3.60E-06	0.967744	1.64	0.57	0.06849
346	9701	19970418	13.43	139.63	14.27	40.00	0.0002642	2.94	3.81E-06	-4.87581	2.00	0.40	0.07051
347	9701	19970419	15.10	138.05	15.59	46.25	0.0002852	3.30	4.28E-06	-3.13053	2.13	0.42	0.07683
348	9701	19970420	17.85	137.55	16.86	50.00	0.0002257	4.37	3.50E-06	3.585925	2.46	0.46	0.07888
349	9701	19970421	21.00	138.10	19.52	40.00	1.39E-04	6.34	2.89E-06	2.53914	3.09	0.49	0.07451
350	9703	19970527	11.70	156.40	8.04	10.00	2.04E-05	3.59	-3.76E-07	-8.43021	2.17	0.30	0.03661
351	9703	19970528	12.83	157.70	7.17	10.50	3.37E-05	3.56	-8.19E-10	-2.68362	2.64	0.16	0.03397
352	9703	19970529	15.30	159.18	8.74	17.00	3.57E-05	4.54	1.56E-06	3.620894	2.92	0.23	0.04234
353	9703	19970530	18.50	159.87	5.46	22.67	1.95E-05	5.13	4.22E-07	5.968912	2.71	0.18	0.03932
354	9704	19970607	12.10	150.67	4.49	12.33	7.50E-06	3.67	1.24E-06	-7.93958	1.99	0.37	0.03778
355	9704	19970608	14.10	149.90	5.45	20.75	1.19E-05	2.59	-2.89E-06	-4.48929	2.25	0.25	0.03814
356	9704	19970609	15.85	149.18	8.62	38.75	2.60E-05	2.82	7.77E-07	-1.13385	2.26	0.29	0.05389
357	9704	19970610	18.03	147.75	9.40	48.75	3.66E-05	4.25	-4.56E-07	-1.08951	2.66	0.20	0.05788
358	9704	19970611	20.38	144.85	6.81	46.25	3.13E-05	5.03	-1.77E-06	-6.51353	3.96	0.17	0.05877
359	9704	19970612	22.50	142.50	8.07	45.00	3.55E-05	6.39	5.45E-07	0.563494	4.86	0.41	0.07099
360	9705	19970614	14.15	133.65	4.78	10.00	2.82E-06	1.62	-1.75E-06	-2.97979	1.96	0.31	0.03305
361	9705	19970615	14.83	133.20	3.84	13.50	5.70E-06	1.20	-3.10E-06	-5.57652	2.01	0.24	0.03225
362	9705	19970616	15.45	132.50	4.80	22.00	1.69E-05	1.79	1.10E-06	-5.38601	2.39	0.33	0.04465
363	9705	19970617	17.90	132.68	9.61	33.25	4.88E-05	5.75	2.94E-07	5.019129	1.84	0.34	0.0502
364	9705	19970618	21.50	133.65	12.14	40.00	5.99E-05	8.22	-2.40E-07	4.311757	3.33	0.60	0.06925
365	9706	19970622	13.23	132.23	3.82	12.00	1.28E-05	0.91	6.90E-07	-8.74149	1.92	0.22	0.03309
366	9706	19970623	13.65	131.13	5.39	14.25	2.59E-05	2.73	1.52E-06	-15.8879	1.88	0.26	0.03702
367	9706	19970624	15.73	128.73	8.45	15.00	3.06E-05	5.27	-1.34E-06	-11.8884	1.16	0.23	0.03215
368	9706	19970625	18.23	127.13	9.39	20.33	3.59E-05	2.98	-9.33E-08	-5.90501	1.48	0.43	0.04509
369	9707	19970718	10.25	138.25	4.06	11.00	2.25E-05	2.12	2.92E-06	-6.83072	1.86	0.30	0.03602
370	9707	19970719	11.95	137.53	6.85	14.25	4.69E-05	3.07	1.30E-06	-7.38378	1.88	0.19	0.03561
371	9707	19970720	14.10	136.18	8.71	22.75	5.31E-05	3.69	1.21E-06	-12.6421	1.87	0.13	0.03977
372	9707	19970721	16.15	133.90	10.45	32.50	0.0001145	4.02	5.27E-06	-14.8402	1.79	0.29	0.05541
373	9707	19970722	18.03	132.20	12.66	47.50	0.000169	2.28	5.23E-06	-7.91441	1.92	0.32	0.06899
374	9707	19970723	20.23	131.95	13.88	53.75	0.0001947	4.30	3.66E-06	2.50877	2.26	0.37	0.07458
375	9707	19970724	22.40	132.40	13.03	50.00	0.0001524	5.32	2.92E-07	1.325118	3.31	0.59	0.08007
376	9709	19970731	13.48	135.50	8.77	17.75	6.95E-05	1.89	2.05E-06	3.178757	1.89	0.32	0.04383
377	9709	19970801	13.63	134.58	8.20	22.75	5.93E-05	2.31	2.02E-06	-6.81766	1.94	0.27	0.04501
378	9709	19970802	14.08	132.40	9.69	30.00	5.81E-05	2.41	2.13E-06	1.145239	2.06	0.35	0.0527
379	9709	19970803	15.33	130.68	9.57	30.00	6.72E-05	4.53	3.40E-06	-13.2726	2.02	0.17	0.04557
380	9709	19970804	18.30	128.30	7.95	33.00	4.30E-05	4.71	2.69E-08	-23.9719	1.34	0.48	0.05344
381	9709	19970805	20.10	127.00	8.56	35.00	5.70E-05	5.21	-2.51E-06	-16.1252	2.50	0.45	0.05726
382	9711	19970808	12.33	157.75	3.80	10.50	9.95E-06	5.58	2.01E-08	-9.3702	2.51	0.16	0.03027
383	9711	19970809	14.65	155.13	5.08	16.25	1.85E-05	2.85	2.27E-07	-10.5358	2.70	0.21	0.03835
384	9711	19970810	15.83	152.35	5.23	27.75	2.13E-05	4.75	6.86E-07	-10.3771	2.46	0.15	0.04151
385	9711	19970811	16.55	149.05	6.84	38.25	3.62E-05	3.83	1.48E-06	-10.2038	2.34	0.36	0.05623
386	9711	19970812	17.90	145.48	5.72	56.25	2.84E-05	5.29	2.17E-06	-3.64871	2.93	0.32	0.06617
387	9711	19970813	19.78	141.78	6.06	58.75	2.66E-05	5.39	-2.09E-07	-1.00059	3.95	0.21	0.06673
388	9711	19970814	21.78	138.15	8.80	47.50	4.91E-05	5.16	3.14E-06	-4.11814	3.50	0.34	0.06756
389	9711	19970815	22.85	135.35	8.84	45.00	5.20E-05	3.66	9.95E-08	-8.63764	4.31	0.29	0.06705
390	9714	19970821	13.68	131.55	9.33	14.25	3.32E-05	1.84	2.07E-06	-2.77104	1.96	0.27	0.04008
391	9714	19970822	15.05	131.63	7.55	22.50	5.10E-05	1.73	2.61E-07	-6.10079	2.39	0.22	0.0437
392	9714	19970823	16.20	131.00	7.96	31.50	5.94E-05	2.05	3.42E-06	-11.1604	2.20	0.29	0.05304
393	9714	19970824	17.10	129.55	8.68	40.00	6.44E-05	2.15	2.80E-06	-10.3082	1.18	0.26	0.05245
394	9714	19970825	17.60	128.13	9.39	43.75	8.40E-05	1.58	6.77E-06	-4.0641	1.28	0.45	0.06445
395	9714	19970826	18.10	127.35	10.26	40.00	0.0001006	1.79	6.13E-06	-8.10833	1.47	0.46	0.06416
396	9715	19970827	12.30	152.20	3.08	12.00	6.40E-06	5.66	-1.27E-06	-16.4191	1.99	0.26	0.03135
397	9715	19970828	12.45	147.95	4.22	13.50	1.22E-05	5.36	2.70E-07	-15.3226	1.70	0.14	0.02884
398	9715	19970829	14.30	143.28	4.40	15.75	1.88E-05	7.55	6.98E-07	-17.6255	1.83	0.11	0.02947
399	9715	19970830	16.28	138.88	6.24	23.25	3.14E-05	3.52	-3.30E-07	2.100267	2.43	0.19	0.04009

400	9715	19970831	19.33	138.60	7.70	30.25	4.62E-05	6.38	7.05E-07	8.154165	2.88	0.43	0.05434
401	9715	19970901	22.50	139.20	11.82	40.00	9.06E-05	6.73	-2.12E-06	4.486984	4.39	0.14	0.05921
402	9716	19970908	18.10	158.70	7.87	30.00	1.26E-05	5.90	-2.17E-06	-4.03629	2.86	0.24	0.04641
403	9716	19970909	18.25	155.63	9.87	41.25	2.12E-05	6.55	3.55E-06	-0.58239	2.54	0.24	0.05547
404	9716	19970910	18.95	150.18	9.64	55.00	4.53E-05	6.64	1.29E-06	2.232991	2.90	0.23	0.06356
405	9716	19970911	20.03	144.78	7.51	57.50	3.26E-05	6.79	-4.58E-07	12.20105	3.78	0.33	0.06877
406	9716	19970912	21.77	140.13	7.46	55.00	3.22E-05	7.16	1.67E-06	10.11005	4.22	0.47	0.07515
407	9720	19971013	11.50	150.00	7.46	12.00	1.22E-05	7.64	6.62E-07	2.418043	1.61	0.29	0.03239
408	9720	19971014	12.10	146.00	5.95	17.75	1.30E-05	8.10	-4.65E-07	-0.06282	1.41	0.33	0.03466
409	9720	19971015	13.63	140.53	7.31	23.25	1.40E-05	6.36	1.07E-06	4.29299	1.97	0.33	0.0425
410	9720	19971016	14.55	135.35	7.65	36.25	3.01E-05	7.00	5.50E-07	11.11647	1.83	0.40	0.05113
411	9720	19971017	14.60	130.08	7.80	50.00	2.98E-05	5.77	1.98E-06	11.21953	1.73	0.29	0.05641
412	9720	19971018	14.80	127.20	8.68	55.00	4.70E-05	6.00	7.07E-07	6.370739	1.14	0.28	0.05692
413	9720	19971022	22.35	127.70	7.41	23.00	3.06E-05	5.99	-8.86E-07	-5.4483	3.32	0.19	0.04367
414	9721	19971015	13.40	159.00	8.11	25.50	2.10E-05	5.53	-4.13E-07	-4.23546	2.74	0.23	0.04449
415	9721	19971016	13.75	155.25	8.28	38.25	4.17E-05	6.97	-1.06E-07	-1.18387	2.54	0.35	0.05485
416	9721	19971017	14.78	149.45	6.05	50.00	3.45E-05	7.89	1.34E-06	-1.88277	2.16	0.45	0.06221
417	9721	19971018	16.75	143.83	7.23	55.00	4.33E-05	6.67	1.52E-06	-2.47683	2.22	0.17	0.05774
418	9721	19971019	18.75	139.40	8.09	55.00	5.57E-05	4.93	1.88E-06	-5.5414	3.11	0.26	0.06667
419	9721	19971020	21.18	136.95	9.63	53.75	6.81E-05	3.88	3.86E-06	1.075301	3.39	0.29	0.07057
420	9722	19971029	6.00	160.00	6.87	16.50	1.96E-05	1.81	3.00E-06	-6.50386	0.56	0.28	0.03457
421	9722	19971030	7.08	159.28	11.52	23.00	8.052E-05	3.85	2.07E-06	-1.41678	0.82	0.27	0.04213
422	9722	19971031	9.60	156.20	12.89	37.00	0.0002671	6.64	7.48E-06	0.373534	1.32	0.36	0.06411
423	9722	19971101	12.33	151.00	15.34	51.25	0.0002433	8.25	4.88E-06	-0.20931	2.06	0.57	0.08063
424	9722	19971102	14.55	145.13	15.39	60.00	0.0001987	6.76	3.50E-06	-12.9207	1.84	0.54	0.08301
425	9722	19971103	15.68	140.05	14.58	53.75	0.0002316	6.03	3.28E-06	-13.518	2.02	0.43	0.07755
426	9722	19971104	16.70	136.13	14.41	45.00	0.000257	3.76	4.51E-06	-11.0283	1.58	0.44	0.07314
427	9722	19971105	18.63	135.18	14.25	41.25	0.0002329	3.63	5.06E-06	-6.92203	2.12	0.44	0.07269
428	9722	19971106	21.60	137.28	15.27	28.75	0.0001821	5.84	6.62E-07	5.979191	3.38	0.34	0.06227
429	9724	19971111	12.00	137.63	6.33	16.67	1.82E-05	5.49	-4.36E-07	-0.49135	1.87	0.38	0.03911
430	9724	19971112	13.15	134.20	8.66	22.25	2.66E-05	4.04	1.17E-06	5.017805	1.97	0.33	0.04393
431	9724	19971113	13.83	131.13	7.88	23.00	3.51E-05	4.62	3.22E-06	-3.52632	1.93	0.22	0.04187
432	9724	19971114	13.80	127.97	6.60	22.00	3.52E-05	4.95	7.43E-08	3.45638	1.07	0.24	0.03511
433	9725	19971213	10.00	159.30	8.51	40.00	7.59E-05	9.48	2.17E-06	4.023611	2.12	0.48	0.0594
434	9725	19971214	11.23	155.05	10.76	43.75	0.0001275	8.44	1.04E-06	9.259136	1.90	0.50	0.06371
435	9725	19971215	12.88	149.13	12.16	51.25	0.0001582	6.26	1.82E-06	6.834875	2.14	0.47	0.07173
436	9725	19971216	13.78	145.13	11.83	50.00	0.000162	3.89	4.42E-06	-3.17543	1.75	0.39	0.06966
437	9725	19971217	13.85	141.88	12.04	46.25	0.0001728	4.38	3.89E-06	1.403269	1.84	0.41	0.06828
438	9725	19971218	14.78	138.58	13.30	50.00	7.95E-05	4.34	2.87E-06	8.022765	2.08	0.42	0.06828
439	9725	19971219	15.88	135.43	13.18	42.50	4.44E-05	3.98	1.12E-06	11.82168	1.55	0.43	0.05961
440	9725	19971220	17.45	133.28	9.26	33.75	1.44E-06	2.48	-6.40E-07	32.0606	1.93	0.33	0.04754
441	9725	19971221	18.10	132.15	7.49	22.00	1.46E-05	1.87	-6.81E-07	7.112773	1.93	0.36	0.0432
442	9725	19971222	18.50	131.30	6.40	15.00	8.60E-06	2.11	-3.63E-07	-21.4324	2.23	0.24	0.03797
443	9805	19980912	19.23	143.93	7.13	15.00	1.04E-05	4.98	-1.59E-06	-16.0715	3.37	0.15	0.03808
444	9805	19980913	21.80	141.70	9.10	16.50	1.65E-05	3.67	-1.27E-06	-7.76977	4.46	0.38	0.05253
445	9806	19980915	20.55	129.05	4.13	12.00	1.14E-05	5.66	1.11E-06	-11.5833	2.74	0.31	0.03804
446	9806	19980916	21.18	132.15	5.11	22.00	1.69E-05	4.55	1.69E-06	2.045429	2.52	0.34	0.04448
447	9806	19980917	22.20	134.20	7.84	35.00	4.67E-05	5.29	3.31E-06	-0.18968	3.89	0.27	0.05896
448	9809	19981010	10.48	140.18	6.72	15.75	2.85E-05	5.23	-1.94E-07	-2.64065	1.83	0.18	0.03307
449	9809	19981011	10.88	135.58	7.30	24.00	4.33E-05	6.72	2.10E-06	-0.84986	1.65	0.33	0.04322
450	9809	19981012	12.48	130.83	6.99	35.00	5.28E-05	7.58	-7.76E-07	-0.72879	1.61	0.16	0.04171
451	9809	19981013	14.70	127.80	11.47	50.00	0.0001006	8.27	-1.15E-06	6.745598	1.08	0.21	0.05285
452	9810	19981017	11.35	131.18	6.92	15.00	1.89E-05	2.68	-1.17E-06	-19.8209	1.49	0.17	0.03216
453	9810	19981018	11.00	129.95	6.69	16.25	1.29E-05	0.99	3.95E-06	-4.30642	1.41	0.37	0.04148
454	9810	19981019	10.65	129.48	10.41	28.25	5.72E-05	2.34	6.92E-06	-6.23775	1.40	0.56	0.05942
455	9810	19981020	11.83	127.70	9.76	45.00	6.974E-05	3.15	9.05E-08	-15.4566	1.34	0.22	0.0537
456	9901	19990423	14.25	127.50	8.39	16.50	4.21E-05	3.99	-9.68E-07	-2.8121	1.13	0.20	0.03237
457	9901	19990424	15.28	129.60	7.03	22.25	2.94E-05	3.58	-1.70E-07	0.977016	1.43	0.15	0.0347
458	9901	19990425	17.90	131.60	6.49	25.25	2.14E-05	5.16	-3.53E-08	-3.89959	2.08	0.30	0.0431
459	9901	19990426	20.93	134.50	6.32	28.75	2.19E-05	7.09	-1.02E-06	-5.20798	3.67	0.21	0.04758
460	9903	19990601	12.40	129.80	9.13	13.00	1.70E-05	2.42	-8.76E-07	-13.3373	1.49	0.16	0.03179
461	9903	19990602	13.68	129.75	10.04	20.25	2.00E-05	1.99	1.52E-06	-13.3676	1.46	0.16	0.03788
462	9903	19990603	15.65	128.73	6.46	27.75	1.73E-05	4.43	9.74E-07	-20.5298	1.16	0.18	0.03866
463	9903	19990604	17.10	127.00	7.06	33.00	4.87E-05	8.99	2.69E-06	-16.2743	1.52	0.39	0.05028
464	9906	19990729	14.03	133.88	9.25	15.00	5.45E-05	5.90	3.63E-06	-7.88766	1.97	0.20	0.03847
465	9906	19990730	19.20	132.90	8.52	20.25	1.72E-05	7.41	3.31E-06	17.69915	2.30	0.34	0.04306
466	9906	19990731	22.20	130.95	8.23	26.50	3.79E-05	5.65	2.79E-06	11.80658	3.35	0.35	0.05286
467	9907	19990803	20.30	139.35	11.38	13.50	3.93E-05	5.64	1.97E-06	-15.2667	3.25	0.22	0.04406
468	9907	19990804	22.50	137.50	8.79	18.00	2.83E-05	6.40	9.08E-08	-4.892	3.70	0.24	0.04525
469	9912	19990917	20.00	131.00	6.05	12.00	2.93E-05	1.78	2.03E-06	-11.493	2.48	0.25	0.03885
470	9912	19990918	20.68	130.25	6.28	13.50	2.57E-05	3.98	1.92E-06	-8.95439	2.77	0.24	0.03944
471	9912	19990919	22.10	128.20	6.16	15.00	2.80E-05	7.60	3.86E-06	-8.35312	3.34	0.21	0.04147
472	9914	19991002	17.00	131.50	4.84	12.00	9.34E-06	6.44	1.17E-06	-7.1279	2.28	0.25	0.03381
473	9914	19991003	18.05	129.05	7.89	17.50	3.22E-05	8.28	-1.33E-06	1.167492	1.23	0.22	0.03136
474	9916	19991113	16.70	131.20	4.63	12.00	1.42E-05	7.10	8.52E-07	5.716658	2.19	0.28	0.03309
475	9916	19991114	19.50	130.48	4.97	19.50	1.88E-05	5.85	1.55E-06	-8.35088	2.35	0.15	0.036
476	9916	19991115	22.30	131.30	4.67	25.00	1.54E-05	7.69	-2.32E-07	-22.4939	3.40	0.19	0.044
477	1	20000505	10.10	134.78	5.87	15.00	1.92E-05	4.30	9.31E-07	-3.06801	1.62	0.50	0.04239
478	1	20000506	12.10	132.30	6.40	15.00	1.85E-05	3.29	-7.79E-07	-14.1283	1.80	0.23	0.03461
479	1	20000507	13.53	131.35	8.44	25.75	4.32E-05	1.40	-4.77E-07	-13.0996	1.91	0.16	0.04202
480	1	20000508	14.50	132.23	10.39	35.75	6.73E-05	3.24	1.08E-06	-4.9028	2.17	0.19	0.05134
481	1	20000509	17.00	134.80	11.41	48.75	6.20E-05	6.52	1.07E-06	5.814794	1.47	0.36	0.05949
482	1	20000510	21.38	138.23	14.55	43.75	8.516E-05	6.86	7.93E-06	-0.81144	3.25	0.42	0.07331
483	3	20000702	14.20	132.70	7.48	15.00	3.87E-05	4.33	9.73E-07	-11.2034	2.04	0.15	0.035
484	3	20000703	16.33	131.83	7.82	23.75	4.78E-05	2.70	5.63E-07	-14.0862	2.60	0.31	0.04854
485	3	20000704	18.68	131.60	11.88	38.25	0.0001276	4.55	2.02E-06	-20.9693	2.22	0.30	0.06045
486	3	20000705	21.57	132.50	13.97	45.00	0.0001252	4.92	4.95E-06	0.263731	2.77	0.63	0.07863
487	8	20000801	22.07	151.13	7.10	18.67	1.74E-05	4.16	2.46E-07	-2.6097	4.93	0.19	0.04885
488	8	20000802	22.33	148.57	8.97	36.00	2.39E-05	4.36	-3.80E-06	7.57267	4.17	0.47	0.06276
489	9	20000809	14.43	140.98	8.95	14.25	3.42E-05	6.57	2.16E-06	-17.4981	1.89	0.17	0.03552
490	9	20000810	16.93	137.98	8.29	18.50	3.34E-05	8.62	1.27E-06	-7.49055	2.39	0.24	0.03982
491	10	20000818	12.10	137.15	7.62	15.00	4.11E-05	6.77	-2.61E-07	-15.2465	1.86	0.21	0.03471
492	10	20000819	15.03	134.95	8.85	22.00	5.75E-05	6.53	1.13E-06	-0.13299	1.74	0.29	0.0419
493	10	20000820	17.28	131.18	7.52	35.25	5.08E-05	5.97	3.02E-06	-1.03238	2.02	0.20	0.04813
494	10	20000821	19.10	127.70	9.01	52.50	6.51E-05	6.61	1.57E-06	-2.74746	1.70	0.32	0.06095
495	14	20000902	16.00	156.00	3.51	12.00	1.22E-05	5.84	1.01E-06	-12.1605	2.70	0.13	0.03173
496	14	20000903	15.78	153.83	4.01	20.75	1.25E-05	4.23	-1.12E-06	-10.6415	2.42	0.14	0.03522
497	14	20000904	16.08	150.38	5.28	31.50	1.90E-05	4.51	-6.95E-07	-7.16523	2.31	0.22	0.04432
498	14	20000905	15.33	147.53	5.58	30.75	2.26E-05	4.29	1.65E-07	-3.75945	2.09	0.20	0.04306
499	14	20000906	14.10	147.33	7.30	27.50	2.82E-05	3.41	7.96E-08	-7.9976	1.85	0.18	0.04122

500	14	20000907	16.85	144.18	7.07	25.00	3.04E-05	7.02	-5.50E-07	-20.3549	2.31	0.10	0.03816
501	14	20000908	19.33	139.93	8.93	27.50	5.214E-05	5.84	-5.52E-07	-17.4146	3.43	0.30	0.05316
502	14	20000909	21.60	136.77	8.44	37.67	3.79E-05	6.84	6.96E-07	-9.22735	3.59	0.13	0.0529
503	19	20001022	21.58	136.45	8.95	17.75	1.87E-05	8.04	3.46E-07	9.484316	3.75	0.33	0.04648
504	19	20001023	22.58	130.80	6.49	22.50	1.81E-05	5.25	1.62E-06	5.614134	3.33	0.24	0.04515
505	19	20001024	23.00	128.20	8.39	25.00	2.78E-05	4.86	-1.35E-06	12.04896	3.33	0.23	0.04568
506	20	20001026	10.68	130.85	5.11	17.75	3.12E-05	7.39	4.56E-07	-9.27583	1.50	0.35	0.03725
507	20	20001027	12.00	128.20	13.35	23.00	0.0002068	3.39	1.84E-06	1.0918	1.39	0.38	0.0532
508	21	20001031	9.57	130.87	6.85	15.00	1.10E-05	5.29	1.43E-06	11.03514	1.51	0.39	0.03729
509	21	20001101	11.27	128.10	9.14	18.67	5.25E-05	7.01	1.46E-06	-4.27312	1.39	0.22	0.03664
510	22	20001128	8.75	131.00	9.85	18.50	8.074E-05	3.38	3.49E-06	7.205543	1.40	0.35	0.04413
511	22	20001129	8.85	129.95	6.79	23.00	6.67E-05	3.27	3.30E-06	10.6685	1.24	0.40	0.04491
512	22	20001130	9.25	127.80	5.93	23.00	4.49E-05	11.86	3.50E-06	7.07063	1.13	0.23	0.03429
513	23	20001229	8.83	129.43	5.23	13.50	4.37E-05	5.23	1.64E-06	-0.70495	1.09	0.38	0.03561
514	23	20001230	11.03	127.38	8.73	20.25	7.395E-05	2.54	2.91E-06	-13.1113	1.31	0.22	0.04092
515	23	20001231	13.18	128.85	9.59	24.00	5.08E-05	6.11	7.44E-07	-10.4266	1.24	0.15	0.0374
516	23	20010101	15.33	132.73	10.71	24.00	8.18E-05	3.82	7.75E-07	1.772034	2.27	0.25	0.04689
517	23	20010102	16.43	134.65	6.55	23.00	3.42E-05	1.89	-6.68E-07	-1.90847	1.56	0.22	0.03845
518	23	20010103	17.70	135.65	7.70	37.00	4.40E-05	2.49	-1.89E-06	-22.3352	1.51	0.20	0.04676
519	23	20010104	18.33	137.13	13.49	26.50	4.70E-05	1.86	2.83E-06	-17.6112	2.42	0.24	0.05248
520	23	20010105	18.00	138.00	15.82	15.00	1.42E-05	2.21	6.20E-07	-11.1659	2.57	0.25	0.0452
521	102	20010619	11.90	136.40	8.93	12.00	2.27E-05	7.63	-2.07E-06	-7.44445	1.87	0.22	0.03161
522	102	20010620	13.25	132.15	5.81	18.25	2.01E-05	9.43	-1.03E-07	-1.31433	1.92	0.21	0.03297
523	102	20010621	14.70	127.45	4.97	23.00	1.59E-05	5.38	1.39E-06	-0.64825	1.12	0.18	0.03331
524	104	20010701	7.93	138.13	9.69	15.00	0.0001633	6.21	2.25E-06	-12.675	1.43	0.32	0.04332
525	104	20010702	11.98	135.45	10.52	21.50	0.0001015	8.21	-2.09E-08	-9.43266	1.83	0.15	0.03936
526	104	20010703	15.20	129.73	14.96	30.33	0.0002919	13.78	3.50E-06	-8.94256	1.51	0.32	0.05728
527	108	20010725	14.33	132.93	5.34	12.00	1.35E-05	4.96	-3.60E-07	-19.9252	2.02	0.18	0.03161
528	108	20010726	16.55	130.23	4.72	15.00	1.60E-05	5.60	1.58E-07	-14.5975	1.56	0.22	0.03184
529	108	20010727	16.90	127.40	6.90	20.00	4.21E-05	5.43	-1.73E-06	-8.7142	1.36	0.15	0.03239
530	109	20010801	10.40	152.80	5.38	12.00	1.59E-05	5.76	-7.29E-07	-1.81601	1.12	0.18	0.02584
531	109	20010802	12.20	150.75	7.70	19.00	2.11E-05	6.42	2.96E-07	4.920601	2.03	0.18	0.03519
532	109	20010803	16.70	147.23	9.72	27.75	3.31E-05	7.56	-4.92E-07	1.749251	2.54	0.22	0.04447
533	109	20010804	21.10	144.60	8.34	40.00	1.85E-05	7.09	-6.45E-06	0.090072	4.43	0.38	0.06042
534	111	20010814	18.90	145.65	12.71	16.25	5.33E-05	4.55	5.29E-06	-1.09861	3.30	0.34	0.05245
535	111	20010815	20.05	141.88	7.72	25.00	4.16E-05	4.03	2.93E-06	-5.52115	4.08	0.38	0.05808
536	111	20010816	21.13	139.35	6.18	32.50	2.74E-05	2.72	-2.80E-07	-2.35273	3.62	0.27	0.05382
537	111	20010817	21.90	137.48	7.04	35.00	3.44E-05	2.39	2.81E-06	-0.18836	3.45	0.15	0.05325
538	111	20010818	22.80	136.80	7.44	35.00	3.10E-05	2.73	-2.13E-07	4.725866	3.82	0.13	0.05191
539	112	20010826	16.30	140.10	9.76	15.00	4.16E-05	3.34	-7.94E-07	-18.2478	2.28	0.15	0.03721
540	112	20010827	17.73	141.30	8.88	17.00	5.22E-05	4.85	1.78E-06	2.358827	2.97	0.31	0.04577
541	112	20010828	20.85	143.95	8.74	31.25	5.42E-05	5.04	-1.69E-06	8.101446	4.65	0.31	0.05873
542	112	20010829	22.80	145.40	6.62	40.00	2.97E-05	4.31	3.51E-06	0.190741	4.79	0.31	0.06615
543	115	20010903	18.83	155.28	6.14	13.50	1.48E-05	4.26	-5.23E-07	-15.8627	2.54	0.32	0.03963
544	115	20010904	18.78	152.00	6.24	22.50	3.41E-05	3.17	-1.17E-06	-13.6003	3.20	0.15	0.04261
545	115	20010905	21.10	150.90	7.58	36.25	3.13E-05	4.06	5.75E-07	-5.08721	4.31	0.25	0.0595
546	115	20010906	23.00	150.50	10.34	40.00	4.75E-05	3.62	4.31E-06	-0.75954	4.62	0.27	0.06755
547	118	20010920	16.48	158.08	9.23	19.75	1.18E-05	7.78	1.45E-06	4.285308	2.98	0.19	0.04088
548	118	20010921	19.10	152.40	9.62	25.75	2.57E-05	6.45	4.21E-07	-0.12054	3.24	0.13	0.0444
549	118	20010922	21.60	149.33	7.53	31.67	2.66E-05	5.29	1.10E-06	10.18449	3.81	0.22	0.05225
550	120	20011003	14.55	145.75	7.53	12.00	1.97E-05	7.28	4.09E-07	-1.08505	1.89	0.32	0.03513
551	120	20011004	16.15	142.10	7.72	21.00	3.69E-05	6.56	1.94E-06	8.065682	2.19	0.18	0.03822
552	120	20011005	19.30	137.83	9.04	35.75	2.98E-05	6.30	3.23E-06	10.23642	2.76	0.23	0.05214
553	120	20011006	21.90	135.50	10.14	40.00	2.36E-05	5.88	2.31E-06	7.935067	4.05	0.26	0.06134
554	121	20011012	17.33	129.95	5.41	15.00	2.29E-05	1.48	-2.14E-06	-2.77819	1.44	0.20	0.0309
555	121	20011013	18.48	129.83	6.15	21.50	2.57E-05	1.69	7.78E-07	-4.41803	1.72	0.22	0.03868
556	121	20011014	20.60	129.35	6.19	29.00	2.94E-05	4.99	3.10E-06	1.490692	2.68	0.20	0.04631
557	121	20011015	22.30	127.60	7.74	33.00	4.03E-05	5.42	3.28E-06	-3.62911	3.34	0.22	0.05355
558	122	20011018	5.43	156.38	7.78	12.00	5.74E-05	0.93	1.89E-07	5.35372	0.24	0.41	0.03438
559	122	20011019	5.70	156.15	5.55	13.50	5.92E-05	1.96	1.68E-06	-5.01807	0.32	0.38	0.03461
560	122	20011020	8.35	156.50	6.01	20.25	5.53E-05	5.11	2.89E-06	0.920896	0.97	0.22	0.03525
561	122	20011021	11.98	156.50	9.98	29.00	4.67E-05	3.45	8.61E-07	3.272347	2.26	0.38	0.05241
562	122	20011022	14.23	156.85	10.54	35.75	5.68E-05	2.55	1.97E-07	-4.11603	2.79	0.15	0.05196
563	122	20011023	15.75	157.73	9.34	45.00	5.51E-05	2.60	9.31E-07	-12.7644	2.97	0.20	0.06015
564	122	20011024	17.30	156.58	12.51	53.75	0.0001224	3.48	2.78E-06	-12.3256	2.76	0.28	0.07138
565	122	20011025	19.68	154.43	10.24	56.25	1.20E-04	4.72	-9.49E-07	7.87812	3.00	0.21	0.06613
566	122	20011026	21.90	154.00	8.93	60.00	5.06E-05	5.13	3.17E-06	-1.06052	3.24	0.24	0.07007
567	125	20011220	8.35	159.45	8.52	26.50	0.0001252	4.39	2.17E-06	5.100989	1.57	0.26	0.04608
568	125	20011221	10.08	157.20	9.86	38.25	6.89E-05	5.29	1.43E-06	18.75643	1.72	0.31	0.05216
569	125	20011222	12.78	153.33	10.50	53.75	4.56E-05	7.40	6.49E-07	22.45998	2.14	0.32	0.06091
570	125	20011223	16.03	148.73	12.86	52.50	0.0001364	6.26	5.58E-07	29.90523	2.27	0.18	0.0606
571	125	20011224	19.88	146.70	11.73	42.50	7.18E-05	7.10	3.11E-06	-12.1595	3.53	0.33	0.06661
572	201	20020110	9.57	133.30	7.26	13.00	5.32E-05	4.44	1.51E-06	16.09083	1.55	0.54	0.04301
573	201	20020111	11.03	130.45	8.51	15.00	3.00E-05	5.30	-4.30E-07	20.71073	1.45	0.35	0.03584
574	201	20020112	12.20	128.00	10.65	15.00	2.47E-05	6.11	-6.00E-07	16.68345	1.37	0.30	0.03478
575	202	20020226	6.00	155.50	6.83	12.00	9.89E-05	1.45	3.88E-06	18.15925	0.39	0.25	0.03185
576	202	20020227	6.55	154.73	7.73	13.50	1.15E-04	2.83	4.38E-06	9.394244	0.49	0.21	0.03315
577	202	20020228	7.08	151.73	8.30	17.00	8.03E-05	5.71	2.80E-06	10.75079	0.66	0.28	0.03502
578	202	20020301	6.65	147.30	8.19	27.25	0.0001249	5.94	4.55E-06	11.58034	0.52	0.31	0.04355
579	202	20020302	7.75	141.93	7.78	33.25	9.88E-05	8.28	4.37E-07	8.854946	1.52	0.21	0.0438
580	202	20020303	9.80	136.10	10.94	38.75	9.85E-05	6.70	2.89E-06	11.87838	1.41	0.37	0.05531
581	202	20020304	11.58	132.23	9.56	45.00	0.0001043	4.60	1.39E-06	3.950187	1.71	0.60	0.06772
582	202	20020305	14.03	130.10	8.21	48.75	4.45E-05	3.41	-1.39E-07	28.31254	1.68	0.15	0.05015
583	202	20020306	16.38	130.75	8.79	46.25	4.51E-05	3.89	1.10E-06	-16.3404	1.94	0.20	0.05536
584	202	20020307	18.08	133.93	7.75	27.50	6.62E-06	4.41	-1.90E-06	-59.562	1.90	0.14	0.04166
585	202	20020308	17.17	135.17	9.13	15.00	4.10E-05	3.18	5.31E-09	-2.33128	1.37	0.25	0.03552
586	203	20020515	8.07	147.93	5.83	13.00	3.34E-05	7.71	6.13E-07	-17.51	0.70	0.11	0.02432
587	203	20020516	11.65	143.83	7.22	16.50	1.45E-05	7.79	4.69E-07	0.309159	1.72	0.40	0.03896
588	203	20020517	13.68	139.90	5.65	23.25	2.08E-05	4.03	1.73E-06	-6.04966	1.99	0.14	0.03769
589	203	20020518	16.38	139.55	6.78	32.50	4.20E-05	3.45	1.31E-07	1.791903	2.34	0.20	0.0463
590	203	20020519	19.03	141.75	8.51	43.75	4.84E-05	7.92	-1.17E-06	10.2794	3.55	0.35	0.06106
591	203	20020520	22.50	144.60	9.61	40.00	1.93E-05	11.41	2.37E-06	2.967417	5.11	0.49	0.07103
592	205	20020628	10.30	137.40	6.92	12.00	6.32E-05	2.57	1.14E-06	-9.22084	1.80	0.26	0.03694
593	205	20020629	11.23	135.75	10.85	18.25	9.787E-05	2.83	1.31E-06	-12.3648	1.69	0.31	0.04533
594	205	20020630	13.33	134.35	8.63	23.50	8.454E-05	3.96	-1.65E-06	-18.34	1.99	0.12	0.03998
595	205	20020701	16.88	132.28	10.04	29.00	7.22E-05	7.28	1.78E-06	-6.10582	2.37	0.37	0.05283
596	205	20020702	20.75	128.55	10.82	38.75	9.777E-05	6.72	3.13E-06	-5.871	3.02	0.46	0.06642
597	206	20020629	5.15	155.65	11.37	18.25	0.0003075	0.82	8.61E-06	0.024108	0.17	0.65	0.06159
598	206	20020630	5.70	155.55	14.83	22.25	0.0006556	2.01	1.49E-05	-0.09987	0.32	0.81	0.0867
599	206	20020701	6.90	154.45	10.08	23.00	1.40E-04	2.32	6.85E-06	4.255902	0.50	0.59	0.05521

600	206	20020702	7.63	152.58	7.91	23.00	9.57E-05	2.93	2.46E-06	0.196114	0.79	0.41	0.04543
601	206	20020703	8.80	150.08	7.53	23.50	8.94E-05	4.76	2.20E-06	-7.18563	0.43	0.28	0.0391
602	206	20020704	11.50	147.25	8.86	28.25	7.59E-05	6.79	7.22E-07	-7.6831	1.06	0.22	0.04109
603	206	20020705	14.53	143.20	7.24	39.50	5.25E-05	5.14	2.22E-06	2.647587	1.83	0.18	0.04856
604	206	20020706	16.88	139.70	9.23	45.00	7.31E-05	5.91	-1.33E-06	-1.35293	2.53	0.17	0.05407
605	206	20020707	19.80	135.58	10.42	48.75	0.0001104	5.81	6.74E-06	-10.5019	3.27	0.36	0.07312
606	206	20020708	22.20	133.45	8.79	50.00	3.83E-05	6.60	7.02E-07	-9.09197	3.50	0.17	0.06115
607	207	20020706	8.80	158.70	3.65	12.00	1.33E-05	3.88	1.05E-06	-0.36612	1.49	0.18	0.0282
608	207	20020707	8.78	156.85	5.99	13.50	3.92E-05	4.48	1.53E-07	3.680766	1.19	0.25	0.03114
609	207	20020708	10.05	152.50	4.72	18.00	3.21E-05	6.97	2.72E-07	-2.29499	0.97	0.36	0.03497
610	207	20020709	11.08	147.88	7.08	26.50	6.554E-05	5.44	2.11E-06	-14.4793	0.97	0.38	0.04526
611	207	20020710	12.20	143.75	7.30	32.25	4.33E-05	4.65	-6.20E-07	9.667756	1.74	0.27	0.04486
612	207	20020711	13.43	139.98	7.85	38.75	5.42E-05	5.82	-2.00E-07	11.65876	1.99	0.27	0.0499
613	207	20020712	16.20	136.15	9.44	41.25	8.52E-05	6.96	3.13E-06	11.59015	1.60	0.28	0.05359
614	207	20020713	20.80	131.53	9.56	45.00	6.96E-05	9.65	-1.84E-06	-0.73568	2.45	0.23	0.05391
615	209	20020719	15.65	159.40	6.74	52.50	3.17E-05	3.02	1.49E-06	-12.3581	2.80	0.16	0.05991
616	209	20020720	16.90	157.85	10.39	50.00	7.27E-05	4.87	1.00E-06	-14.2944	3.00	0.13	0.06098
617	209	20020721	20.18	154.80	12.72	53.75	0.0001014	6.26	4.96E-06	-4.93965	3.07	0.31	0.07277
618	209	20020722	22.20	152.30	7.20	55.00	2.97E-05	6.67	2.09E-06	0.258825	4.68	0.30	0.07222
619	211	20020723	22.65	130.85	7.14	30.75	3.09E-05	3.13	-2.12E-07	-2.28889	3.34	0.17	0.04899
620	211	20020724	21.10	131.20	6.31	31.25	2.40E-05	4.65	2.86E-07	1.566193	2.75	0.30	0.04947
621	211	20020725	21.25	134.20	8.00	30.00	2.93E-05	6.66	2.66E-06	6.310974	3.59	0.22	0.05109
622	213	20020811	9.97	158.77	7.47	15.00	7.076E-05	2.74	1.07E-06	-10.713	1.96	0.15	0.03629
623	213	20020812	11.60	157.68	9.25	20.25	1.11E-04	4.48	3.48E-06	-4.33988	2.28	0.20	0.04499
624	213	20020813	15.80	155.40	12.09	29.00	7.69E-05	8.10	2.75E-06	-6.31378	2.64	0.36	0.0554
625	213	20020814	19.20	150.15	10.44	37.00	5.95E-05	7.55	2.06E-06	4.584929	2.96	0.29	0.05651
626	213	20020815	22.00	145.73	12.11	41.67	8.37E-05	7.11	-5.70E-08	1.101464	4.31	0.24	0.06429
627	215	20020823	17.95	159.25	10.44	24.00	2.43E-05	8.38	-8.48E-07	-0.29806	2.75	0.30	0.04577
628	215	20020824	19.38	155.55	9.52	27.00	4.46E-05	5.82	-2.75E-06	4.702288	2.68	0.23	0.04452
629	215	20020825	21.23	150.28	10.46	32.00	6.35E-05	7.35	1.45E-06	3.095563	4.01	0.25	0.05695
630	215	20020826	22.43	145.60	7.79	41.67	3.45E-05	5.44	-1.44E-06	2.854487	4.56	0.26	0.06163
631	216	20020828	16.50	155.27	8.34	13.00	3.04E-05	3.30	-2.41E-07	-13.8956	2.53	0.12	0.03505
632	216	20020829	18.65	155.00	9.91	18.25	3.99E-05	3.90	-2.79E-07	9.343496	2.57	0.13	0.03786
633	216	20020830	21.83	152.90	10.12	27.75	6.85E-05	5.78	2.22E-06	21.58761	3.99	0.35	0.05757
634	221	20020926	15.68	155.55	7.96	12.75	2.36E-05	6.36	-6.69E-07	4.931442	2.68	0.23	0.03574
635	221	20020927	16.70	149.95	9.25	20.00	3.65E-05	8.30	2.47E-06	7.982233	2.38	0.36	0.04481
636	221	20020928	18.83	143.15	11.43	33.25	8.659E-05	8.99	2.64E-07	5.128275	3.18	0.20	0.05214
637	221	20020929	20.65	137.25	8.98	47.50	4.69E-05	5.35	1.83E-06	11.84322	3.20	0.33	0.06375
638	221	20020930	22.50	135.80	10.55	50.00	4.42E-05	5.21	1.26E-06	4.766228	4.24	0.19	0.06615
639	222	20021008	10.65	155.65	5.26	15.00	3.17E-05	5.85	-1.83E-08	-0.24743	1.75	0.19	0.03153
640	222	20021009	12.78	152.90	8.15	17.00	4.81E-05	5.91	2.02E-06	-4.63685	2.12	0.29	0.04105
641	222	20021010	15.58	150.25	9.89	22.00	6.10E-05	6.79	-4.23E-07	-16.3574	2.29	0.15	0.04052
642	222	20021011	20.78	148.80	11.48	25.75	8.13E-05	6.17	4.62E-06	-4.6909	3.40	0.29	0.0563
643	225	20021120	10.58	145.30	8.14	14.25	4.17E-05	8.67	1.27E-06	-5.74633	1.25	0.23	0.03203
644	225	20021121	12.58	139.58	6.26	21.50	4.24E-05	6.87	4.95E-07	-0.63501	1.91	0.24	0.03835
645	225	20021122	15.33	135.73	9.43	26.50	1.02E-04	5.55	5.89E-07	2.402008	1.66	0.27	0.0454
646	225	20021123	19.30	135.58	8.91	32.75	7.14E-05	7.27	2.30E-06	-17.1985	2.81	0.27	0.05415
647	225	20021124	22.80	137.50	13.21	35.00	0.0001698	10.43	6.28E-07	-16.0749	3.82	0.31	0.0644
648	226	20021204	8.33	159.15	6.64	22.00	7.94E-05	3.43	3.73E-06	7.706454	1.46	0.22	0.04035
649	226	20021205	8.30	155.70	11.47	29.00	0.0002805	5.61	5.06E-06	0.395257	0.81	0.32	0.05474
650	226	20021206	9.23	151.48	13.08	33.50	0.0003407	5.11	8.38E-06	7.620885	0.45	0.45	0.0646
651	226	20021207	10.93	147.68	15.31	41.25	0.0003385	5.58	5.21E-06	5.061781	0.87	0.53	0.07259
652	226	20021208	13.90	144.93	16.53	48.75	0.0003015	5.21	5.29E-06	8.408709	1.75	0.59	0.08228
653	226	20021209	17.58	143.85	17.24	45.00	0.0002298	6.50	3.15E-06	-0.14438	2.51	0.39	0.0737
654	226	20021210	21.50	146.60	14.82	42.50	9.90E-05	12.81	-3.34E-07	-6.26927	4.31	0.18	0.06283
655	301	20030115	8.20	159.70	8.82	15.00	1.30E-05	8.06	6.61E-07	21.80405	1.58	0.14	0.02851
656	301	20030116	9.83	155.50	9.12	15.00	1.83E-05	10.04	2.46E-06	22.80445	1.31	0.20	0.0296
657	301	20030117	12.55	149.20	10.55	15.00	3.77E-06	5.71	-3.62E-08	27.27142	2.10	0.31	0.03723
658	301	20030118	14.23	146.88	10.58	17.25	3.52E-05	2.72	2.91E-07	13.86844	1.82	0.29	0.04012
659	301	20030119	15.48	149.50	8.50	18.00	5.12E-05	6.11	8.39E-07	-15.9548	2.23	0.15	0.03779
660	301	20030120	17.35	154.28	8.72	15.75	1.17E-05	6.56	-2.05E-06	-15.2749	2.57	0.12	0.03398
661	301	20030121	19.00	157.00	6.29	10.00	1.96E-05	7.15	1.97E-07	-37.0863	2.85	0.18	0.03539
662	302	20030410	8.00	158.90	6.02	15.00	6.67E-05	4.68	1.89E-07	11.26235	1.20	0.09	0.02717
663	302	20030411	9.55	156.10	5.43	18.00	4.44E-05	4.42	2.36E-06	4.847993	1.28	0.14	0.03176
664	302	20030412	10.03	152.78	5.92	22.75	5.02E-05	4.34	1.90E-06	3.079403	1.02	0.28	0.03809
665	302	20030413	10.25	149.50	7.87	30.25	8.43E-05	4.34	3.43E-06	-1.48142	0.83	0.33	0.04669
666	302	20030414	10.73	145.63	7.99	38.75	7.563E-05	6.48	1.54E-06	-5.37253	1.13	0.16	0.04532
667	302	20030415	12.10	140.25	10.03	46.25	0.0001094	6.98	1.94E-06	-1.58696	1.90	0.30	0.05962
668	302	20030416	13.58	135.68	12.58	52.50	0.0001275	4.32	1.43E-06	-3.43042	1.86	0.50	0.07246
669	302	20030417	13.80	132.50	12.94	45.00	6.69E-05	3.94	-1.34E-07	-5.42594	2.00	0.33	0.06081
670	302	20030418	13.63	129.80	11.55	45.00	5.61E-05	2.48	2.33E-06	-3.10695	1.49	0.39	0.06121
671	302	20030419	14.90	128.08	11.29	42.50	4.68E-05	3.85	1.86E-06	8.547567	1.09	0.37	0.05537
672	303	20030519	7.10	150.30	7.65	14.25	6.63E-05	0.81	4.53E-06	-0.53434	0.38	0.29	0.03578
673	303	20030520	7.93	150.80	8.55	17.00	0.0001114	2.28	3.20E-06	3.435206	0.24	0.36	0.03944
674	303	20030521	9.78	150.48	8.85	25.25	7.75E-05	3.14	1.72E-06	2.714611	0.58	0.12	0.03544
675	303	20030522	12.90	151.20	9.69	30.00	8.88E-05	4.89	2.36E-06	-15.0296	2.18	0.18	0.049
676	303	20030523	16.13	151.38	12.31	37.50	0.0001362	4.29	2.64E-06	12.82731	2.39	0.27	0.05838
677	303	20030524	19.70	152.55	13.08	40.00	0.0001265	6.63	4.37E-06	12.41669	3.45	0.43	0.06992
678	303	20030525	22.40	154.80	13.56	40.00	0.000116	7.25	-2.02E-06	17.36085	3.39	0.44	0.06551
679	306	20030612	10.23	134.88	4.35	11.50	1.39E-05	6.48	1.84E-06	-7.78699	1.63	0.24	0.03085
680	306	20030613	11.30	130.38	5.94	16.50	2.79E-05	6.09	-4.59E-06	-10.3853	1.45	0.47	0.03815
681	306	20030614	11.40	127.50	8.68	20.00	6.60E-05	4.08	5.56E-06	-16.8149	1.33	0.43	0.04848
682	308	20030715	10.30	131.97	4.30	15.00	1.51E-05	4.59	-2.62E-07	-6.71672	1.57	0.31	0.03413
683	308	20030716	10.63	129.17	6.19	15.00	4.59E-05	4.93	1.16E-06	-3.22643	1.38	0.62	0.04605
684	307	20030715	6.10	146.80	3.32	12.00	2.79E-05	6.34	2.59E-06	-8.73831	0.61	0.20	0.02574
685	307	20030716	6.08	143.60	4.77	12.75	3.83E-05	5.78	1.31E-07	-0.30995	0.50	0.35	0.03009
686	307	20030717	8.45	140.53	5.02	17.75	3.47E-05	5.91	1.13E-07	-6.54479	1.81	0.15	0.03246
687	307	20030718	10.18	137.28	9.50	22.00	9.13E-05	3.27	2.74E-06	-2.04023	1.72	0.39	0.04916
688	307	20030719	10.65	134.50	11.37	30.25	0.0001782	4.05	4.82E-06	-7.78553	1.70	0.33	0.05708
689	307	20030720	12.35	131.15	13.19	41.25	0.0002525	6.35	4.55E-06	-0.10835	1.62	0.38	0.06674
690	307	20030721	13.80	127.65	15.50	47.50	0.0003258	6.66	7.75E-06	3.851108	1.13	0.43	0.07494
691	309	20030731	15.40	127.50	3.70	12.00	4.94E-06	3.95	-4.92E-06	-2.97657	1.10	0.46	0.0319
692	309	20030801	16.78	127.70	4.38	14.25	6.53E-06	3.61	-1.52E-06	-6.32305	1.24	0.24	0.02954
693	310	20030802	12.40	140.30	5.85	12.00	1.74E-05	2.54	-1.19E-07	-13.7731	1.94	0.21	0.03347
694	310	20030803	13.70	139.03	6.81	16.25	2.77E-05	5.36	1.61E-06	-7.09873	2.02	0.15	0.03453
695	310	20030804	16.25	135.00	7.90	29.00	5.789E-05	6.32	4.82E-06	-14.7829	1.50	0.27	0.04697
696	310	20030805	19.23	131.38	7.83	38.75	4.73E-05	5.95	-3.17E-07	-10.6741	2.38	0.21	0.05072
697	310	20030806	22.15	129.15	10.99	40.00	9.398E-05	7.46	2.18E-06	-15.166	3.05	0.35	0.06349
698	312	20030815	9.93	149.93	4.10	12.00	6.06E-06	4.88	5.30E-07	3.20153	0.70	0.27	0.02687
699	312	20030816	11.80	146.55	5.78	12.00	5.99E-06	6.33	-8.52E-07	3.472805	1.23	0.24	0.02752

700	312	20030817	14.38	142.88	5.77	12.00	5.81E-06	5.25	-5.26E-07	7.263776	1.83	0.12	0.02663
701	312	20030818	17.10	139.83	7.00	12.00	1.12E-05	5.82	-4.31E-06	9.303907	2.63	0.27	0.03323
702	312	20030819	19.45	135.43	6.25	14.25	2.02E-05	5.63	4.30E-07	-2.42612	2.97	0.41	0.04404
703	312	20030820	19.10	131.68	5.71	17.00	2.09E-05	4.27	2.58E-06	0.502019	2.28	0.30	0.04052
704	312	20030821	18.30	128.23	5.82	25.33	2.47E-05	6.23	-1.43E-06	6.480415	1.35	0.19	0.03426
705	313	20030828	16.78	137.18	5.29	12.75	1.65E-05	3.67	8.96E-08	-33.4114	2.10	0.21	0.03516
706	313	20030829	16.00	135.28	5.63	15.75	2.12E-05	1.89	-2.21E-07	-13.3997	1.52	0.29	0.03642
707	313	20030830	17.08	133.50	9.02	27.75	8.327E-05	5.70	4.68E-06	2.262693	1.93	0.31	0.05001
708	313	20030831	19.70	129.67	8.95	34.33	7.222E-05	7.12	-3.56E-07	3.017942	2.08	0.45	0.05499
709	314	20030904	10.97	149.83	5.73	10.67	8.62E-06	7.18	-1.68E-06	-10.7315	1.22	0.20	0.02575
710	314	20030905	13.38	145.68	3.82	12.75	7.55E-06	7.40	-4.34E-09	-9.26933	1.71	0.15	0.02697
711	314	20030906	16.03	140.53	4.41	17.75	1.37E-05	6.48	-1.40E-06	-8.12343	2.16	0.23	0.03441
712	314	20030907	18.15	136.43	8.98	22.00	3.96E-05	5.73	-3.47E-06	-22.7946	2.01	0.29	0.04186
713	314	20030908	20.35	132.48	6.65	29.00	2.99E-05	5.20	4.40E-06	-9.71188	2.36	0.31	0.05028
714	314	20030909	22.43	129.57	6.26	40.00	2.11E-05	4.37	1.59E-06	-16.3679	3.00	0.25	0.05597
715	315	20030916	14.25	129.93	4.76	15.00	8.67E-06	7.58	1.19E-06	-12.2046	1.60	0.12	0.0283
716	315	20030917	18.08	128.98	8.20	15.75	2.34E-05	7.89	1.79E-06	-11.1255	1.22	0.27	0.03472
717	315	20030918	22.07	128.13	10.61	20.33	3.74E-05	4.29	-1.25E-06	11.63598	3.35	0.31	0.04766
718	316	20030924	16.15	138.40	8.25	12.00	1.61E-05	5.51	-1.61E-06	-3.161	2.39	0.26	0.03557
719	316	20030925	16.38	136.03	5.70	15.00	2.36E-05	1.12	-1.55E-06	-7.03913	1.57	0.24	0.03377
720	316	20030926	18.58	137.30	6.85	15.00	3.20E-05	7.03	4.08E-06	3.29584	2.54	0.23	0.03888
721	316	20030927	22.00	139.30	6.56	20.50	2.48E-05	5.21	-1.12E-06	-8.36538	4.17	0.32	0.05001
722	317	20031017	14.97	132.03	4.38	10.00	1.55E-05	1.14	6.60E-07	-6.88256	2.32	0.16	0.03197
723	317	20031018	15.00	131.60	5.57	13.75	2.63E-05	2.48	9.02E-07	-3.86568	2.39	0.18	0.03536
724	317	20031019	15.53	130.25	8.65	19.50	6.542E-05	1.41	1.44E-06	8.054852	1.73	0.30	0.04267
725	317	20031020	15.83	130.95	7.96	32.75	8.05E-05	1.15	2.73E-06	-0.02384	2.22	0.24	0.05203
726	317	20031021	16.73	131.18	8.42	42.50	7.01E-05	1.45	3.82E-06	-4.71447	2.16	0.27	0.05896
727	317	20031022	17.93	131.30	9.23	45.00	7.03E-05	2.18	3.23E-06	-18.1749	2.05	0.37	0.06387
728	317	20031023	19.40	132.68	12.67	43.75	0.0001657	2.86	6.63E-06	0.86787	2.35	0.38	0.07039
729	317	20031024	21.53	134.05	11.98	40.00	9.294E-05	5.34	5.64E-06	-10.6163	3.62	0.39	0.07036
730	318	20031019	18.35	144.60	4.81	10.00	1.68E-05	2.19	5.44E-07	-17.324	2.94	0.23	0.03744
731	318	20031020	20.00	143.60	5.08	13.75	1.88E-05	3.66	-1.13E-06	-5.78377	4.12	0.29	0.04438
732	318	20031021	21.70	145.00	6.39	19.75	2.65E-05	3.54	3.49E-06	-3.43079	4.47	0.35	0.05487
733	318	20031022	22.70	146.70	7.75	27.50	4.20E-05	2.80	-2.42E-06	2.673814	4.64	0.22	0.05355
734	318	20031028	22.05	155.05	10.61	32.00	4.26E-05	9.91	3.35E-07	9.736783	3.13	0.24	0.04985
735	319	20031029	12.55	131.20	6.80	11.00	8.37E-06	3.59	-1.32E-06	23.90935	1.67	0.26	0.02963
736	319	20031030	13.10	128.50	6.59	14.25	1.31E-05	4.60	-2.80E-07	13.51451	1.21	0.21	0.02881
737	320	20031111	10.80	139.50	8.43	12.00	1.36E-05	8.01	4.02E-08	6.336187	1.77	0.29	0.03306
738	320	20031112	11.80	135.03	8.90	14.25	2.22E-05	9.97	2.00E-06	8.448753	1.84	0.52	0.04279
739	320	20031113	12.30	129.25	9.06	21.50	4.07E-05	9.57	-4.90E-07	2.045758	1.43	0.38	0.04027
740	321	20031121	8.73	157.45	7.39	17.00	2.88E-05	7.79	1.23E-06	-2.868	1.23	0.13	0.02954
741	321	20031122	8.68	151.45	5.75	26.50	5.852E-05	7.05	2.51E-06	-3.64633	0.37	0.20	0.03467
742	321	20031123	8.00	146.25	8.42	32.50	0.0001216	5.39	2.56E-06	6.79928	0.93	0.18	0.04352
743	321	20031124	8.83	142.80	11.22	36.25	0.0002535	5.77	6.48E-06	-1.49493	1.58	0.47	0.06683
744	321	20031125	11.48	138.53	14.98	41.25	0.0003735	5.34	5.96E-06	-4.42624	1.79	0.46	0.07647
745	321	20031126	13.23	136.10	15.78	48.75	0.0003825	3.12	7.10E-06	-16.5475	1.87	0.54	0.08697
746	321	20031127	14.65	134.08	16.74	58.75	0.0004248	3.09	4.41E-06	-5.78872	1.89	0.34	0.08604
747	321	20031128	16.50	132.10	16.87	50.00	0.0003846	4.44	7.90E-06	-16.3258	2.53	0.51	0.09033
748	321	20031129	19.60	131.18	17.10	45.00	0.0003077	5.07	4.30E-06	-17.0686	2.44	0.66	0.08705
749	321	20031130	22.40	133.20	19.58	42.50	0.0003995	8.40	1.06E-06	-13.2673	3.47	0.35	0.08091
750	401	20040402	6.40	152.80	4.67	10.00	3.45E-05	2.61	5.05E-07	-5.48086	0.92	0.29	0.03006
751	401	20040403	6.45	151.65	4.87	11.50	4.45E-05	2.26	3.03E-06	-8.56909	0.73	0.28	0.03191
752	401	20040404	7.03	149.90	5.65	15.00	7.04E-05	3.29	3.32E-06	-0.30916	0.40	0.23	0.03147
753	401	20040405	9.00	148.55	8.55	20.25	6.66E-05	3.43	4.11E-06	3.531755	0.81	0.20	0.03702
754	401	20040406	9.48	145.88	10.45	27.00	0.0001243	3.95	4.10E-06	-8.34835	1.09	0.37	0.05129
755	401	20040407	9.73	142.70	11.45	30.75	0.0001847	4.65	3.31E-06	-9.64804	1.70	0.26	0.05449
756	401	20040408	8.90	139.50	13.65	38.75	0.0003481	3.44	9.67E-06	3.672411	1.79	0.56	0.07938
757	401	20040409	9.70	136.90	17.31	47.50	0.000614	4.19	1.08E-05	0.097225	1.47	0.78	0.10191
758	401	20040410	11.60	133.98	17.87	50.00	0.000587	4.91	5.13E-06	0.633948	1.84	0.67	0.09703
759	401	20040411	13.93	131.60	18.75	45.00	0.0004769	3.28	5.89E-06	-0.82601	2.03	0.58	0.09015
760	401	20040412	15.95	131.10	17.73	45.00	0.0003785	2.45	3.74E-06	5.648839	2.34	0.45	0.08193
761	401	20040413	18.28	132.30	17.49	45.00	0.0003867	4.98	7.52E-06	0.427577	1.92	0.54	0.08455
762	401	20040414	21.40	135.10	17.19	41.67	0.0002424	8.08	4.17E-06	-0.5105	3.97	0.66	0.08701
763	402	20040513	7.60	132.30	4.89	11.75	4.02E-05	1.74	4.93E-06	0.302006	1.32	0.26	0.03468
764	402	20040514	8.73	131.20	5.79	24.75	4.94E-05	3.16	2.83E-06	-2.47619	1.42	0.18	0.03918
765	402	20040515	10.08	128.83	11.44	33.75	0.0001879	3.73	5.78E-06	-11.7682	1.34	0.49	0.0642
766	402	20040516	11.10	127.20	14.74	45.00	0.000177	4.09	1.86E-06	1.842502	1.30	0.34	0.06356
767	402	20040519	22.50	128.00	15.85	40.00	0.0002065	4.09	2.93E-06	-1.6878	3.29	0.67	0.08255
768	403	20040516	6.45	141.15	5.73	10.00	1.98E-05	2.77	6.42E-06	-4.86071	1.61	0.60	0.0465
769	403	20040517	7.53	139.63	4.16	12.75	2.14E-05	3.39	5.62E-06	3.759711	1.73	0.39	0.03963
770	403	20040518	9.18	137.00	6.58	18.50	4.33E-05	4.97	3.97E-07	9.183066	1.31	0.48	0.04212
771	403	20040519	11.08	133.78	6.10	22.50	2.15E-05	4.46	-4.32E-07	-10.1593	1.81	0.49	0.04686
772	403	20040520	14.05	133.70	7.94	22.25	1.91E-05	4.96	2.31E-07	1.843032	1.97	0.18	0.03759
773	403	20040521	17.10	134.80	6.58	15.00	9.80E-06	3.78	-1.48E-06	-3.32339	1.45	0.27	0.03303
774	403	20040522	19.20	134.80	4.98	12.00	5.24E-06	5.22	-9.30E-07	-4.42121	2.72	0.22	0.03371
775	406	20040611	6.50	139.10	3.27	12.00	1.47E-05	3.43	5.40E-06	-0.42268	1.79	0.61	0.04591
776	406	20040612	7.50	137.80	4.29	12.00	2.47E-05	2.74	6.11E-06	-0.24914	1.44	0.37	0.03841
777	406	20040613	8.93	136.58	5.07	17.00	4.19E-05	2.43	2.47E-06	-2.83743	1.21	0.29	0.03681
778	406	20040614	9.83	136.95	8.23	27.25	9.767E-05	2.85	6.30E-06	-10.6322	1.54	0.35	0.0525
779	406	20040615	12.33	137.05	11.92	45.00	0.0002114	4.12	1.83E-06	-39.7713	1.84	0.23	0.06473
780	406	20040616	15.40	135.83	14.19	60.00	0.0002059	4.60	5.08E-06	-25.2082	1.66	0.28	0.0759
781	406	20040617	17.55	133.20	12.89	57.50	0.0001365	3.83	4.13E-06	-12.5576	1.92	0.30	0.07195
782	406	20040618	19.80	130.70	15.28	56.25	0.0002238	5.53	2.30E-06	2.566257	2.47	0.42	0.07921
783	406	20040619	22.20	129.40	19.98	50.00	0.0004744	5.32	3.91E-06	1.39797	3.06	0.70	0.09961
784	407	20040621	14.60	146.00	3.95	12.00	1.23E-05	2.55	7.72E-08	-0.21814	1.93	0.18	0.0303
785	407	20040622	15.08	144.70	5.13	14.25	2.18E-05	4.13	3.67E-07	0.254499	1.83	0.12	0.02962
786	407	20040623	16.60	142.23	8.13	19.00	4.08E-05	3.64	1.68E-07	1.25518	2.28	0.24	0.04063
787	407	20040624	16.00	138.00	9.92	26.00	7.445E-05	7.28	1.44E-06	16.49123	2.30	0.31	0.04719
788	407	20040625	14.95	133.18	9.84	30.00	7.48E-05	4.30	2.14E-06	2.334719	2.03	0.35	0.05225
789	407	20040626	15.15	130.28	8.81	30.00	8.03E-05	3.46	2.55E-06	-25.7259	1.83	0.24	0.04983
790	407	20040627	16.87	127.97	14.77	32.67	0.0002673	5.39	6.86E-07	-21.0204	1.21	0.25	0.05677
791	408	20040625	11.55	152.45	6.87	12.75	3.71E-05	2.75	-4.82E-07	-1.08431	1.67	0.12	0.02965
792	408	20040626	12.15	150.60	8.16	20.25	7.858E-05	3.71	1.44E-07	-11.2479	2.02	0.09	0.03733
793	408	20040627	14.35	148.03	7.19	27.75	5.32E-05	4.91	2.31E-06	-5.43002	1.98	0.17	0.04302
794	408	20040628	17.10	145.33	9.73	31.50	8.904E-05	4.81	2.45E-06	-15.099	2.61	0.22	0.0531
795	408	20040629	20.50	143.05	15.17	34.50	0.0002119	6.30	7.82E-06	-1.05696	4.36	0.59	0.08325
796	409	20040711	19.23	135.40	2.72	10.67	5.37E-06	4.98	-1.74E-06	-6.7705	2.77	0.33	0.03532
797	409	20040712	20.35	131.85	4.49	12.00	1.04E-05	4.40	1.66E-07	-5.1349	2.25	0.27	0.03435
798	409	20040713	20.87	129.10	6.26	14.00	2.08E-05	6.38	-2.55E-07	-10.2293	2.88	0.38	0.04239
799	414	20040806	15.43	134.00	6.55	13.50	1.82E-05	7.26	7.48E-07	-8.3636	1.83	0.23	0.03285

800	414	20040807	15.98	130.45	7.49	15.00	2.96E-05	2.41	1.60E-06	-14.0767	1.82	0.28	0.03914
801	414	20040808	17.98	130.20	9.30	17.50	5.08E-05	3.37	-2.29E-06	-4.71394	1.72	0.19	0.03578
802	414	20040809	20.23	129.73	9.01	24.00	6.308E-05	3.57	7.76E-07	-4.23603	2.43	0.29	0.04768
803	414	20040810	21.97	127.90	6.14	29.33	2.38E-05	3.50	-2.98E-06	-1.03185	3.39	0.34	0.05117
804	416	20040814	15.20	138.23	4.57	13.50	1.29E-05	7.79	-1.43E-07	-22.6327	2.16	0.24	0.03371
805	416	20040815	17.73	133.83	5.51	15.00	1.42E-05	5.12	1.92E-06	-13.2024	1.81	0.23	0.03479
806	416	20040816	19.55	130.20	9.30	20.25	6.03E-05	7.68	2.86E-06	0.091397	2.28	0.36	0.04644
807	416	20040817	23.00	127.80	6.06	23.00	2.74E-05	10.39	3.20E-07	6.197547	3.35	0.23	0.04224
808	417	20040819	13.60	159.10	6.22	23.00	4.65E-05	6.69	4.17E-07	-0.03391	2.78	0.27	0.04406
809	417	20040820	14.23	156.08	8.45	24.00	7.457E-05	6.07	8.07E-07	-1.45996	2.76	0.21	0.04525
810	417	20040821	14.33	150.90	9.52	30.25	8.725E-05	7.02	2.13E-06	4.847993	2.35	0.27	0.05014
811	417	20040822	14.40	146.03	12.30	46.25	0.0001218	4.98	3.21E-06	-9.06352	1.85	0.37	0.06541
812	417	20040823	16.03	142.43	12.63	60.00	0.0001702	5.09	4.08E-06	-9.2451	2.11	0.25	0.07292
813	417	20040824	18.33	139.40	16.01	60.00	0.0002626	4.62	2.53E-06	-15.1316	3.00	0.24	0.08113
814	417	20040825	21.50	137.45	17.76	60.00	0.0003185	4.08	3.93E-06	-12.9083	3.28	0.38	0.09077
815	418	20040818	10.23	140.48	8.38	15.00	3.10E-05	4.39	-6.64E-08	1.346027	1.89	0.28	0.0371
816	418	20040819	12.03	137.80	7.67	15.00	2.33E-05	4.96	-1.76E-07	-7.87203	1.86	0.14	0.03214
817	418	20040820	15.23	135.35	7.97	21.00	4.05E-05	5.62	-1.95E-06	-2.27617	1.69	0.25	0.03754
818	418	20040821	18.58	132.38	7.30	28.25	4.64E-05	6.20	-4.67E-07	9.839041	1.97	0.20	0.04072
819	418	20040822	21.20	128.43	6.78	31.50	3.70E-05	5.46	3.22E-06	3.138744	3.23	0.31	0.05411
820	419	20040829	14.17	157.27	7.00	28.00	4.12E-05	7.61	1.72E-06	-1.47427	2.84	0.28	0.04814
821	419	20040830	15.48	152.30	7.83	33.25	3.91E-05	6.36	9.21E-07	-0.07285	2.45	0.30	0.05063
822	419	20040831	17.03	148.08	10.18	48.75	9.133E-05	4.88	3.36E-07	8.158668	2.52	0.24	0.06033
823	419	20040901	19.70	144.40	12.58	50.00	0.0001596	7.34	3.62E-06	2.631162	3.62	0.26	0.07145
824	419	20040902	21.60	139.20	13.17	47.50	1.53E-04	5.96	3.81E-06	-14.5532	3.93	0.24	0.0727
825	419	20040903	22.35	134.68	11.04	45.00	0.0001046	4.86	-1.33E-06	-11.1146	4.13	0.30	0.06869
826	420	20040903	14.00	155.60	6.86	15.00	1.31E-05	9.91	2.58E-06	6.528927	2.65	0.22	0.03589
827	420	20040904	16.00	152.13	8.00	15.75	1.85E-05	7.19	3.73E-07	11.29185	2.45	0.33	0.03952
828	420	20040905	18.23	146.50	7.21	26.00	1.30E-05	7.64	-3.19E-06	8.216092	2.81	0.24	0.04114
829	420	20040906	19.83	140.75	7.85	25.00	2.92E-05	6.67	-2.28E-06	0.192521	3.74	0.19	0.04557
830	420	20040907	22.30	138.40	9.21	21.50	2.91E-05	5.87	-1.73E-06	2.718055	3.92	0.24	0.0471
831	422	20040919	12.38	148.10	4.23	11.00	1.37E-05	5.38	1.09E-06	-0.56321	1.70	0.25	0.03042
832	422	20040920	12.75	143.88	6.40	15.75	2.03E-05	4.22	-2.09E-06	-0.32698	1.79	0.27	0.03435
833	422	20040921	13.83	141.10	6.73	21.00	3.47E-05	3.88	2.43E-06	4.75951	1.90	0.22	0.03933
834	422	20040922	15.80	138.88	8.23	29.00	3.73E-05	3.93	1.83E-06	4.500686	2.30	0.25	0.04722
835	422	20040923	18.03	136.30	8.71	38.25	2.60E-05	4.77	1.58E-06	11.03912	1.90	0.20	0.04799
836	422	20040924	20.30	133.05	9.89	43.75	5.913E-05	5.25	-2.32E-06	25.91769	2.68	0.35	0.05787
837	422	20040925	22.20	131.20	8.08	45.00	3.45E-05	5.70	9.06E-08	16.19945	3.37	0.31	0.06009
838	423	20041003	16.50	134.90	4.28	15.00	1.49E-05	1.32	-2.08E-06	-6.64846	1.50	0.25	0.03236
839	423	20041004	16.75	134.45	6.76	18.75	1.80E-05	1.86	-3.50E-06	-8.37007	1.59	0.21	0.03406
840	423	20041005	19.13	134.50	6.76	22.25	1.71E-05	4.10	2.98E-06	-9.73362	2.67	0.19	0.04294
841	423	20041006	20.90	132.48	8.85	29.50	4.27E-05	3.50	-5.05E-07	-6.31908	2.42	0.18	0.04587
842	423	20041007	22.23	130.77	7.51	41.67	3.00E-05	2.57	9.13E-07	7.950973	3.33	0.28	0.05909
843	424	20041012	13.20	148.40	5.95	15.75	3.25E-05	9.54	8.55E-07	-14.656	2.03	0.17	0.03267
844	424	20041013	14.30	141.30	8.02	24.00	5.16E-05	7.17	-1.12E-08	-15.6691	1.89	0.27	0.04265
845	424	20041014	13.80	137.15	9.41	31.50	9.656E-05	3.68	2.31E-06	-10.7368	1.84	0.22	0.04995
846	424	20041015	15.28	134.70	9.63	37.50	8.69E-05	3.56	2.89E-06	-7.796	1.70	0.22	0.05255
847	424	20041016	17.75	133.30	9.71	43.75	9.46E-05	4.53	1.87E-06	-7.14845	1.87	0.16	0.05419
848	424	20041017	20.00	130.48	13.70	50.00	0.0001887	5.76	3.89E-06	-4.55031	2.51	0.33	0.07206
849	424	20041018	22.00	127.85	15.20	45.00	0.0002343	3.92	2.41E-06	-5.9122	3.39	0.55	0.08246
850	425	20041015	11.70	159.30	8.08	15.00	1.12E-05	4.24	5.53E-07	9.206682	2.43	0.33	0.04013
851	425	20041016	11.08	155.93	7.10	17.75	2.18E-05	4.39	-3.06E-07	5.86819	1.95	0.21	0.03538
852	425	20041017	10.58	153.53	7.68	23.00	5.92E-05	2.36	2.59E-06	9.563526	1.36	0.16	0.03814
853	425	20041018	10.23	151.28	8.67	32.00	0.0001091	4.54	1.56E-06	6.122511	0.78	0.39	0.04905
854	425	20041019	9.83	147.55	9.37	35.00	0.0001379	5.21	2.39E-06	8.867588	0.89	0.27	0.04862
855	425	20041020	11.58	143.45	8.42	40.00	9.05E-05	6.69	2.28E-06	23.15327	1.70	0.13	0.04664
856	425	20041021	13.85	138.83	8.91	36.25	7.603E-05	5.67	4.33E-06	14.17415	2.01	0.31	0.05389
857	425	20041022	16.08	135.20	9.35	38.75	7.985E-05	5.96	4.05E-06	7.640754	1.50	0.27	0.0523
858	425	20041023	18.33	130.05	10.73	45.00	6.46E-05	8.28	2.93E-06	18.5588	1.78	0.25	0.05418
859	426	20041113	8.90	134.60	7.88	10.00	3.06E-05	7.97	7.67E-07	5.109996	1.39	0.22	0.02858
860	426	20041114	11.48	130.50	7.08	15.75	2.40E-05	11.06	-4.62E-07	18.75184	1.45	0.20	0.02803
861	428	20041128	5.85	151.35	5.19	13.00	6.44E-05	7.80	3.25E-06	1.912175	0.51	0.28	0.03002
862	428	20041129	6.93	145.28	9.74	20.50	0.000143	9.09	2.72E-06	8.310201	0.86	0.13	0.03466
863	428	20041130	9.68	138.08	9.91	31.50	0.0001335	11.09	3.96E-06	0.637704	1.72	0.40	0.05358
864	428	20041201	12.37	130.67	12.85	43.33	0.0001614	10.59	2.91E-06	15.08865	1.57	0.23	0.05537
865	429	20041211	9.65	158.15	9.39	20.00	3.14E-05	9.74	1.27E-06	-6.3159	1.70	0.18	0.03564
866	429	20041212	9.98	152.20	9.35	20.00	4.37E-05	10.66	8.40E-07	10.01521	0.88	0.18	0.03065
867	429	20041213	10.38	144.53	7.55	20.00	2.91E-05	8.41	1.89E-06	11.08813	1.56	0.30	0.03759
868	429	20041214	11.33	138.80	7.25	20.00	2.54E-05	5.45	-2.35E-07	6.832226	1.78	0.34	0.03971
869	429	20041215	11.70	135.08	7.05	17.00	2.48E-05	4.28	1.28E-06	4.583605	1.82	0.26	0.03707
870	429	20041216	12.35	132.33	6.29	15.75	2.87E-05	2.50	-1.22E-06	0.22412	1.82	0.15	0.03216
871	429	20041217	13.63	131.45	7.91	18.00	1.09E-05	1.78	-2.88E-07	11.57849	1.94	0.20	0.03571
872	429	20041218	15.75	131.08	7.27	18.00	1.39E-05	4.38	3.04E-06	9.612314	2.33	0.40	0.04466
873	429	20041219	18.40	130.80	8.39	15.00	1.40E-05	3.24	-3.31E-06	-7.92872	2.10	0.26	0.03617
874	430	20041217	12.48	152.48	8.04	12.00	2.18E-05	4.34	1.11E-06	-0.14729	2.02	0.31	0.03727
875	430	20041218	13.55	149.63	7.66	15.75	2.49E-05	4.03	1.95E-06	14.13309	2.24	0.27	0.03884
876	430	20041219	14.63	147.03	6.40	19.00	4.09E-05	5.67	5.17E-07	20.56238	1.95	0.19	0.03436
877	430	20041220	19.30	146.85	10.93	23.00	6.07E-05	8.74	3.21E-08	45.99129	3.16	0.10	0.03891
878	430	20041221	22.90	150.00	12.61	23.00	1.30E-05	13.64	1.37E-06	14.3788	4.35	0.17	0.04683
879	501	20050114	6.13	148.18	8.44	15.00	7.34E-05	6.16	4.61E-06	11.89401	0.25	0.27	0.03258
880	501	20050115	8.88	147.00	10.26	17.00	3.03E-05	6.17	9.36E-07	13.31345	0.97	0.38	0.03758
881	501	20050116	12.65	146.30	7.16	22.25	3.59E-05	4.89	1.16E-06	-11.2977	1.53	0.22	0.03871
882	501	20050117	15.28	149.15	6.96	23.50	5.19E-05	5.84	1.01E-06	-21.1076	2.17	0.24	0.04339
883	501	20050118	17.40	152.80	8.20	24.50	6.02E-05	4.08	3.98E-06	-24.4164	2.62	0.22	0.04911
884	501	20050119	18.67	155.10	9.74	15.00	6.23E-05	3.63	1.77E-06	-27.5289	2.55	0.24	0.04405
885	502	20050312	6.50	150.50	7.54	10.00	2.06E-05	4.09	3.22E-06	-2.3448	0.38	0.29	0.02971
886	502	20050313	8.23	146.20	6.50	13.00	2.60E-05	8.36	3.08E-06	-3.54804	0.98	0.20	0.0289
887	502	20050314	10.45	140.60	7.69	15.00	2.52E-05	6.70	-3.46E-07	13.08988	1.88	0.35	0.03694
888	502	20050315	12.03	135.23	9.61	20.25	3.23E-05	8.29	-2.38E-07	14.66902	1.85	0.46	0.044
889	502	20050316	11.75	130.00	9.21	27.50	3.05E-05	11.11	-1.45E-06	22.82247	1.46	0.39	0.04127
890	503	20050421	9.73	140.13	5.23	12.00	2.64E-05	8.60	3.48E-06	8.04484	1.86	0.20	0.03053
891	503	20050422	11.33	135.33	5.55	14.25	2.86E-05	6.05	1.08E-06	4.536185	1.74	0.36	0.03668
892	503	20050423	12.38	131.85	3.83	26.00	1.92E-05	3.55	1.41E-06	-15.1914	1.75	0.24	0.04105
893	503	20050424	14.30	130.85	5.07	41.25	2.65E-05	3.27	1.01E-06	4.524528	2.00	0.38	0.05456
894	503	20050425	17.20	131.95	8.58	45.00	4.46E-05	6.20	9.88E-07	11.92765	2.27	0.16	0.05162
895	503	20050426	20.85	134.85	9.19	42.50	2.56E-05	11.96	4.41E-06	4.540953	3.74	0.37	0.06327
896	504	20050530	9.15	147.10	6.33	12.75	8.58E-06	3.79	7.21E-07	0.416451	0.98	0.20	0.02813
897	504	20050531	10.10	144.68	7.59	17.75	2.11E-05	3.85	5.03E-07	7.308283	1.53	0.40	0.04043
898	504	20050601	10.78	141.13	5.64	30.25	2.18E-05	5.53	1.80E-06	-6.76918	1.81	0.43	0.0496
899	504	20050602	11.60	136.90	3.84	42.50	1.79E-05	5.38	-1.98E-06	0.087953	1.90	0.44	0.0537

900	504	20050603	13.25	133.13	4.65	45.00	2.50E-05	4.82	1.23E-06	-16.1836	1.95	0.28	0.05383
901	504	20050604	15.45	130.85	6.86	48.75	3.77E-05	3.81	3.22E-07	-12.4212	2.14	0.15	0.05397
902	504	20050605	18.50	131.10	7.85	42.50	4.39E-05	4.98	1.24E-06	-4.95036	2.22	0.43	0.0604
903	504	20050606	21.50	133.45	13.57	43.75	0.0001565	3.75	-3.06E-07	-7.63228	3.18	0.28	0.06712
904	504	20050607	22.90	134.20	12.59	40.00	0.0001067	3.11	4.84E-08	-3.94038	4.01	0.44	0.07195
905	505	20050710	22.10	154.10	4.83	12.00	8.54E-06	2.39	-1.96E-06	-8.13218	3.27	0.26	0.03833
906	505	20050711	22.35	152.93	7.15	12.75	1.86E-05	5.46	-3.40E-06	-9.9662	4.27	0.31	0.04437
907	505	20050713	22.05	147.65	6.85	24.75	3.39E-05	6.27	-4.08E-06	0.334326	4.36	0.37	0.05269
908	505	20050714	19.88	141.93	8.28	36.25	4.71E-05	9.58	8.54E-07	1.99271	4.00	0.20	0.0547
909	505	20050715	19.25	134.30	9.39	45.00	9.868E-05	8.01	-1.48E-06	-1.03025	2.82	0.16	0.05525
910	505	20050716	20.30	129.07	8.38	55.00	5.54E-05	6.92	1.13E-06	-7.52604	2.54	0.29	0.06427
911	507	20050720	13.25	137.90	6.92	13.50	3.80E-05	0.92	1.63E-09	-12.3234	1.86	0.19	0.0352
912	507	20050721	14.13	137.40	6.12	15.75	3.69E-05	3.13	2.03E-06	-13.5333	1.89	0.18	0.036
913	507	20050722	16.98	137.75	6.67	19.50	3.93E-05	4.69	-6.21E-07	-12.8936	2.34	0.26	0.04103
914	507	20050723	19.88	137.03	8.09	24.75	4.76E-05	3.56	3.36E-06	-12.2726	2.95	0.27	0.05073
915	507	20050724	21.70	137.20	9.18	28.00	6.80E-05	6.32	1.51E-07	-0.54308	3.44	0.48	0.05889
916	509	20050730	9.45	137.40	3.36	13.50	1.92E-05	6.18	1.51E-06	-23.944	1.46	0.26	0.03234
917	509	20050731	11.50	134.45	7.67	16.50	4.79E-05	6.11	5.70E-07	-19.4116	1.80	0.17	0.03528
918	509	20050801	14.98	131.33	6.70	22.75	4.42E-05	5.81	2.57E-06	-11.6704	2.32	0.21	0.0427
919	509	20050802	18.48	128.33	8.68	31.50	4.43E-05	6.41	2.59E-06	-8.24185	1.38	0.36	0.04905
920	510	20050809	14.00	130.60	5.29	15.00	2.83E-05	2.93	2.29E-06	-26.4791	1.85	0.19	0.03631
921	510	20050810	14.85	129.03	8.08	15.00	5.34E-05	4.54	2.50E-06	-23.7115	1.29	0.25	0.03731
922	511	20050819	20.50	142.60	7.20	15.00	1.24E-05	1.24	-1.38E-06	-10.0795	4.36	0.33	0.04946
923	511	20050820	21.15	141.68	6.80	22.00	2.29E-05	2.30	-2.22E-06	-6.72626	4.31	0.37	0.05371
924	511	20050821	22.48	140.30	6.91	42.00	2.39E-05	2.22	2.54E-06	-4.5648	4.58	0.30	0.06652
925	512	20050818	21.60	153.50	4.33	10.00	2.50E-06	3.05	-1.51E-06	-2.64316	3.47	0.22	0.03594
926	512	20050819	22.15	152.18	6.58	11.00	8.18E-06	2.55	-5.89E-07	-3.22352	4.75	0.26	0.04576
927	512	20050820	22.80	150.80	6.83	15.00	1.93E-05	2.16	-4.34E-06	-0.58467	4.80	0.27	0.04683
928	513	20050825	12.50	144.60	5.21	15.00	2.17E-05	3.06	1.46E-06	-14.0979	1.79	0.13	0.03231
929	513	20050826	12.83	143.28	8.07	15.00	4.00E-05	3.21	1.36E-06	-14.7641	1.69	0.08	0.03239
930	513	20050827	15.95	141.28	8.39	19.75	5.488E-05	7.15	2.04E-06	6.211524	2.15	0.21	0.0393
931	513	20050828	19.53	137.25	9.42	31.50	6.57E-05	6.64	2.75E-06	13.05887	2.86	0.27	0.05207
932	513	20050829	21.03	132.40	9.47	42.50	6.65E-05	5.47	1.54E-06	6.781891	2.45	0.30	0.05773
933	513	20050830	21.70	128.50	11.82	55.00	0.0001149	5.95	2.11E-06	-0.19862	3.33	0.21	0.06945
934	514	20050829	14.90	152.73	7.04	18.25	5.30E-05	4.84	5.26E-07	-13.4483	2.42	0.13	0.03758
935	514	20050830	15.05	148.60	6.58	29.50	3.88E-05	5.09	1.15E-06	-16.5889	2.09	0.27	0.04678
936	514	20050831	16.35	144.40	11.78	42.50	0.0002227	6.48	1.68E-06	-14.1479	2.14	0.23	0.06213
937	514	20050901	18.03	140.13	12.97	52.50	0.0001947	5.49	3.50E-06	-21.9241	3.14	0.32	0.0769
938	514	20050902	19.93	136.43	13.04	57.50	0.0001773	4.61	3.35E-06	-20.5756	3.17	0.35	0.08027
939	514	20050903	21.85	134.20	13.12	55.00	0.0001641	5.43	3.49E-07	-5.45916	3.81	0.37	0.0786
940	515	20050905	9.05	140.48	5.67	12.75	3.02E-05	6.45	-4.13E-07	1.104556	1.86	0.37	0.03602
941	515	20050906	11.68	136.50	6.46	15.75	2.10E-05	5.73	-2.21E-06	10.58102	1.88	0.20	0.03112
942	515	20050907	14.05	133.75	7.20	22.00	4.23E-05	3.44	-2.62E-07	-24.736	1.97	0.13	0.03842
943	515	20050908	16.80	131.83	6.56	31.00	3.41E-05	5.66	2.23E-06	-1.83138	2.44	0.21	0.04634
944	515	20050909	20.80	129.00	7.35	40.00	3.75E-05	6.81	-9.40E-07	22.87973	2.90	0.13	0.04741
945	517	20050919	20.35	154.05	4.76	10.00	1.12E-05	3.75	1.59E-06	-8.12131	3.22	0.22	0.03742
946	517	20050920	21.78	151.75	6.38	15.00	2.13E-05	2.74	3.30E-06	0.8034	4.80	0.31	0.05214
947	517	20050921	22.60	150.60	10.60	20.00	4.92E-05	2.84	1.69E-06	4.126887	4.67	0.28	0.05539
948	519	20050925	19.03	144.67	6.83	13.00	1.97E-05	3.22	2.28E-06	2.547448	3.18	0.29	0.04241
949	519	20050926	20.20	142.68	8.61	22.25	1.71E-05	3.92	-3.03E-06	2.226368	4.26	0.32	0.05117
950	519	20050927	21.83	139.68	8.87	38.75	3.49E-05	4.32	-1.77E-06	9.304172	4.19	0.23	0.0579
951	519	20050928	22.38	136.48	7.92	48.75	3.21E-05	2.75	1.31E-06	5.238746	3.88	0.27	0.06583
952	519	20050929	22.33	134.20	7.11	50.00	3.51E-05	3.74	-1.70E-06	0.866546	3.91	0.21	0.06224
953	519	20050930	22.15	130.40	9.32	46.25	5.55E-05	6.14	7.00E-07	-3.28922	3.25	0.17	0.05876
954	519	20051001	22.50	127.00	9.79	55.00	5.44E-05	7.17	-1.58E-06	5.408613	3.43	0.28	0.06599
955	520	20051009	22.53	136.73	8.25	12.00	1.70E-05	4.33	4.71E-06	-1.77044	3.79	0.23	0.04481
956	520	20051010	22.40	133.83	6.39	17.75	1.79E-05	2.65	-3.09E-06	-5.30789	3.74	0.23	0.04312
957	520	20051011	20.90	132.90	6.47	35.00	2.86E-05	1.49	-7.77E-07	0.318961	2.62	0.39	0.05504
958	520	20051012	20.78	132.20	8.36	48.75	4.86E-05	1.53	1.66E-06	-1.86343	2.37	0.34	0.06342
959	520	20051013	21.75	131.78	6.71	45.00	3.25E-05	1.75	4.70E-06	-4.09711	3.01	0.37	0.06529
960	520	20051014	22.83	131.27	6.56	45.00	2.90E-05	1.26	4.57E-06	-8.12873	3.45	0.34	0.06653
961	522	20051107	12.03	137.67	7.69	12.00	1.08E-05	4.72	-1.64E-06	13.22177	1.87	0.27	0.03231
962	522	20051108	13.23	134.28	9.39	12.00	1.48E-05	6.01	3.21E-07	15.95175	1.98	0.22	0.03251
963	522	20051109	13.37	129.70	9.71	15.00	2.67E-05	9.21	3.92E-08	11.15992	1.43	0.20	0.03058
964	523	20051114	9.80	128.85	7.13	15.00	3.31E-05	4.33	6.42E-07	1.591625	1.30	0.36	0.03697
965	523	20051115	12.00	129.93	6.88	15.00	5.66E-05	4.00	-3.67E-07	0.737022	1.48	0.17	0.03188
966	523	20051116	13.68	129.60	7.58	20.25	4.43E-05	1.38	1.64E-06	2.38002	1.40	0.14	0.03569
967	523	20051117	13.28	128.63	10.53	30.50	7.49E-05	2.02	1.59E-06	9.590548	1.18	0.32	0.04836
968	523	20051118	14.33	127.87	9.29	30.00	7.13E-05	4.74	1.81E-06	13.94314	1.06	0.23	0.04252
969	601	20060508	8.60	134.57	4.29	15.00	3.19E-05	3.32	1.90E-06	-7.15274	1.37	0.28	0.03497
970	601	20060509	8.50	132.38	4.69	17.00	3.03E-05	3.51	8.59E-07	-2.30364	1.39	0.36	0.03791
971	601	20060510	9.73	129.75	7.97	24.75	5.20E-05	4.20	1.65E-06	-1.98879	1.41	0.43	0.04724
972	601	20060511	10.90	128.10	10.62	28.00	0.0001437	4.08	1.22E-06	14.79221	1.38	0.31	0.04852
973	603	20060629	6.03	139.50	5.71	13.00	4.85E-05	1.92	1.44E-06	-14.3399	1.78	0.16	0.03396
974	603	20060630	6.83	138.23	9.90	15.75	0.0001149	3.39	1.41E-06	-16.5732	1.63	0.12	0.03759
975	603	20060701	9.70	136.85	8.92	21.00	1.17E-04	3.99	3.42E-06	-5.4634	1.46	0.25	0.04386
976	603	20060702	11.48	134.98	6.98	25.75	4.88E-05	2.68	1.14E-06	-9.5124	1.77	0.24	0.04351
977	603	20060703	13.58	134.10	7.59	40.75	5.17E-05	4.29	2.43E-06	-16.1698	1.98	0.22	0.05335
978	603	20060704	16.40	132.30	8.49	48.75	6.58E-05	4.40	-9.75E-07	-11.6299	2.52	0.33	0.06226
979	603	20060705	18.35	129.95	9.69	47.50	7.78E-05	2.99	-8.01E-07	-6.39962	1.73	0.29	0.0583
980	603	20060706	19.85	128.33	10.28	45.00	7.06E-05	2.52	3.55E-06	-8.83315	2.32	0.44	0.06751
981	603	20060707	21.43	127.37	12.92	45.00	0.0001614	3.65	2.55E-06	-0.23021	3.30	0.47	0.07565
982	604	20060708	12.03	139.67	9.49	14.00	3.77E-05	4.90	3.33E-07	-13.6512	1.82	0.26	0.0378
983	604	20060709	13.80	136.43	9.57	17.25	8.81E-05	6.12	1.97E-06	-18.9615	1.86	0.18	0.03961
984	604	20060710	16.53	132.23	9.15	21.50	5.63E-05	6.05	-2.05E-06	-19.1835	2.50	0.26	0.04376
985	604	20060711	19.23	128.58	11.16	28.00	1.04E-04	5.78	6.04E-06	-13.2332	1.70	0.54	0.06032
986	605	20060717	8.90	147.80	5.47	12.00	1.97E-05	4.81	1.20E-06	-3.90727	0.89	0.30	0.03064
987	605	20060718	9.95	145.20	5.82	14.25	2.48E-05	6.70	4.11E-07	-5.15053	1.28	0.41	0.03634
988	605	20060719	11.70	140.40	7.53	17.75	2.49E-05	5.09	3.10E-07	1.937343	1.86	0.49	0.04464
989	605	20060720	13.73	137.03	7.13	29.00	5.24E-05	6.58	-3.91E-07	-4.23948	1.84	0.17	0.04066
990	605	20060721	15.93	132.35	7.77	37.50	4.70E-05	5.67	2.66E-07	-9.47663	2.52	0.34	0.05539
991	605	20060722	17.13	128.90	9.03	40.00	4.15E-05	3.80	-1.22E-06	-10.0197	1.09	0.28	0.04893
992	605	20060723	18.50	127.40	7.18	40.00	4.31E-05	4.60	1.70E-06	-16.6995	1.45	0.45	0.05705
993	606	20060728	13.47	129.20	6.12	12.00	1.76E-05	1.41	2.60E-06	0.455128	1.26	0.27	0.03355
994	606	20060729	13.75	128.13	5.71	12.00	3.29E-05	2.23	7.44E-07	-13.8955	1.07	0.24	0.0317
995	606	20060730	14.10	127.00	6.71	12.00	4.11E-05	2.70	-4.80E-07	8.948282	1.23	0.15	0.0277
996	608	20060805	11.50	146.95	5.08	17.00	2.73E-05	7.48	-1.61E-07	-15.183	1.04	0.19	0.0294
997	608	20060806	15.68	142.33	5.45	22.75	3.14E-05	7.69	-1.48E-06	-7.79441	2.03	0.21	0.03659
998	608	20060807	19.15	137.45	5.21	33.75	1.87E-05	7.62	8.01E-07	-5.82077	2.75	0.28	0.04887
999	608	20060808	21.63	132.83	6.08	38.33	2.65E-05	8.74	2.25E-06	-3.78594	2.94	0.18	0.05027

1000	609	20060805	20.73	132.43	5.71	15.00	7.01E-06	2.50	-1.57E-07	1.489102	2.38	0.17	0.03429
1001	609	20060806	22.10	130.73	5.00	17.75	8.43E-06	3.89	-2.42E-06	-1.59348	3.30	0.28	0.04134
1002	609	20060807	22.87	127.87	5.91	24.33	1.77E-05	4.18	-3.11E-06	-0.83343	3.32	0.43	0.05024
1003	611	20060813	17.35	128.30	7.43	15.00	3.58E-05	6.22	-2.65E-06	-6.22662	1.21	0.22	0.03014
1004	611	20060814	19.15	131.75	7.08	18.50	2.91E-05	7.88	2.28E-06	-4.856	2.26	0.29	0.04061
1005	611	20060815	21.75	136.35	9.73	17.50	1.85E-05	9.90	-1.29E-06	3.764713	3.83	0.27	0.04405
1006	613	20060909	14.05	137.78	6.14	15.00	2.18E-05	4.42	-2.26E-08	2.246501	1.94	0.17	0.03232
1007	613	20060910	16.58	135.20	6.96	17.00	2.44E-05	4.31	1.97E-07	-3.2924	1.44	0.23	0.03413
1008	613	20060911	18.68	133.38	5.87	30.25	2.00E-05	2.95	-2.21E-06	0.778328	2.10	0.48	0.05098
1009	613	20060912	19.83	131.18	7.02	40.00	2.61E-05	4.02	3.52E-06	1.360352	2.44	0.18	0.05185
1010	613	20060913	20.23	128.30	7.73	40.00	5.28E-05	3.41	-2.88E-07	-11.5377	2.74	0.18	0.05322
1011	614	20060916	19.90	156.40	9.45	15.00	3.02E-05	2.67	-2.73E-06	-8.70864	2.89	0.26	0.04131
1012	614	20060917	20.85	157.78	9.04	18.25	3.42E-06	2.54	-7.26E-08	8.407426	3.07	0.14	0.03952
1013	614	20060918	20.50	159.15	6.56	25.25	7.07E-06	1.53	-5.57E-07	0.859658	2.94	0.23	0.04502
1014	614	20060919	19.98	158.05	5.73	39.50	1.88E-05	4.17	1.54E-07	-7.07092	2.96	0.22	0.05265
1015	614	20060920	19.63	153.25	7.37	48.75	3.25E-05	7.76	1.72E-06	5.313781	3.26	0.22	0.05933
1016	614	20060921	21.23	147.00	7.48	56.25	2.96E-05	8.53	3.25E-06	3.040915	4.22	0.26	0.06955
1017	616	20060925	11.80	128.60	5.94	14.25	4.28E-05	1.89	2.35E-08	-0.60004	1.39	0.36	0.03733
1018	616	20060926	11.85	127.40	6.57	20.50	4.57E-05	2.08	2.07E-06	3.696131	1.35	0.29	0.03974
1019	617	20060928	12.75	143.25	4.01	12.00	6.06E-06	2.74	-1.60E-06	-1.6646	1.69	0.28	0.03128
1020	617	20060929	12.08	141.93	5.10	12.00	1.39E-05	2.90	-3.23E-07	-6.79567	1.69	0.41	0.03759
1021	617	20060930	12.38	139.15	4.58	12.75	1.92E-05	5.24	1.87E-06	-7.33981	1.82	0.22	0.03235
1022	617	20061001	13.75	134.88	4.63	15.00	2.23E-05	4.93	-9.09E-07	-10.5391	1.94	0.13	0.03007
1023	617	20061002	14.58	131.88	5.22	15.00	2.45E-05	2.81	3.00E-06	-5.625	2.25	0.26	0.03898
1024	617	20061003	16.68	130.83	6.52	18.50	3.35E-05	4.77	-2.12E-09	-3.11305	1.94	0.19	0.03565
1025	617	20061004	20.10	132.40	8.61	20.75	3.93E-05	6.31	2.44E-06	-5.25623	2.38	0.46	0.05004
1026	618	20061002	18.10	151.75	7.34	15.00	4.04E-05	8.98	1.29E-06	-13.0093	2.89	0.38	0.04429
1027	618	20061003	20.43	153.90	6.96	17.75	3.34E-05	3.14	-1.15E-06	-1.55745	3.27	0.30	0.04472
1028	618	20061004	22.00	152.43	7.09	20.00	3.68E-05	1.80	1.55E-06	-8.31935	4.53	0.23	0.05193
1029	618	20061005	22.60	151.90	7.30	23.00	3.10E-05	1.40	-4.72E-07	2.287564	4.94	0.21	0.05299
1030	619	20061008	13.80	159.20	4.04	15.00	1.27E-05	7.47	4.33E-07	0.258825	2.84	0.26	0.03659
1031	619	20061009	15.83	155.85	6.72	16.50	3.03E-05	6.27	-1.72E-07	-4.9529	2.71	0.16	0.03627
1032	619	20061010	18.58	151.25	9.20	21.50	5.70E-05	8.97	2.47E-06	1.861121	3.09	0.25	0.04595
1033	619	20061011	20.68	144.48	7.67	28.25	4.26E-05	6.23	2.25E-06	-0.33009	4.25	0.17	0.05214
1034	619	20061012	22.20	141.55	6.47	30.00	2.85E-05	4.75	-7.07E-07	1.63931	4.63	0.14	0.05132
1035	620	20061025	12.13	140.27	5.32	12.00	1.12E-05	6.43	-6.81E-07	-0.06702	1.90	0.30	0.03261
1036	620	20061026	11.98	135.65	4.08	12.75	1.63E-05	7.14	4.10E-07	-2.66957	1.82	0.24	0.03078
1037	620	20061027	13.20	130.60	3.37	20.25	1.32E-05	5.68	-4.94E-07	4.768517	1.70	0.19	0.03184
1038	620	20061028	14.60	127.65	4.94	32.50	2.35E-05	5.44	1.88E-06	4.21272	1.09	0.27	0.04177
1039	621	20061108	15.60	135.57	8.22	15.00	9.54E-06	6.97	1.46E-07	7.213177	1.60	0.20	0.03115
1040	621	20061109	15.93	130.68	7.31	17.75	1.62E-05	6.84	-4.68E-07	20.34514	1.97	0.24	0.0341
1041	621	20061110	16.10	127.20	8.31	28.00	2.62E-05	6.93	3.56E-08	1.249087	1.15	0.16	0.03643
1042	622	20061125	9.57	146.13	9.38	15.00	6.49E-06	5.51	6.36E-07	0.951849	1.02	0.43	0.03818
1043	622	20061126	10.20	142.23	9.87	18.25	1.93E-05	5.53	1.18E-07	2.050462	1.77	0.36	0.04113
1044	622	20061127	10.78	138.15	10.62	22.75	5.48E-05	6.39	1.01E-06	5.235302	1.92	0.49	0.05019
1045	622	20061128	12.00	132.38	11.15	30.75	7.25E-05	8.00	-5.09E-07	2.810246	1.80	0.47	0.05312
1046	622	20061129	13.10	127.95	9.18	47.50	4.34E-05	6.61	6.73E-07	18.21971	1.20	0.26	0.05139
1047	623	20061207	9.45	135.05	7.63	15.75	3.47E-05	6.54	4.50E-07	19.50562	1.46	0.34	0.03547
1048	623	20061208	10.63	129.65	7.74	24.75	2.48E-05	7.19	1.96E-06	11.32602	1.42	0.34	0.04132
1049	624	20061215	8.43	154.50	8.04	15.00	1.80E-05	12.46	-4.04E-07	-0.90437	0.74	0.13	0.02345
1050	624	20061216	9.70	146.88	8.75	15.00	1.83E-05	10.82	1.34E-06	7.683671	0.95	0.32	0.03201
1051	624	20061217	12.65	139.25	8.37	16.50	1.77E-05	8.99	-1.04E-07	7.410149	1.88	0.28	0.0351
1052	624	20061218	15.23	134.35	8.55	17.25	2.26E-05	8.39	1.61E-07	11.76473	1.77	0.14	0.03092
1053	624	20061219	13.20	129.03	7.91	12.00	2.37E-05	7.00	-9.07E-07	8.647706	1.26	0.21	0.02777
1054	701	20070330	6.05	158.15	5.11	13.50	2.25E-05	2.45	1.24E-07	0.221565	0.46	0.17	0.02526
1055	701	20070331	7.45	156.05	7.03	15.00	7.306E-05	7.67	2.93E-07	9.92249	0.63	0.17	0.02709
1056	701	20070401	10.33	151.78	8.49	21.50	6.97E-05	5.17	4.01E-06	24.87709	0.96	0.39	0.04222
1057	701	20070402	13.80	148.25	9.71	31.00	7.13E-05	7.07	7.22E-07	44.01176	2.03	0.17	0.04199
1058	701	20070403	17.35	144.78	9.92	37.50	7.49E-05	5.79	1.10E-06	14.44967	2.67	0.25	0.05389
1059	701	20070404	21.45	145.98	10.97	32.50	5.34E-05	7.84	2.58E-06	-17.5677	4.14	0.26	0.0598
1060	702	20070515	8.23	146.07	6.11	12.00	1.31E-05	3.57	-3.28E-07	-4.11232	1.01	0.19	0.02735
1061	702	20070516	8.93	143.48	7.85	14.25	1.54E-05	5.13	4.31E-07	-3.83813	1.51	0.17	0.03121
1062	702	20070517	9.55	138.73	4.56	19.00	2.42E-05	6.89	1.41E-07	-2.20215	1.75	0.35	0.03809
1063	702	20070518	12.78	134.30	6.65	28.50	1.30E-05	6.78	-8.38E-07	15.70909	1.94	0.28	0.04091
1064	702	20070519	16.53	132.40	4.59	40.00	9.93E-06	4.75	-1.21E-07	2.142124	2.46	0.24	0.04934
1065	702	20070520	19.48	134.88	8.46	47.50	2.91E-05	6.70	-4.53E-06	12.84294	2.96	0.27	0.05554
1066	702	20070521	22.30	138.40	11.69	45.00	9.68E-06	9.08	1.79E-06	2.585331	3.92	0.30	0.0639
1067	704	20070707	6.00	148.43	7.26	15.00	9.21E-05	4.01	1.60E-06	-6.91092	0.19	0.31	0.03396
1068	704	20070708	8.18	145.40	5.08	15.75	2.99E-05	7.01	-8.02E-07	-6.11907	1.13	0.17	0.02776
1069	704	20070709	11.15	140.88	7.66	23.25	7.48E-05	6.04	9.20E-07	-21.7654	1.80	0.18	0.04073
1070	704	20070710	13.43	136.83	11.87	32.25	1.72E-04	7.42	2.54E-07	-15.9608	1.84	0.11	0.04818
1071	704	20070711	18.05	132.15	9.43	43.75	7.55E-05	8.73	2.62E-06	-17.7052	1.94	0.31	0.05806
1072	704	20070712	21.60	128.90	9.66	52.50	6.87E-05	6.79	2.46E-06	-11.3411	3.28	0.49	0.07473
1073	705	20070727	18.60	154.30	4.90	12.00	9.25E-06	11.51	-4.37E-06	-10.4009	2.69	0.27	0.03121
1074	705	20070728	17.78	149.50	3.99	15.00	7.60E-06	5.36	-3.11E-06	-10.1306	2.48	0.20	0.03233
1075	705	20070729	18.40	144.55	6.10	22.25	2.24E-05	5.38	-3.77E-06	-17.5725	2.95	0.22	0.04114
1076	705	20070730	20.23	141.65	5.85	33.25	2.31E-05	4.43	4.24E-06	-10.8677	4.10	0.22	0.05675
1077	705	20070731	22.25	139.90	7.31	42.50	3.60E-05	4.06	-3.16E-06	-4.92899	4.43	0.30	0.06294
1078	707	20070804	18.40	137.50	8.94	15.00	-2.69E-07	5.09	2.18E-06	0.275175	2.53	0.23	0.03874
1079	707	20070805	19.73	134.88	7.99	18.25	1.35E-05	6.61	3.05E-06	-5.9217	3.18	0.36	0.04798
1080	707	20070806	21.43	129.48	10.09	23.25	1.13E-05	6.89	-3.94E-06	-11.5894	3.14	0.44	0.05043
1081	708	20070806	15.00	129.35	7.68	15.00	3.15E-05	5.93	3.92E-07	-9.05484	1.38	0.25	0.03389
1082	708	20070807	16.43	127.40	9.57	15.00	2.49E-05	5.67	-3.79E-06	-14.4338	1.18	0.27	0.0326
1083	709	20070812	17.30	135.50	8.33	15.00	1.39E-05	1.93	-1.79E-07	2.250486	1.37	0.26	0.03481
1084	709	20070813	16.63	133.45	6.60	25.75	2.84E-05	3.68	-2.19E-06	-6.59248	1.99	0.20	0.03973
1085	709	20070814	16.03	130.40	8.21	40.00	4.56E-05	3.78	-6.06E-07	-12.6567	1.77	0.29	0.05262
1086	709	20070815	16.00	127.93	9.70	52.50	8.68E-05	3.56	4.63E-06	-5.28555	1.11	0.43	0.06641
1087	710	20070827	16.70	152.50	7.25	12.00	9.07E-06	4.65	1.56E-06	-4.79853	2.49	0.18	0.0346
1088	710	20070828	18.73	154.33	7.76	14.25	2.70E-05	5.73	3.06E-06	-11.6792	2.70	0.27	0.04182
1089	710	20070829	21.70	156.23	9.17	21.00	5.13E-05	4.56	1.55E-06	-11.2079	2.99	0.23	0.04655
1090	712	20070911	19.05	138.35	7.98	15.00	1.52E-05	5.85	3.48E-06	-11.7547	2.78	0.33	0.04439
1091	712	20070912	20.75	135.98	7.30	15.00	1.86E-05	4.20	-7.73E-07	-15.6067	3.68	0.26	0.04427
1092	712	20070913	22.30	133.55	6.87	19.00	2.15E-05	11.91	1.40E-06	-3.42433	3.57	0.15	0.03945
1093	713	20070915	19.68	132.63	6.77	15.00	2.10E-05	3.16	5.39E-06	-6.05602	2.45	0.32	0.04409
1094	713	20070916	21.05	130.13	9.70	24.75	6.62E-05	5.03	4.10E-07	-14.8076	2.95	0.36	0.05319
1095	713	20070917	22.40	127.70	8.37	35.00	5.26E-05	5.20	2.29E-06	-7.37425	3.32	0.36	0.05963
1096	716	20071001	17.30	130.77	8.23	16.00	4.72E-05	1.79	-3.84E-06	-0.00848	1.81	0.27	0.03643
1097	716	20071002	16.80	131.18	7.47	27.25	4.65E-05	2.10	-1.38E-06	-13.8149	2.12	0.28	0.04641
1098	716	20071003	17.55	129.58	8.68	40.00	6.66E-05	3.57	-7.83E-07	-15.3069	1.26	0.34	0.05317
1099	716	20071004	18.90	127.70	10.57	47.50	0.0001059	4.24	5.55E-06	-14.2629	1.60	0.26	0.06208

1100	720	20071018	17.95	145.40	4.95	15.00	1.50E-05	5.31	2.97E-07	-15.432	2.94	0.18	0.03692
1101	720	20071019	20.20	142.55	6.89	23.25	2.53E-05	5.89	1.55E-07	1.06285	4.26	0.28	0.05053
1102	720	20071020	22.10	140.70	5.69	40.00	1.74E-05	3.39	-7.68E-07	5.892828	4.48	0.18	0.05704
1103	722	20071101	18.10	132.50	6.06	12.00	1.41E-05	1.56	-6.43E-07	-16.1128	1.86	0.19	0.03258
1104	722	20071102	18.25	131.15	5.63	12.75	1.08E-05	3.81	-4.14E-07	-2.65842	2.11	0.20	0.03249
1105	722	20071103	18.10	128.23	7.98	16.00	1.72E-05	5.09	-1.19E-06	5.063106	1.31	0.29	0.03357
1106	724	20071120	12.18	137.00	7.44	16.50	3.77E-05	7.94	-1.82E-06	10.11256	1.86	0.32	0.0358
1107	724	20071121	14.23	132.23	10.47	25.25	6.65E-05	5.30	4.38E-07	-3.33081	2.10	0.19	0.04374
1108	724	20071122	14.33	128.60	14.16	37.67	1.10E-04	4.57	3.02E-06	-7.03383	1.13	0.38	0.05835
1109	724	20071128	20.00	127.50	8.20	15.00	7.55E-06	4.61	9.65E-07	-32.9712	2.51	0.29	0.04217
1110	802	20080507	7.88	132.28	6.59	15.75	4.32E-05	3.07	3.34E-06	-13.7307	1.37	0.24	0.0372
1111	802	20080508	9.35	131.40	9.37	21.50	1.07E-04	3.41	4.35E-06	-17.408	1.52	0.47	0.05295
1112	802	20080509	12.35	132.10	11.67	36.50	0.0001954	4.36	1.83E-08	-21.5158	1.80	0.17	0.0547
1113	802	20080510	16.23	132.00	12.61	52.50	0.0001737	6.30	3.55E-06	-3.65281	2.60	0.36	0.07335
1114	802	20080511	20.70	132.20	14.90	50.00	0.0002126	7.11	5.85E-06	-0.94629	2.33	0.61	0.08219
1115	805	20080526	11.67	139.33	5.37	13.00	1.12E-05	6.25	2.38E-07	1.594539	1.73	0.28	0.03242
1116	805	20080527	14.15	137.60	4.56	19.00	1.39E-05	1.93	-6.37E-07	-17.0559	1.93	0.14	0.03422
1117	805	20080528	15.55	136.83	4.98	29.50	1.86E-05	1.94	7.44E-07	-19.2831	1.86	0.21	0.04346
1118	805	20080529	16.23	135.68	5.86	46.25	2.73E-05	2.12	8.02E-07	-20.0895	1.51	0.24	0.05325
1119	805	20080530	17.20	133.83	5.64	47.50	2.82E-05	3.24	-3.18E-06	-12.0026	1.76	0.32	0.05436
1120	805	20080531	19.35	132.85	8.95	35.75	8.17E-05	2.97	8.07E-06	-2.54374	2.37	0.40	0.0628
1121	805	20080601	21.50	133.10	10.10	34.33	7.45E-05	4.71	-4.09E-06	1.693014	2.95	0.39	0.05653
1122	806	20080617	8.40	134.20	3.40	12.00	2.22E-05	4.36	1.83E-06	-19.89	1.40	0.27	0.03255
1123	806	20080618	9.15	132.48	3.84	13.50	2.53E-05	3.37	2.15E-06	-29.6411	1.45	0.41	0.03967
1124	806	20080619	10.28	129.38	6.81	24.75	5.67E-05	6.17	-6.13E-08	-23.8354	1.39	0.45	0.04727
1125	808	20080724	21.83	132.48	4.88	14.25	1.47E-05	2.23	4.45E-09	-5.72116	2.93	0.30	0.04085
1126	808	20080725	21.70	130.15	6.62	19.00	2.99E-05	4.29	8.03E-07	-15.3112	3.19	0.45	0.05136
1127	808	20080726	21.50	127.50	10.08	26.50	7.80E-05	3.97	3.55E-06	-9.19755	3.31	0.30	0.05604
1128	812	20080817	15.80	136.87	7.59	16.00	4.79E-05	8.94	-1.61E-06	-1.30154	1.86	0.27	0.035
1129	812	20080818	16.23	130.93	9.23	25.50	8.34E-05	8.46	3.70E-06	13.97811	2.12	0.35	0.04845
1130	814	20080917	15.85	140.65	4.00	12.00	8.55E-06	5.27	1.99E-06	-15.849	2.07	0.13	0.03026
1131	814	20080918	15.28	137.83	4.75	12.00	1.91E-05	5.06	2.14E-06	-18.2809	2.14	0.17	0.03295
1132	814	20080919	14.00	133.43	5.40	17.75	2.70E-05	5.82	-9.35E-07	-15.9396	1.98	0.14	0.03292
1133	814	20080920	14.40	129.43	7.44	27.25	4.27E-05	5.71	1.92E-07	-24.6437	1.35	0.14	0.03852
1134	814	20080921	16.40	127.30	11.49	30.00	0.0001594	6.42	2.45E-06	-5.86766	1.19	0.27	0.04984
1135	815	20080923	11.60	138.60	4.27	15.00	1.96E-05	4.32	1.80E-06	-11.02	1.76	0.24	0.0346
1136	815	20080924	12.45	136.65	6.41	17.75	3.92E-05	5.30	3.01E-07	-17.0059	1.84	0.22	0.03701
1137	815	20080925	14.53	132.00	9.45	34.00	9.49E-05	6.31	1.18E-06	-9.03882	2.22	0.32	0.05415
1138	815	20080926	17.30	128.48	11.96	48.75	0.0001525	6.23	-6.39E-07	14.51325	1.15	0.26	0.05668
1139	817	20080928	8.00	134.30	4.16	13.00	1.56E-05	9.24	1.29E-06	-1.72627	1.40	0.18	0.02687
1140	817	20080929	8.93	130.18	6.71	14.00	2.95E-05	8.88	1.17E-06	17.65808	1.30	0.37	0.03365
1141	822	20081211	12.80	144.40	7.29	15.00	3.13E-05	7.74	1.72E-09	12.60736	1.79	0.27	0.03389
1142	822	20081212	13.75	139.68	7.69	17.00	3.04E-05	5.30	8.40E-07	-13.9314	2.01	0.15	0.03543
1143	822	20081213	13.93	135.28	9.46	23.00	5.93E-05	5.31	4.23E-08	-17.1216	1.90	0.17	0.04086
1144	822	20081214	13.63	131.78	6.49	25.00	4.33E-05	3.15	1.45E-07	-7.84686	1.98	0.19	0.04113
1145	822	20081215	14.63	130.38	10.99	32.00	1.35E-04	2.58	2.71E-07	11.70486	1.85	0.26	0.05138
1146	822	20081216	16.73	130.83	8.46	31.50	4.05E-05	3.90	2.82E-07	-5.1553	1.93	0.13	0.04315
1147	822	20081217	19.73	133.40	9.12	23.25	4.38E-05	8.03	-1.68E-07	-11.0428	2.79	0.11	0.04032
1148	822	20081218	22.00	137.30	9.58	18.00	3.03E-05	10.64	6.90E-07	-8.69447	3.50	0.20	0.04264
1149	901	20090504	16.50	129.13	5.68	37.00	2.30E-05	7.06	1.03E-06	-8.51975	1.07	0.20	0.04177
1150	901	20090505	18.55	134.75	7.11	40.00	3.45E-05	7.64	-2.08E-06	-15.4009	2.07	0.22	0.04809
1151	901	20090506	21.57	139.80	10.10	39.33	3.01E-05	9.24	-1.86E-07	-4.37511	4.07	0.17	0.05631
1152	902	20090509	17.73	128.13	9.26	14.50	4.83E-05	3.38	3.99E-06	3.966612	1.29	0.26	0.03732
1153	902	20090510	19.98	127.83	7.43	13.00	3.36E-05	3.60	1.14E-06	11.24337	2.48	0.30	0.03909
1154	902	20090511	21.95	127.10	6.17	11.50	2.38E-05	3.59	4.88E-06	0.352076	3.42	0.25	0.04278
1155	908	20090803	20.50	134.67	11.82	16.00	4.94E-05	4.71	2.73E-06	-0.9682	3.57	0.33	0.05116
1156	908	20090804	22.20	134.88	5.56	21.50	2.18E-05	3.51	-1.20E-06	-1.14921	4.14	0.44	0.05374
1157	908	20090805	22.83	132.13	7.77	32.00	4.99E-05	5.46	-8.64E-07	-0.05484	3.44	0.30	0.05384
1158	910	20090816	11.50	160.00	4.69	13.00	1.69E-05	6.83	1.55E-06	-2.60262	2.55	0.22	0.0345
1159	910	20090817	13.55	158.60	5.73	15.25	1.23E-05	4.31	-1.17E-06	0.147294	2.82	0.18	0.03534
1160	910	20090818	16.38	157.43	7.49	22.75	8.37E-06	2.49	2.33E-06	4.84137	2.96	0.17	0.04319
1161	910	20090819	17.80	157.58	6.09	37.25	1.95E-05	1.82	-8.97E-07	-7.4365	2.99	0.19	0.05126
1162	910	20090820	18.93	157.15	6.66	48.00	2.13E-05	1.71	2.37E-06	0.456718	2.88	0.22	0.05947
1163	910	20090821	20.90	156.73	8.17	51.00	4.12E-05	4.50	-1.75E-06	8.665721	3.03	0.18	0.05806
1164	912	20090902	17.60	130.00	4.60	15.00	1.68E-05	5.50	5.09E-07	-2.72835	1.54	0.15	0.02912
1165	912	20090903	17.30	128.10	8.06	15.75	3.25E-05	3.90	2.43E-06	-11.9618	1.27	0.14	0.0329
1166	912	20090904	18.50	130.38	9.28	20.75	3.98E-05	5.78	2.13E-06	-5.0915	2.02	0.36	0.04577
1167	912	20090905	21.23	133.67	8.07	25.00	3.23E-05	8.47	1.30E-06	0.101463	3.22	0.27	0.04731
1168	914	20090912	14.85	152.28	5.93	14.75	1.30E-05	4.70	1.60E-06	-8.67128	2.42	0.14	0.03443
1169	914	20090913	15.75	149.20	7.90	26.50	3.54E-05	2.28	2.66E-06	-14.0327	2.25	0.21	0.04651
1170	914	20090914	16.05	147.63	10.09	42.50	4.38E-05	2.90	1.08E-07	-9.77599	2.34	0.12	0.0526
1171	914	20090915	17.70	145.38	11.17	61.25	0.0001177	4.38	-3.89E-07	-7.1843	2.84	0.21	0.07022
1172	914	20090916	19.10	142.33	7.52	65.00	6.068E-05	3.77	3.96E-07	1.850185	3.55	0.18	0.07067
1173	914	20090917	21.33	140.08	10.94	58.75	2.00E-04	4.83	2.31E-06	3.915483	3.97	0.19	0.07645
1174	917	20090927	9.50	145.50	4.62	15.00	2.58E-05	5.11	1.21E-06	-11.7416	1.16	0.30	0.0336
1175	917	20090928	9.20	142.75	6.35	15.00	3.54E-05	6.50	1.60E-06	0.718457	1.66	0.31	0.03647
1176	917	20090929	8.05	138.60	6.56	20.25	6.77E-05	4.26	2.28E-06	2.495524	1.54	0.18	0.03708
1177	917	20090930	10.15	134.68	6.96	37.00	4.81E-05	8.08	-4.75E-07	17.23951	1.64	0.30	0.04633
1178	917	20091001	12.83	129.23	7.42	51.25	4.03E-05	7.30	1.58E-06	3.973764	1.34	0.14	0.05007
1179	918	20090929	10.50	158.07	4.21	16.00	2.13E-05	7.34	1.44E-06	5.234179	1.97	0.13	0.02987
1180	918	20090930	12.53	154.58	5.22	24.00	2.61E-05	4.46	5.38E-07	-7.39226	2.15	0.18	0.03912
1181	918	20091001	13.90	152.28	6.29	39.00	3.42E-05	2.84	-1.44E-07	-16.2861	2.24	0.14	0.04855
1182	918	20091002	14.78	150.10	7.96	50.00	5.07E-05	4.42	1.82E-06	1.952973	2.24	0.18	0.05694
1183	918	20091003	16.20	145.93	10.98	51.25	0.0001187	7.17	2.23E-06	9.50498	2.37	0.34	0.06593
1184	918	20091004	17.25	140.05	9.17	63.75	6.49E-05	7.74	2.65E-06	9.613585	2.73	0.22	0.06793
1185	918	20091005	19.70	134.30	8.07	62.50	4.49E-05	7.83	4.85E-06	4.351007	3.14	0.38	0.07434
1186	918	20091006	22.50	131.70	8.59	55.00	4.29E-05	7.69	5.32E-07	9.956664	3.43	0.15	0.06141
1187	919	20091008	16.40	143.58	8.57	14.50	2.67E-05	4.43	2.85E-08	21.52774	2.09	0.15	0.03232
1188	919	20091009	19.53	142.68	8.93	17.25	1.77E-05	2.91	4.92E-07	19.71596	3.89	0.21	0.04445
1189	919	20091010	21.45	142.23	8.66	18.00	2.16E-05	2.42	-2.91E-06	19.10957	4.49	0.21	0.04586
1190	919	20091011	22.67	142.77	5.22	20.00	1.66E-05	2.84	1.71E-06	7.18748	5.05	0.26	0.05196
1191	920	20091014	10.75	147.75	5.63	15.00	1.88E-05	11.86	1.00E-06	-3.7633	0.86	0.27	0.02821
1192	920	20091015	11.98	141.48	6.68	17.00	4.76E-05	8.92	-1.07E-06	-13.3126	1.76	0.19	0.033
1193	920	20091016	13.93	135.35	8.17	28.50	8.65E-05	6.73	3.99E-08	-5.23795	1.89	0.31	0.04717
1194	920	20091017	15.88	132.48	7.30	42.00	4.47E-05	3.05	-1.24E-07	-1.28061	2.49	0.16	0.05189
1195	920	20091018	17.50	133.68	9.23	55.00	6.68E-05	2.74	4.00E-06	3.703421	1.84	0.36	0.06689
1196	920	20091019	19.15	132.80	9.73	56.25	9.45E-05	4.74	3.38E-06	4.017211	2.26	0.29	0.06726
1197	920	20091020	20.43	129.28	10.91	42.50	8.959E-05	4.43	2.37E-06	-0.13988	2.59	0.31	0.0618
1198	920	20091021	20.20	127.10	12.28	40.00	0.0001214	4.16	3.44E-06	-6.51379	2.62	0.27	0.06235
1199	921	20091025	11.80	152.50	7.15	13.00	9.32E-06	5.91	2.66E-07	-1.6246	1.78	0.32	0.03534

1200	921	20091026	12.93	149.20	7.70	14.00	1.23E-05	8.08	-4.00E-07	6.032439	2.17	0.24	0.03358
1201	921	20091027	15.15	142.85	8.56	21.00	2.10E-05	8.91	1.14E-06	7.62274	1.92	0.17	0.03537
1202	921	20091028	16.23	136.20	10.24	38.25	6.10E-05	7.82	4.16E-06	9.098066	1.61	0.32	0.05308
1203	921	20091029	15.95	129.80	9.38	40.00	3.91E-05	7.65	9.64E-08	15.99864	1.39	0.31	0.04884
1204	922	20091122	7.08	148.18	8.59	14.50	0.0001392	2.09	4.85E-06	-7.18112	0.49	0.26	0.03839
1205	922	20091123	8.48	147.28	9.46	17.75	1.06E-04	3.31	2.28E-06	-3.12232	0.88	0.38	0.04314
1206	922	20091124	9.18	145.15	7.94	33.25	7.94E-05	3.65	3.14E-06	0.03285	1.23	0.26	0.04785
1207	922	20091125	12.13	142.60	7.19	58.75	6.27E-05	6.24	9.78E-07	-6.22318	1.64	0.23	0.06017
1208	922	20091126	15.45	139.93	8.69	60.00	5.19E-05	3.69	1.49E-06	10.53471	1.98	0.24	0.06343
1209	922	20091127	17.93	139.10	9.49	56.25	4.33E-05	2.74	1.28E-06	19.04148	2.85	0.25	0.06501
1210	922	20091128	19.10	139.23	6.74	56.25	3.42E-05	0.70	2.85E-06	0.603747	3.12	0.25	0.06738
1211	922	20091129	19.43	139.35	8.63	51.25	6.44E-05	0.43	2.74E-06	-5.16298	3.18	0.34	0.07042
1212	922	20091130	19.80	138.90	9.91	42.00	5.75E-05	1.75	9.07E-07	13.74817	2.97	0.38	0.0632
1213	922	20091201	20.63	137.70	9.56	31.50	3.76E-05	1.98	-1.27E-06	16.59047	3.04	0.26	0.05102
1214	922	20091202	21.20	135.45	6.71	22.75	4.85E-06	3.65	2.74E-06	-9.28616	3.93	0.28	0.0514
1215	922	20091203	21.70	134.00	3.47	15.00	4.70E-06	1.77	9.17E-07	-37.1153	3.64	0.27	0.04524
1216	1001	20100322	8.40	141.70	11.42	15.00	4.18E-05	5.79	-4.69E-07	24.75364	1.66	0.54	0.04437
1217	1001	20100323	10.28	138.03	9.32	15.00	3.63E-05	5.92	1.28E-06	52.03937	1.87	0.37	0.03756
1218	1001	20100324	12.83	134.00	8.27	16.50	3.95E-05	5.84	-4.15E-08	29.50606	1.95	0.40	0.04006
1219	1001	20100325	15.85	132.18	8.01	18.00	1.43E-05	3.76	-1.47E-06	13.67319	2.57	0.18	0.03632
1220	1001	20100326	17.57	131.83	7.93	15.33	3.72E-05	1.51	8.07E-07	-17.5505	2.15	0.28	0.04156
1221	1002	20100711	13.80	132.20	6.27	14.00	1.80E-05	5.83	8.11E-07	-6.23722	2.00	0.19	0.03311
1222	1002	20100712	14.27	129.13	6.79	24.33	1.58E-05	7.18	-1.93E-07	-10.2142	1.26	0.13	0.03302
1223	1007	20100828	19.00	138.35	7.92	15.00	2.03E-05	8.13	-5.03E-07	-7.36361	2.77	0.24	0.03788
1224	1007	20100829	21.35	134.90	5.86	17.00	1.63E-05	5.63	3.36E-07	-4.8623	3.91	0.27	0.04485
1225	1007	20100830	22.80	132.40	4.95	23.00	1.48E-05	5.67	1.60E-06	-9.72592	3.44	0.23	0.04558
1226	1009	20100901	16.05	140.70	8.11	13.00	4.17E-05	9.65	1.03E-06	-6.41842	2.17	0.19	0.03339
1227	1009	20100902	20.05	136.68	9.94	15.00	3.74E-05	9.41	2.40E-06	2.111394	3.16	0.25	0.0421
1228	1009	20100903	22.60	132.90	7.09	15.00	1.63E-05	9.31	-5.81E-07	1.596394	3.45	0.21	0.03802
1229	1011	20100914	19.60	129.10	4.96	15.00	2.06E-05	3.56	-1.81E-06	-12.2384	1.87	0.32	0.03631
1230	1011	20100915	20.58	127.95	6.50	17.75	2.73E-05	2.13	-2.64E-06	-7.91309	3.02	0.32	0.04391
1231	1011	20100916	21.68	128.20	9.71	30.00	4.99E-05	1.95	-7.28E-07	-7.81322	3.36	0.31	0.0558
1232	1011	20100917	22.55	128.15	8.88	36.50	6.14E-05	2.10	1.55E-06	-6.96866	3.27	0.25	0.05822
1233	1012	20100920	18.97	145.90	7.31	14.33	1.41E-05	2.18	1.10E-06	-14.7617	3.30	0.20	0.0418
1234	1012	20100921	19.13	144.55	7.89	15.75	2.65E-05	2.85	-2.39E-07	-19.3933	3.23	0.17	0.0413
1235	1012	20100922	19.60	142.10	9.15	21.50	6.697E-05	2.67	1.78E-06	-8.57724	3.89	0.23	0.05206
1236	1012	20100923	21.38	140.98	12.28	33.25	0.0001381	5.01	8.17E-06	1.641165	4.24	0.42	0.07292
1237	1013	20101013	11.95	141.00	6.09	16.00	4.04E-05	1.85	5.15E-07	-3.54727	1.84	0.12	0.03309
1238	1013	20101014	12.90	138.88	10.69	24.50	1.00E-04	4.47	-1.59E-07	-6.7244	1.87	0.30	0.04743
1239	1013	20101015	15.38	135.93	12.27	35.75	0.0001077	6.64	-4.58E-07	6.071117	1.66	0.27	0.05102
1240	1013	20101016	18.18	130.90	11.31	43.75	8.13E-05	6.98	-2.69E-06	1.650437	2.03	0.23	0.05314
1241	1013	20101017	18.70	127.50	11.85	60.00	0.0001112	6.35	2.27E-06	-5.28123	1.50	0.25	0.06595
1242	1014	20101023	15.00	133.60	6.67	15.00	2.03E-05	2.54	1.86E-06	-17.8195	1.92	0.23	0.03762
1243	1014	20101024	15.83	132.20	6.74	17.00	2.49E-05	3.59	2.13E-06	-20.8445	2.55	0.25	0.04213
1244	1014	20101025	17.75	130.28	7.54	27.25	3.79E-05	4.02	-6.38E-07	-23.0789	1.74	0.17	0.04123
1245	1014	20101026	20.03	128.45	8.86	36.50	4.47E-05	2.33	5.26E-06	-6.38637	2.48	0.22	0.05538
1246	1014	20101027	21.63	127.90	11.80	45.00	0.0001326	4.02	1.95E-06	-11.9231	3.37	0.19	0.0655
1247	1102	20110519	8.30	141.50	4.68	15.00	1.70E-05	2.04	-1.12E-06	-4.07092	1.70	0.38	0.03766
1248	1102	20110520	8.38	140.25	5.83	15.00	3.14E-05	2.96	2.30E-06	-5.78733	1.82	0.43	0.0429
1249	1102	20110521	9.00	138.05	6.76	15.75	4.93E-05	2.80	3.84E-06	-0.59072	1.49	0.22	0.0367
1250	1102	20110522	10.08	136.00	10.64	23.25	6.26E-05	4.00	9.35E-07	-6.07395	1.50	0.38	0.04699
1251	1102	20110523	11.28	132.83	10.38	28.00	9.02E-05	4.01	4.43E-08	-11.6932	1.79	0.28	0.04866
1252	1102	20110524	12.18	129.85	6.86	31.50	3.35E-05	3.79	2.70E-07	-8.64638	1.49	0.21	0.04304
1253	1102	20110525	12.95	127.68	8.21	45.00	1.09E-04	3.32	7.62E-07	-11.1385	1.27	0.14	0.05124
1254	1104	20110616	9.10	129.40	8.82	13.00	4.05E-05	4.85	3.45E-06	-2.26717	1.19	0.24	0.03442
1255	1104	20110617	11.28	128.23	6.39	13.50	2.92E-05	5.04	1.51E-06	1.49599	1.40	0.30	0.03437
1256	1105	20110620	11.20	133.20	5.85	15.00	1.75E-05	3.86	-1.27E-08	-4.6395	1.82	0.32	0.03703
1257	1105	20110621	12.03	131.10	7.07	15.00	3.78E-05	4.66	4.26E-07	-10.5382	1.56	0.34	0.03819
1258	1105	20110622	13.90	128.53	9.27	19.75	7.387E-05	4.15	2.10E-06	-22.9943	1.11	0.21	0.03927
1259	1105	20110623	16.20	127.15	9.63	25.00	5.89E-05	5.33	2.47E-06	-17.1526	1.17	0.43	0.04866
1260	1106	20110711	18.50	156.95	9.82	15.00	3.70E-05	3.81	2.70E-06	-8.65556	2.83	0.36	0.04741
1261	1106	20110712	19.08	154.68	8.85	19.75	3.34E-05	3.93	1.60E-07	-7.14509	2.70	0.29	0.04496
1262	1106	20110713	19.75	150.65	6.87	30.25	2.20E-05	6.33	4.76E-06	-4.64085	3.29	0.30	0.05402
1263	1106	20110714	20.15	145.63	7.46	40.00	6.71E-05	6.07	-6.16E-07	-13.2138	3.67	0.24	0.05867
1264	1106	20110715	20.58	141.00	6.74	48.50	3.65E-05	4.62	-2.73E-06	-12.6591	4.01	0.22	0.06193
1265	1106	20110716	21.73	137.53	9.21	53.75	5.77E-05	5.20	2.55E-06	-5.89627	3.35	0.27	0.06831
1266	1107	20110713	14.80	133.50	5.66	12.00	2.13E-05	2.06	-5.17E-07	-5.81276	1.95	0.15	0.03073
1267	1107	20110714	14.45	132.53	7.04	12.75	3.45E-05	2.07	5.05E-07	-7.35252	2.10	0.18	0.0349
1268	1107	20110715	14.15	133.70	8.51	17.25	4.02E-05	3.28	6.68E-07	-12.2919	1.96	0.16	0.0372
1269	1109	20110727	10.73	139.73	6.20	15.00	3.17E-05	7.68	2.03E-06	-16.7453	1.78	0.25	0.03556
1270	1109	20110728	11.88	134.68	7.03	19.00	1.96E-05	3.40	-1.24E-07	-20.5899	1.84	0.16	0.03625
1271	1109	20110729	13.68	133.83	8.59	26.50	3.12E-05	3.89	1.32E-06	-17.7317	1.99	0.14	0.04228
1272	1109	20110730	16.25	133.05	7.22	50.00	4.17E-05	2.19	-2.40E-07	-12.156	2.20	0.16	0.05598
1273	1109	20110731	17.53	133.15	10.58	55.50	1.26E-04	3.16	1.34E-06	-9.57942	1.92	0.19	0.064
1274	1109	20110801	20.18	134.05	12.61	50.50	1.71E-04	4.09	3.59E-06	-12.7931	3.34	0.24	0.07296
1275	1109	20110802	22.35	134.10	9.19	50.00	6.21E-05	3.59	-4.00E-07	-9.98051	3.87	0.33	0.06971
1276	1111	20110821	12.60	128.47	4.36	10.00	1.20E-05	3.89	-1.14E-06	-11.1395	1.35	0.16	0.02567
1277	1111	20110822	14.45	127.60	5.23	11.75	1.55E-05	1.90	2.47E-06	-10.1765	1.10	0.16	0.02901
1278	1111	20110823	15.73	127.33	6.11	17.00	2.98E-05	1.32	2.33E-07	-6.31511	1.12	0.42	0.04011
1279	1111	20110824	16.10	127.10	9.08	23.00	7.96E-05	0.99	-1.38E-06	-10.7416	1.16	0.35	0.04434
1280	1112	20110823	15.30	142.80	6.89	13.00	2.21E-05	5.59	1.31E-06	-18.8868	1.94	0.11	0.03143
1281	1112	20110824	16.73	141.35	6.21	14.50	1.97E-05	3.64	-4.29E-07	-17.7519	2.49	0.19	0.0363
1282	1112	20110825	19.58	140.50	8.53	19.00	3.86E-05	3.25	-1.76E-07	-18.613	3.68	0.20	0.04673
1283	1112	20110826	21.70	140.10	9.68	23.00	5.22E-05	2.16	3.60E-06	0.180674	4.19	0.42	0.06085
1284	1112	20110827	22.58	139.58	10.53	24.00	1.02E-04	0.78	3.11E-06	2.613412	4.52	0.38	0.06415
1285	1112	20110828	22.90	139.60	9.60	25.00	7.11E-05	0.70	4.34E-06	-1.13703	4.74	0.48	0.06819
1286	1113	20110902	20.00	148.20	5.93	14.00	1.91E-05	2.14	1.64E-06	-6.53376	3.12	0.23	0.04098
1287	1113	20110903	20.90	149.33	7.52	16.00	2.11E-05	8.15	-2.54E-07	1.512945	3.44	0.39	0.04543
1288	1114	20110905	17.90	132.20	5.27	12.00	1.10E-05	4.96	4.52E-08	2.433798	1.93	0.19	0.03004
1289	1114	20110906	19.75	134.18	4.79	12.75	9.87E-06	4.61	1.79E-06	0.287128	3.15	0.34	0.04149
1290	1114	20110907	21.90	135.53	4.93	17.67	1.00E-05	4.27	-1.49E-06	-2.09338	4.05	0.24	0.04367
1291	1115	20110909	20.25	143.95	8.32	12.00	1.67E-05	3.64	-1.88E-06	-2.21742	4.20	0.21	0.04219
1292	1115	20110910	20.25	141.23	6.01	12.75	1.32E-05	5.83	-1.33E-07	-5.63117	3.98	0.23	0.04135
1293	1115	20110911	20.73	136.93	5.22	15.00	1.45E-05	3.55	2.21E-06	-4.50704	3.32	0.23	0.04139
1294	1115	20110912	21.85	136.98	6.15	15.00	1.28E-05	2.77	-1.07E-06	-4.43817	3.54	0.34	0.04478
1295	1115	20110913	22.70	138.15	8.48	16.50	1.39E-05	2.11	1.44E-06	-6.85527	3.99	0.36	0.05169
1296	1116	20110914	21.20	154.60	6.49	15.00	1.50E-05	2.81	-3.56E-07	-0.44162	3.13	0.28	0.04158
1297	1116	20110915	22.30	154.78	7.29	17.25	1.21E-05	3.71	3.09E-07	-0.51129	3.30	0.23	0.04229
1298	1116	20110916	22.95	152.35	6.76	20.00	2.56E-05	6.19	3.19E-07	10.63883	4.86	0.29	0.05095
1299	1117	20110923	13.23	138.30	5.80	13.50	1.80E-05	4.27	7.05E-07	-6.54453	1.87	0.18	0.03219

1300	1117	20110924	14.73	134.28	8.55	21.50	5.05E-05	7.42	2.31E-06	-2.55169	1.86	0.24	0.04057
1301	1117	20110925	14.57	129.37	10.47	29.33	0.0001837	5.84	2.19E-06	-14.9308	1.35	0.24	0.05
1302	1119	20110926	18.15	139.15	5.57	12.00	7.45E-06	3.54	-3.72E-07	-14.2515	2.90	0.20	0.03598
1303	1119	20110927	18.83	137.65	7.16	15.75	3.10E-05	1.83	4.05E-08	-13.8703	2.65	0.29	0.04287
1304	1119	20110928	18.88	135.90	6.74	24.00	2.97E-05	3.51	4.37E-06	-9.2851	2.33	0.28	0.0469
1305	1119	20110929	17.83	132.33	7.25	32.00	4.02E-05	6.56	1.25E-06	-5.46658	1.92	0.19	0.04398
1306	1119	20110930	17.80	128.15	10.31	39.00	4.69E-05	7.36	2.53E-06	15.79836	1.28	0.21	0.04699
1307	1120	20111009	7.77	134.40	5.61	12.00	3.01E-05	5.56	-1.12E-07	3.945762	1.41	0.27	0.03073
1308	1120	20111010	7.55	130.38	8.45	14.25	3.17E-05	4.71	5.18E-06	-2.01417	1.05	0.73	0.05114
1309	1120	20111011	8.35	128.30	7.68	16.50	6.96E-05	3.99	6.34E-06	5.895477	0.84	0.55	0.04673
1310	1121	20111213	6.00	143.33	7.40	14.33	2.47E-05	7.70	2.44E-06	-7.83436	0.58	0.44	0.03636
1311	1121	20111214	6.35	138.35	8.04	15.00	3.57E-05	8.18	3.91E-06	3.017942	1.70	0.32	0.03837
1312	1121	20111215	7.63	131.75	5.66	18.25	2.14E-05	7.97	4.94E-06	-3.11162	1.25	0.44	0.04137
1313	1121	20111216	7.80	128.10	5.06	23.00	2.52E-05	6.64	4.91E-06	-6.71036	0.82	0.32	0.03889
1314	1202	20120520	10.70	148.00	7.27	10.00	2.69E-06	3.86	-9.57E-07	-4.19375	0.89	0.17	0.02511
1315	1202	20120521	11.75	145.95	7.05	14.00	9.86E-06	5.26	-1.39E-07	-3.58974	1.30	0.15	0.02819
1316	1202	20120522	14.33	142.85	8.21	19.00	2.92E-05	5.21	-7.86E-07	-2.75965	1.83	0.18	0.03543
1317	1202	20120523	17.05	140.23	10.20	23.25	4.03E-05	4.12	-4.35E-07	0.152328	2.67	0.41	0.051
1318	1202	20120524	20.20	139.10	9.51	30.25	1.92E-05	4.35	3.23E-06	1.582618	3.14	0.37	0.05641
1319	1202	20120525	22.60	139.35	9.91	33.00	8.43E-05	4.34	1.21E-07	-0.07789	4.47	0.18	0.05825
1320	1204	20120610	8.00	148.40	3.98	13.00	7.79E-06	5.27	-1.69E-07	-1.91122	0.65	0.13	0.02244
1321	1204	20120611	9.25	145.95	5.45	13.50	1.94E-05	4.81	2.08E-06	2.894681	1.09	0.15	0.02758
1322	1204	20120612	9.60	142.40	5.71	15.00	2.60E-05	3.82	1.33E-06	-2.64547	1.72	0.22	0.03407
1323	1204	20120613	10.33	138.60	4.34	19.75	1.74E-05	7.25	1.41E-06	-13.3512	1.84	0.16	0.03362
1324	1204	20120614	10.70	133.45	4.13	28.25	9.83E-06	4.67	1.20E-06	-7.70857	1.75	0.30	0.04289
1325	1204	20120615	11.00	131.00	8.42	32.75	3.64E-05	3.85	-1.43E-06	-5.93051	1.47	0.16	0.04172
1326	1204	20120616	14.50	129.25	10.49	50.75	7.88E-05	6.49	2.15E-06	-8.86428	1.31	0.18	0.05583
1327	1204	20120617	19.03	127.23	13.88	56.25	0.0001817	6.43	-3.51E-06	-7.99972	1.59	0.32	0.0668
1328	1204	20120618	22.20	127.40	9.60	48.00	5.83E-05	7.43	1.87E-07	-5.9731	3.39	0.24	0.06205
1329	1206	20120625	11.90	134.30	4.75	12.75	9.66E-06	5.08	9.61E-07	-8.52664	1.85	0.18	0.03066
1330	1206	20120626	14.35	130.83	3.82	15.75	1.35E-05	6.37	-3.02E-06	-8.26967	2.01	0.19	0.03004
1331	1206	20120627	15.60	127.45	7.53	18.00	4.25E-05	3.95	2.77E-06	-1.68594	1.11	0.17	0.03394
1332	1207	20120714	19.20	143.70	7.47	15.00	9.16E-06	3.74	1.67E-06	-7.86991	3.41	0.34	0.04661
1333	1207	20120715	20.48	141.63	8.38	15.00	1.55E-05	7.59	4.21E-06	-4.99555	4.19	0.43	0.0532
1334	1207	20120716	23.00	138.00	6.87	15.00	2.30E-05	10.95	-2.77E-06	-4.23743	4.14	0.27	0.04167
1335	1209	20120726	10.60	131.50	7.51	15.00	4.04E-05	8.74	1.57E-06	-17.5952	1.56	0.30	0.03676
1336	1209	20120727	12.25	128.45	8.23	15.00	2.54E-05	5.87	1.67E-07	-15.0163	1.40	0.17	0.03187
1337	1211	20120801	22.40	146.20	8.36	15.00	2.82E-05	3.81	1.81E-07	-9.87401	4.52	0.19	0.04675
1338	1211	20120802	22.47	144.00	8.44	15.00	2.91E-05	4.83	-1.25E-06	-8.52484	5.26	0.22	0.04981
1339	1213	20120812	16.60	130.00	7.43	14.00	1.87E-05	4.82	-2.21E-07	-8.76644	1.42	0.26	0.03355
1340	1213	20120813	16.70	127.65	9.91	18.00	4.67E-05	2.47	1.02E-07	-19.0772	1.21	0.32	0.04118
1341	1215	20120819	15.33	142.03	6.94	15.00	2.18E-05	5.24	1.53E-07	-20.825	2.00	0.18	0.03458
1342	1215	20120820	17.70	141.33	5.64	19.00	2.16E-05	1.70	1.96E-06	-14.1169	2.96	0.34	0.04764
1343	1215	20120821	18.65	140.15	8.60	29.00	5.45E-05	3.00	4.27E-06	-10.0192	3.36	0.49	0.06312
1344	1215	20120822	19.45	137.68	8.41	36.50	4.08E-05	3.48	-1.67E-06	-20.813	2.80	0.18	0.05154
1345	1215	20120823	20.08	134.90	9.23	39.50	5.05E-05	3.57	-7.37E-07	-16.0959	3.44	0.25	0.05911
1346	1215	20120824	21.95	133.13	11.09	45.00	0.0001183	3.47	-2.06E-07	-11.6961	3.24	0.21	0.06353
1347	1216	20120910	9.03	134.68	5.37	14.00	1.64E-05	2.08	1.14E-06	-17.1407	1.41	0.18	0.03193
1348	1216	20120911	11.00	133.53	4.98	18.50	2.61E-05	4.83	5.60E-07	-18.4169	1.80	0.22	0.03646
1349	1216	20120912	13.43	130.85	5.02	29.75	3.18E-05	3.64	1.47E-06	-24.3359	1.80	0.20	0.04362
1350	1216	20120913	15.95	129.70	6.87	55.00	5.68E-05	4.17	3.83E-06	-10.664	1.34	0.22	0.05897
1351	1216	20120914	19.68	129.70	10.39	58.75	1.02E-04	5.65	1.92E-06	-0.63209	2.11	0.31	0.06821
1352	1216	20120915	22.30	129.10	7.77	55.00	3.71E-05	5.00	1.73E-06	3.881043	3.01	0.14	0.06067
1353	1217	20120919	14.10	135.90	5.04	15.00	1.16E-05	6.68	-1.32E-06	-13.8668	1.85	0.21	0.03162
1354	1217	20120920	13.60	132.83	4.90	15.75	1.17E-05	4.88	-3.33E-08	-13.0059	1.97	0.17	0.0324
1355	1217	20120921	12.58	130.33	5.27	22.25	1.95E-05	2.19	2.63E-06	-4.54042	1.56	0.19	0.03778
1356	1217	20120922	11.70	129.60	7.28	27.00	7.33E-05	1.16	7.87E-07	-13.7331	1.44	0.10	0.04005
1357	1217	20120923	12.38	128.85	7.89	46.75	6.01E-05	2.24	2.59E-06	-11.1252	1.39	0.21	0.05478
1358	1217	20120924	14.38	128.23	7.98	59.50	6.23E-05	3.00	2.23E-06	-8.57326	1.06	0.16	0.05846
1359	1217	20120925	16.30	127.50	7.90	64.00	6.06E-05	2.98	2.07E-06	-13.0161	1.14	0.25	0.06446
1360	1218	20120923	16.40	140.13	8.04	15.00	1.16E-05	5.52	-4.30E-07	-0.95503	2.33	0.17	0.03402
1361	1218	20120924	19.80	138.85	10.19	16.50	4.72E-05	4.58	1.96E-06	-5.0231	2.96	0.33	0.0476
1362	1218	20120925	22.35	138.55	7.43	18.00	3.49E-05	3.62	7.23E-07	-1.39824	4.04	0.15	0.04446
1363	1219	20120929	13.05	150.80	8.02	14.00	1.47E-05	4.39	-2.15E-06	-3.82223	2.24	0.27	0.03612
1364	1219	20120930	15.40	149.05	9.73	15.00	1.94E-05	5.78	5.75E-07	-7.57135	2.19	0.17	0.03585
1365	1219	20121001	18.43	145.60	9.45	18.25	2.60E-05	6.19	-1.40E-06	-3.92687	3.09	0.26	0.04294
1366	1219	20121002	21.50	142.33	8.64	20.75	2.49E-05	5.01	3.25E-06	3.818629	4.52	0.25	0.05228
1367	1221	20121005	15.13	143.00	6.03	13.50	1.14E-05	5.17	5.36E-08	-5.01928	1.91	0.21	0.03225
1368	1221	20121006	17.10	140.15	5.25	15.00	1.54E-05	3.90	-1.32E-06	-21.0874	2.68	0.20	0.03658
1369	1221	20121007	17.83	137.10	4.88	17.00	1.79E-05	3.21	-3.57E-07	-17.6578	2.34	0.16	0.03558
1370	1221	20121008	18.05	134.50	5.87	27.75	2.24E-05	3.98	-2.25E-06	-19.8238	1.79	0.26	0.04196
1371	1221	20121009	17.93	132.05	8.62	33.50	7.91E-05	2.63	2.17E-06	-11.1954	1.98	0.20	0.05028
1372	1221	20121010	18.65	130.18	7.99	40.50	6.62E-05	2.61	-9.04E-07	0.293794	2.01	0.15	0.04931
1373	1221	20121011	19.43	128.60	10.32	44.25	1.43E-04	1.23	2.65E-06	1.999863	1.85	0.25	0.06044
1374	1221	20121012	20.08	129.10	11.37	45.75	1.54E-04	2.05	4.42E-06	-3.30432	2.31	0.30	0.06701
1375	1221	20121013	21.23	130.03	12.78	40.00	2.09E-04	2.05	2.93E-06	-8.14039	3.01	0.26	0.06781
1376	1221	20121014	22.43	131.08	8.60	39.50	5.97E-05	1.52	4.23E-07	-1.15292	3.42	0.28	0.06042
1377	1221	20121015	22.50	130.98	9.88	36.50	9.90E-05	2.08	3.46E-07	-4.1584	3.38	0.31	0.06158
1378	1221	20121016	22.10	129.30	8.50	29.50	5.97E-05	2.73	4.09E-06	-4.63765	3.07	0.27	0.05499
1379	1222	20121012	16.20	147.10	7.56	15.00	5.75E-06	1.98	-5.64E-07	-4.88031	2.40	0.07	0.03255
1380	1222	20121013	16.48	145.90	7.27	15.00	1.25E-05	3.03	9.56E-07	-2.32227	2.42	0.11	0.03413
1381	1222	20121014	17.80	143.45	5.63	17.75	2.61E-05	4.38	2.44E-07	0.287436	2.54	0.23	0.03865
1382	1222	20121015	20.93	141.40	9.44	23.00	5.37E-05	9.30	2.78E-06	-1.21094	4.23	0.22	0.05096
1383	1223	20121021	6.65	134.45	2.72	15.00	1.52E-05	5.26	1.40E-06	-12.2714	1.44	0.12	0.02761
1384	1223	20121022	8.28	132.13	5.20	15.00	1.64E-05	5.02	1.29E-06	-12.4662	1.38	0.20	0.03155
1385	1223	20121023	8.45	128.60	5.58	16.50	3.34E-05	4.41	1.33E-06	-10.5657	0.86	0.19	0.03075
1386	1224	20121201	5.40	140.00	9.49	53.00	1.22E-04	6.28	1.87E-06	-4.71231	1.77	0.39	0.06648
1387	1224	20121202	6.38	135.35	9.10	53.25	2.07E-04	7.48	2.22E-06	5.05313	1.59	0.37	0.06684
1388	1224	20121203	7.18	129.55	8.50	55.00	1.62E-04	8.01	3.94E-06	6.201987	0.93	0.31	0.06182
1389	1225	20121223	8.30	133.80	7.54	13.00	2.54E-05	5.81	1.13E-06	-2.97045	1.43	0.27	0.03345
1390	1225	20121224	9.38	131.18	9.40	16.00	2.77E-05	5.68	1.54E-06	15.6267	1.51	0.40	0.03985
1391	1225	20121225	10.25	127.90	9.63	19.00	7.04E-05	7.12	2.40E-06	8.350373	1.31	0.27	0.03846
1392	1301	20130101	5.40	135.65	3.93	11.50	2.34E-05	12.34	2.13E-06	-4.27365	1.67	0.28	0.02977
1393	1301	20130102	7.13	130.10	6.70	13.00	2.47E-05	10.67	-1.10E-07	-7.43544	1.04	0.29	0.02949
1394	1303	20130607	16.23	129.57	5.16	12.00	7.27E-06	2.55	-5.06E-06	-4.93091	1.23	0.33	0.03
1395	1303	20130608	17.73	129.73	8.27	16.50	9.68E-06	4.15	-2.89E-06	6.823748	1.34	0.49	0.03993
1396	1303	20130609	20.73	131.85	8.84	19.50	3.28E-05	4.55	2.29E-06	1.133318	2.34	0.46	0.04931
1397	1303	20130610	22.40	133.40	13.87	23.00	0.0001485	5.00	1.33E-06	0.022253	3.54	0.18	0.0535
1398	1306	20130627	9.03	131.23	4.13	13.00	1.80E-05	5.00	1.48E-06	-5.60221	1.48	0.23	0.031
1399	1306	20130628	9.83	128.43	5.43	15.00	2.38E-05	4.87	2.02E-06	9.808045	1.28	0.34	0.03515

1400	1307	20130707	19.03	149.05	6.94	14.00	1.34E-05	6.73	-4.03E-07	5.928327	2.60	0.21	0.03463
1401	1307	20130708	19.23	143.38	6.08	23.50	1.84E-05	6.14	-2.26E-06	-1.81469	3.48	0.34	0.04738
1402	1307	20130709	20.15	138.75	6.67	42.00	3.32E-05	5.88	6.79E-07	-8.29773	3.02	0.20	0.05435
1403	1307	20130710	21.58	134.10	8.44	55.00	4.05E-05	6.04	1.06E-06	-5.28696	3.65	0.30	0.06912
1404	1307	20130711	22.35	129.00	12.29	48.00	1.30E-04	6.38	8.86E-07	-5.14669	3.03	0.31	0.06774
1405	1311	20130808	12.70	135.90	5.09	15.00	6.02E-06	5.06	3.91E-07	-3.23941	1.81	0.11	0.02902
1406	1311	20130809	13.58	133.23	4.10	17.75	1.01E-05	5.71	4.92E-07	-6.66135	1.97	0.19	0.03323
1407	1311	20130810	13.93	128.75	3.13	34.75	9.62E-06	5.73	-9.29E-08	-12.4358	1.15	0.08	0.03538
1408	1312	20130818	20.15	127.55	9.02	17.25	2.20E-05	1.43	-2.28E-06	-1.77813	2.67	0.38	0.04542
1409	1312	20130819	20.25	128.33	9.61	22.75	6.36E-05	2.65	-5.34E-07	-7.39006	2.72	0.39	0.05181
1410	1312	20130820	21.80	128.30	12.67	28.00	0.0001013	5.32	3.24E-06	-9.94368	3.35	0.42	0.06262
1411	1318	20130911	19.60	148.20	8.08	13.00	8.58E-06	9.02	1.30E-06	-5.03079	2.88	0.12	0.03343
1412	1318	20130912	20.80	145.50	7.05	14.50	1.62E-05	5.23	8.96E-07	-7.27816	3.88	0.29	0.04559
1413	1318	20130913	22.40	141.70	7.73	18.00	3.87E-05	9.51	5.44E-08	-4.897	4.70	0.14	0.04475
1414	1319	20130916	17.30	132.20	7.65	15.33	3.47E-05	2.06	4.75E-07	-4.46678	2.17	0.20	0.03751
1415	1319	20130917	17.45	131.10	10.30	19.75	1.11E-04	1.65	2.94E-06	-6.8015	1.99	0.27	0.04742
1416	1319	20130918	17.18	129.73	8.61	31.00	6.54E-05	2.85	3.99E-08	-13.2454	1.25	0.17	0.04297
1417	1319	20130919	17.57	127.83	10.94	48.67	0.0001086	4.53	8.48E-07	-6.65482	1.37	0.19	0.05631
1418	1320	20130919	15.10	148.97	7.76	11.00	2.67E-05	3.91	1.29E-06	-23.1207	2.13	0.10	0.03243
1419	1320	20130920	18.33	148.28	10.52	14.00	4.84E-05	6.59	1.68E-06	-13.7392	2.57	0.24	0.04133
1420	1320	20130921	20.13	144.90	10.51	17.75	9.69E-05	3.94	2.46E-06	-3.08629	3.81	0.37	0.05546
1421	1320	20130922	22.07	142.70	11.31	24.33	0.0001404	4.05	2.69E-06	-7.64499	4.67	0.17	0.05872
1422	1323	20130929	10.60	133.85	5.68	13.00	1.88E-05	4.92	1.51E-06	-20.1949	1.74	0.19	0.03282
1423	1323	20130930	13.00	132.80	4.86	16.50	2.37E-05	3.79	-2.73E-07	-23.1038	1.92	0.10	0.03198
1424	1323	20131001	15.65	131.45	4.54	24.75	2.15E-05	4.59	-1.38E-06	-28.1467	2.50	0.16	0.04024
1425	1323	20131002	18.70	130.10	9.19	30.00	1.18E-04	3.21	3.59E-06	2.94244	2.00	0.24	0.051
1426	1323	20131003	20.83	129.75	10.07	32.75	9.85E-05	2.72	3.99E-07	0.013988	2.79	0.13	0.05068
1427	1323	20131004	22.45	129.70	9.60	40.00	4.84E-05	2.73	2.51E-07	14.91937	3.02	0.20	0.05497
1428	1324	20131001	17.50	151.80	6.52	13.00	7.50E-06	3.12	-3.66E-07	-16.3951	2.57	0.20	0.03632
1429	1324	20131002	16.98	150.63	6.09	14.00	1.34E-05	2.19	3.58E-07	-19.3644	2.41	0.22	0.03767
1430	1324	20131003	16.10	148.93	5.86	15.00	2.04E-05	4.09	-3.54E-07	-12.7881	2.28	0.24	0.03679
1431	1324	20131004	17.00	145.35	7.59	17.75	2.51E-05	6.14	1.08E-06	-0.84791	2.57	0.17	0.0376
1432	1324	20131005	19.20	140.18	8.17	28.25	3.29E-05	7.96	-5.45E-07	3.783819	3.53	0.26	0.04904
1433	1324	20131006	22.00	135.00	9.58	40.00	3.79E-05	8.28	3.22E-06	3.937865	4.10	0.28	0.0619
1434	1325	20131008	14.10	131.25	5.03	13.00	2.30E-05	3.12	8.05E-07	-6.0564	2.06	0.18	0.03306
1435	1325	20131009	14.48	129.33	5.10	17.00	2.85E-05	3.40	6.53E-07	-12.2546	1.34	0.19	0.03278
1436	1325	20131010	15.30	127.40	8.70	27.50	1.01E-04	4.11	2.47E-06	8.822287	1.11	0.18	0.04146
1437	1326	20131009	13.50	146.30	3.53	13.00	1.47E-05	3.51	-2.46E-08	-22.6422	1.70	0.11	0.02849
1438	1326	20131010	13.63	144.20	6.73	16.50	4.85E-05	4.62	2.68E-06	-19.5707	1.76	0.19	0.03711
1439	1326	20131011	14.58	140.90	10.51	22.00	1.38E-04	4.70	2.03E-06	-20.7401	1.88	0.16	0.04465
1440	1326	20131012	17.05	138.55	8.69	31.50	7.16E-05	3.73	8.89E-07	-13.9699	2.54	0.18	0.04974
1441	1326	20131013	19.48	136.70	13.08	45.75	0.0001694	4.88	4.83E-06	2.382934	2.89	0.33	0.0706
1442	1326	20131014	22.35	135.20	16.37	50.00	0.0001981	7.25	2.82E-06	-7.92766	4.21	0.56	0.08775
1443	1327	20131015	13.20	146.80	5.17	13.00	2.09E-05	8.44	1.39E-07	-20.5664	1.69	0.13	0.028
1444	1327	20131016	11.80	144.03	7.55	17.75	5.89E-05	4.08	-2.84E-07	-19.3766	1.72	0.13	0.03522
1445	1327	20131017	11.93	142.73	7.07	38.75	5.35E-05	3.68	6.04E-07	-11.4897	1.65	0.20	0.04873
1446	1327	20131018	14.78	141.78	10.74	56.25	1.36E-04	4.07	3.19E-06	-2.7411	1.90	0.19	0.06516
1447	1327	20131019	16.88	139.38	8.78	61.00	6.73E-05	3.92	1.52E-06	-6.23351	2.53	0.44	0.07458
1448	1327	20131020	18.58	137.10	7.63	56.25	3.97E-05	3.10	1.97E-06	4.687188	2.50	0.20	0.06214
1449	1327	20131021	20.65	135.75	8.55	44.25	8.95E-05	4.07	1.42E-06	3.964863	3.68	0.16	0.06048
1450	1327	20131022	22.50	134.05	7.19	40.00	3.35E-05	3.91	3.54E-06	-6.53478	3.88	0.24	0.06112
1451	1328	20131021	13.80	159.10	11.37	30.00	3.55E-05	8.74	-1.58E-06	-6.32756	2.85	0.23	0.04806
1452	1328	20131022	16.63	156.08	11.60	49.50	2.47E-05	8.54	1.44E-06	3.059799	2.67	0.25	0.05993
1453	1328	20131023	19.25	150.38	9.98	60.00	8.93E-05	6.20	-6.20E-08	9.72218	3.05	0.17	0.06568
1454	1328	20131024	21.53	146.57	13.16	59.33	0.0001955	6.80	2.29E-06	12.18939	4.32	0.17	0.07749
1455	1329	20131027	12.50	142.20	4.40	13.00	1.65E-05	11.86	5.81E-07	-8.00094	1.69	0.26	0.02956
1456	1329	20131028	14.73	138.03	6.34	13.50	1.79E-05	7.85	-1.64E-07	-7.29901	2.05	0.14	0.03
1457	1329	20131029	15.60	132.70	5.13	15.75	2.22E-05	5.90	-1.02E-06	-0.11338	2.34	0.28	0.03636
1458	1329	20131030	16.60	129.10	10.49	23.00	9.74E-05	6.68	-2.30E-06	-2.64361	1.05	0.35	0.0418
1459	1330	20131103	6.10	154.63	4.00	14.33	2.90E-05	6.84	1.20E-06	3.391211	0.55	0.17	0.02458
1460	1330	20131104	6.33	149.63	4.44	21.50	4.93E-05	7.71	2.29E-06	-0.5648	0.30	0.17	0.02901
1461	1330	20131105	6.78	143.73	3.84	37.50	4.37E-05	8.15	1.86E-06	-5.00006	0.68	0.14	0.03805
1462	1330	20131106	7.78	137.03	6.41	56.00	7.44E-05	8.99	3.00E-06	-5.38373	1.32	0.25	0.05776
1463	1330	20131107	9.70	130.00	11.25	71.50	2.98E-04	9.33	2.66E-06	7.84402	1.46	0.35	0.07951
1464	1401	20140117	9.70	127.63	9.46	13.00	6.63E-05	1.30	4.16E-06	-12.1497	1.24	0.51	0.0472
1465	1401	20140118	8.78	127.85	9.38	17.25	4.70E-05	2.65	5.93E-06	-2.8778	1.02	0.41	0.04469
1466	1401	20140119	7.20	128.10	7.74	15.33	2.79E-05	1.54	4.82E-06	3.127088	0.88	0.42	0.04092
1467	1402	20140129	9.43	141.25	5.69	13.00	2.42E-05	7.11	-6.11E-08	6.639048	1.84	0.29	0.03282
1468	1402	20140130	8.60	135.35	5.40	14.50	4.68E-05	9.55	1.98E-06	1.139004	1.26	0.36	0.03461
1469	1402	20140131	9.75	129.30	6.27	19.00	4.85E-05	12.46	-2.97E-07	23.61039	1.33	0.45	0.03704
1470	1403	20140227	8.70	147.90	7.68	13.00	3.70E-05	1.29	1.61E-06	2.965223	0.85	0.12	0.02851
1471	1403	20140228	8.60	148.58	8.13	16.50	4.07E-05	2.03	2.56E-06	-4.1306	0.77	0.19	0.03326
1472	1403	20140301	8.90	149.75	7.76	19.00	4.16E-05	2.25	1.66E-06	-8.25373	0.54	0.25	0.03519
1473	1403	20140302	9.80	149.13	8.26	23.00	7.25E-05	3.00	3.93E-06	-8.49935	0.75	0.25	0.04081
1474	1403	20140303	12.45	149.50	8.72	25.75	8.62E-05	4.97	1.57E-06	3.213091	2.12	0.23	0.04532
1475	1403	20140304	16.80	150.90	9.51	31.50	7.50E-05	7.61	7.31E-07	-14.9347	2.40	0.09	0.04516
1476	1403	20140305	21.35	153.70	11.37	24.75	6.30E-05	5.51	1.34E-06	-31.5649	3.35	0.23	0.05297
1477	1403	20140306	23.00	155.90	10.92	15.00	8.06E-05	6.65	1.00E-06	-22.6638	3.88	0.19	0.04741
1478	1404	20140405	5.15	138.20	6.09	18.00	6.33E-05	4.92	1.87E-06	10.00223	1.66	0.24	0.0367
1479	1404	20140406	6.58	135.60	6.62	18.00	8.38E-05	5.02	4.90E-06	6.139466	1.65	0.45	0.04662
1480	1404	20140407	6.65	132.15	8.15	18.00	0.0001103	3.56	1.19E-05	0.283992	1.12	0.63	0.05701
1481	1404	20140408	7.70	130.38	8.46	18.00	6.06E-05	2.09	1.69E-06	3.873626	1.05	0.51	0.04582
1482	1404	20140409	8.20	130.40	9.25	16.00	4.01E-05	0.90	1.00E-06	5.734672	1.13	0.31	0.03811
1483	1405	20140427	11.10	146.55	6.09	14.50	1.22E-05	3.33	4.06E-07	7.802619	0.89	0.22	0.02892
1484	1405	20140428	13.60	147.25	7.27	21.50	1.48E-05	4.13	7.26E-07	3.739535	1.77	0.15	0.03529
1485	1405	20140429	16.80	147.38	9.38	29.50	1.04E-05	4.00	-1.26E-06	-0.03682	2.55	0.18	0.04466
1486	1405	20140430	19.58	146.88	9.41	24.00	9.77E-06	4.31	2.91E-06	3.091324	3.30	0.37	0.05298
1487	1405	20140501	22.03	144.37	7.09	14.33	7.96E-06	5.40	1.34E-06	0.009272	4.92	0.23	0.04734
1488	1408	20140702	8.25	145.85	6.99	13.00	1.85E-05	4.19	2.28E-07	-6.85166	1.06	0.23	0.03046
1489	1408	20140703	10.13	144.13	7.49	15.75	3.64E-05	5.39	1.18E-06	-7.46591	1.64	0.13	0.03238
1490	1408	20140704	13.40	140.80	10.98	27.75	1.94E-04	6.38	1.00E-06	-4.7354	1.97	0.17	0.04835
1491	1408	20140705	16.35	136.45	14.18	43.75	0.0002978	7.40	1.25E-06	-2.67196	1.70	0.20	0.06231
1492	1408	20140706	18.75	131.00	13.87	50.75	0.0002064	6.71	2.74E-06	-11.0768	2.32	0.28	0.07017
1493	1408	20140707	21.05	127.80	18.20	55.00	0.0004444	7.41	6.43E-06	-3.72209	3.23	0.71	0.10238
1494	1409	20140710	10.20	150.98	3.83	13.50	9.36E-06	6.59	1.25E-06	9.898912	0.79	0.29	0.02791
1495	1409	20140711	12.70	146.85	2.85	15.00	6.36E-06	5.14	2.75E-07	-3.16841	1.55	0.16	0.02817
1496	1409	20140712	13.45	142.35	4.53	18.25	1.82E-05	7.78	4.25E-07	-7.63175	1.75	0.28	0.03529
1497	1409	20140713	13.55	135.45	5.18	21.50	1.68E-05	9.81	8.22E-07	3.392005	1.89	0.24	0.03541
1498	1409	20140714	13.00	129.37	4.55	30.00	2.19E-05	6.06	4.69E-07	0.42319	1.34	0.30	0.04107
1499	1410	20140717	10.00	135.60	6.95	15.75	5.14E-05	1.57	1.41E-07	-2.51447	1.49	0.26	0.03676

1500	1410	20140718	10.80	133.88	7.35	20.75	6.40E-05	4.66	4.76E-07	-12.1817	1.77	0.29	0.04207
1501	1410	20140719	11.45	130.80	11.54	31.50	0.0001157	3.36	-6.44E-07	-13.2456	1.46	0.36	0.05327
1502	1410	20140720	13.93	128.80	10.34	35.00	8.61E-05	5.94	3.12E-07	-4.76216	1.16	0.16	0.04452
1503	1410	20140721	16.70	127.20	8.70	35.00	6.52E-05	8.90	3.59E-06	-7.34908	1.31	0.46	0.05406
1504	1411	20140728	11.73	149.88	5.94	13.50	1.70E-05	3.62	1.37E-06	7.183242	1.73	0.17	0.03108
1505	1411	20140729	12.98	147.43	6.93	19.00	4.62E-05	4.25	1.18E-06	0.48109	1.73	0.24	0.03803
1506	1411	20140730	14.25	144.13	6.49	23.00	3.41E-05	4.51	1.45E-06	2.478692	1.78	0.20	0.03863
1507	1411	20140731	14.93	140.90	4.54	26.00	1.62E-05	3.77	-5.24E-07	-10.4942	1.88	0.15	0.0372
1508	1411	20140801	14.68	138.08	6.22	32.75	2.63E-05	3.53	-2.85E-07	-15.3273	2.04	0.13	0.04318
1509	1411	20140802	15.05	135.50	7.24	53.75	1.40E-05	3.17	8.41E-07	-17.4599	1.72	0.15	0.05526
1510	1411	20140803	15.73	132.70	9.33	59.25	7.12E-05	4.27	-2.00E-06	-15.1581	2.36	0.21	0.06399
1511	1411	20140804	17.35	130.25	7.89	44.75	5.18E-05	3.80	1.64E-06	-10.092	1.64	0.24	0.05431
1512	1411	20140805	20.48	129.90	8.34	40.00	4.73E-05	4.21	2.12E-06	-8.44134	2.60	0.16	0.05328
1513	1411	20140806	22.40	130.30	7.51	42.00	4.11E-05	3.71	1.24E-06	-6.40279	3.19	0.24	0.05836
1514	1412	20140729	18.73	130.63	6.51	15.25	2.67E-05	3.70	1.99E-06	-13.9535	2.19	0.24	0.03864
1515	1412	20140730	21.67	129.07	6.22	18.00	1.87E-05	10.29	2.77E-07	-6.81898	3.21	0.16	0.03746
1516	1415	20140910	10.00	141.00	6.14	13.00	4.63E-06	10.80	-4.08E-07	-6.89401	1.89	0.25	0.0307
1517	1415	20140911	12.90	137.05	4.58	13.50	2.28E-05	10.16	-6.81E-07	-8.73566	1.80	0.28	0.03137
1518	1415	20140912	13.70	130.63	5.30	19.00	1.97E-05	4.54	-9.24E-07	-11.0526	1.78	0.17	0.03365
1519	1415	20140913	14.35	128.10	6.69	27.50	2.65E-05	6.98	1.02E-06	11.43199	1.06	0.29	0.03875
1520	1416	20140917	12.73	130.90	7.32	16.00	2.78E-05	6.95	-1.49E-06	1.357438	1.68	0.24	0.03283
1521	1416	20140918	14.20	127.75	9.26	18.00	5.26E-05	7.19	-2.27E-07	11.91875	1.08	0.31	0.03566
1522	1417	20140923	18.60	149.80	6.28	13.00	1.17E-05	3.23	3.90E-06	2.960984	2.68	0.23	0.03878
1523	1417	20140924	19.83	149.70	8.13	16.00	1.94E-05	2.97	4.12E-06	-0.76853	2.95	0.27	0.04479
1524	1417	20140925	20.60	147.38	7.57	20.00	2.67E-05	3.46	4.17E-06	-0.44374	3.83	0.29	0.05131
1525	1417	20140926	22.10	146.05	7.30	21.50	3.24E-05	4.34	1.79E-06	-1.93072	4.37	0.24	0.05122
1526	1418	20140928	11.77	155.10	6.87	13.67	1.19E-05	8.49	1.60E-07	-3.89138	2.04	0.23	0.03269
1527	1418	20140929	13.35	150.73	4.28	19.00	1.03E-05	6.05	1.07E-07	-12.373	2.29	0.13	0.03367
1528	1418	20140930	16.10	146.28	6.84	24.75	3.74E-05	6.21	6.49E-07	-8.90865	2.40	0.16	0.04071
1529	1418	20141001	18.08	141.93	7.03	34.50	3.46E-05	6.68	2.65E-06	0.493012	2.98	0.35	0.05524
1530	1418	20141002	20.35	137.73	6.58	45.00	3.32E-05	5.70	3.24E-06	6.746922	2.96	0.20	0.05631
1531	1418	20141003	22.30	135.40	6.99	45.00	3.92E-05	5.95	-1.17E-06	-0.06385	4.18	0.21	0.06015
1532	1419	20141003	8.55	157.90	4.25	18.50	2.42E-05	6.02	9.59E-07	5.298088	1.21	0.14	0.02867
1533	1419	20141004	10.73	153.43	8.01	27.25	5.17E-05	7.61	5.85E-07	9.266024	1.36	0.27	0.04049
1534	1419	20141005	13.40	147.55	8.52	36.50	6.81E-05	8.97	4.74E-07	1.249616	1.86	0.27	0.04882
1535	1419	20141006	16.18	140.60	7.29	39.00	5.00E-05	8.95	1.71E-07	-14.4836	2.23	0.15	0.04734
1536	1419	20141007	17.35	134.75	9.86	59.00	1.15E-04	5.30	1.02E-06	-9.94598	1.46	0.29	0.06533
1537	1419	20141008	18.35	131.25	9.56	67.25	1.25E-04	3.20	-8.78E-07	-3.74055	2.16	0.27	0.07201
1538	1419	20141009	19.98	129.70	12.59	58.75	2.41E-04	3.00	4.86E-06	-0.86178	2.29	0.29	0.0775
1539	1419	20141010	21.85	129.35	10.16	51.00	7.39E-05	4.64	1.21E-06	-0.93834	3.15	0.15	0.06207
1540	1420	20141030	12.50	139.40	4.55	13.50	1.33E-05	4.81	-2.33E-06	-10.9127	1.87	0.22	0.03075
1541	1420	20141031	12.70	135.93	6.11	19.00	2.98E-05	4.20	1.60E-06	-5.06787	1.81	0.19	0.03653
1542	1420	20141101	13.85	133.48	7.46	31.75	3.80E-05	3.56	8.42E-07	0.730908	1.98	0.15	0.04349
1543	1420	20141102	16.70	132.55	11.07	59.00	1.12E-04	3.75	1.50E-07	3.921046	2.38	0.20	0.06618
1544	1420	20141103	19.48	133.28	9.05	63.50	5.82E-05	4.77	3.02E-07	-22.0425	2.59	0.28	0.07078
1545	1420	20141104	22.17	135.50	13.55	56.67	2.65E-04	5.14	1.29E-06	3.211565	4.11	0.11	0.07635
1546	1422	20141201	5.55	149.75	3.79	21.50	2.88E-05	9.43	3.00E-06	-9.85977	0.00	0.57	0.04005
1547	1422	20141202	6.25	144.43	4.73	31.00	3.47E-05	8.99	6.60E-06	1.162755	0.56	0.54	0.04904
1548	1422	20141203	8.48	137.75	11.33	47.50	0.0001809	8.88	3.42E-06	11.96259	1.31	0.26	0.05869
1549	1422	20141204	10.65	131.98	13.60	65.75	0.0002079	5.85	3.54E-06	8.669165	1.60	0.26	0.07422
1550	1422	20141205	11.90	128.78	15.11	58.75	0.0001611	2.84	5.78E-06	-1.82581	1.39	0.69	0.08585
1551	1422	20141206	12.10	127.40	13.31	55.00	0.0002184	3.07	7.37E-06	-1.46446	1.36	0.52	0.07976
1552	1501	20150113	8.78	141.88	8.47	14.00	6.15E-05	4.39	2.37E-07	2.208088	1.69	0.46	0.04281
1553	1501	20150114	10.68	137.80	8.76	18.00	4.10E-05	6.77	1.02E-06	21.68722	1.93	0.37	0.04092
1554	1501	20150115	11.60	133.18	6.50	20.00	3.38E-05	5.21	1.37E-06	23.95332	1.85	0.33	0.03964
1555	1501	20150116	11.33	129.18	7.21	26.50	5.18E-05	5.24	4.46E-07	22.4719	1.40	0.39	0.04359
1556	1502	20150206	8.65	158.05	5.55	13.00	2.88E-05	3.57	-1.28E-08	15.62696	1.27	0.17	0.02739
1557	1502	20150207	10.53	157.60	7.61	15.75	3.03E-05	3.14	1.14E-06	12.98865	1.95	0.18	0.03461
1558	1502	20150208	12.15	156.95	7.80	24.00	2.26E-05	1.27	7.07E-07	-15.1112	2.38	0.29	0.04698
1559	1502	20150209	12.60	155.85	7.80	34.50	6.22E-05	2.76	9.67E-07	3.188019	2.34	0.26	0.05154
1560	1502	20150210	14.40	154.15	7.60	41.50	3.68E-05	2.94	-1.25E-07	8.025415	2.52	0.18	0.05172
1561	1502	20150211	15.90	152.68	7.18	20.75	2.81E-05	2.53	1.31E-07	17.62682	2.45	0.15	0.03781
1562	1502	20150212	17.15	152.40	5.56	13.00	2.81E-05	4.09	-6.39E-07	-36.6816	2.53	0.14	0.03514
1563	1503	20150313	9.73	158.67	11.22	20.00	0.0001728	8.96	3.83E-06	9.415702	1.86	0.23	0.04454
1564	1503	20150314	12.93	152.23	13.60	21.50	0.0001939	9.36	1.28E-06	19.02956	2.11	0.45	0.05356
1565	1503	20150315	13.75	145.58	12.82	22.25	2.07E-04	9.32	1.54E-06	65.38376	1.71	0.38	0.04674
1566	1503	20150316	13.95	139.28	9.91	20.00	1.04E-04	6.38	1.87E-06	48.23753	2.04	0.28	0.04116
1567	1503	20150317	15.48	134.85	8.73	17.25	3.36E-05	4.32	8.30E-07	43.37523	1.65	0.27	0.03519
1568	1503	20150318	16.03	131.53	5.99	14.00	2.18E-05	3.77	1.54E-06	15.85611	2.57	0.17	0.03449
1569	1503	20150319	15.53	128.55	6.16	13.00	2.29E-05	4.63	2.22E-07	3.948332	1.15	0.19	0.02844
1570	1504	20150326	6.50	159.20	8.54	14.00	7.81E-05	4.72	6.91E-07	-8.39999	0.56	0.20	0.0311
1571	1504	20150327	7.15	157.03	8.89	16.50	5.15E-05	3.51	3.03E-07	3.954425	0.80	0.12	0.02956
1572	1504	20150328	7.53	154.00	5.24	27.75	2.90E-05	4.37	4.45E-06	-6.3303	0.64	0.25	0.03939
1573	1504	20150329	7.83	150.18	8.09	37.00	0.0001137	6.13	1.54E-06	0.169282	0.31	0.14	0.04118
1574	1504	20150330	8.90	145.35	11.78	44.75	0.0002274	6.31	4.45E-06	11.48789	1.19	0.38	0.06421
1575	1504	20150331	10.05	140.58	14.73	62.00	0.0004193	6.31	3.89E-06	12.56131	1.90	0.66	0.094
1576	1504	20150401	11.40	136.15	13.24	57.25	0.0002548	5.00	3.80E-06	16.54517	1.78	0.64	0.0838
1577	1504	20150402	13.05	132.98	12.67	49.25	0.000169	4.46	2.54E-06	32.4291	1.93	0.42	0.06803
1578	1504	20150403	14.23	129.43	11.11	38.75	1.21E-04	5.79	2.25E-06	22.42422	1.34	0.27	0.05206
1579	1505	20150403	8.00	155.33	5.26	15.25	4.74E-05	5.48	3.74E-06	3.414258	0.69	0.23	0.03099
1580	1505	20150404	8.83	152.38	5.20	22.25	4.55E-05	2.25	2.60E-06	1.229748	0.77	0.21	0.03542
1581	1505	20150405	8.93	150.93	6.68	20.25	6.12E-05	0.96	1.93E-06	2.474596	0.30	0.19	0.03293
1582	1505	20150406	9.05	150.60	5.99	15.00	5.47E-05	0.51	2.78E-06	7.336629	0.31	0.17	0.02919
1583	1506	20150502	7.65	142.60	4.65	13.00	3.49E-05	2.33	3.42E-06	-0.41778	1.25	0.22	0.03248
1584	1506	20150503	8.70	141.35	4.92	15.75	1.80E-05	3.23	-9.07E-07	-2.94588	1.75	0.43	0.03981
1585	1506	20150504	9.63	139.75	7.15	21.50	3.91E-05	1.59	2.72E-06	-2.60075	1.81	0.35	0.04559
1586	1506	20150505	9.40	138.60	6.68	26.50	5.70E-05	1.79	2.70E-06	0.068973	1.72	0.40	0.04962
1587	1506	20150506	9.75	136.43	6.14	34.75	5.10E-05	4.60	1.77E-06	9.858751	1.40	0.62	0.05725
1588	1506	20150507	11.33	132.63	10.41	43.50	1.43E-04	5.46	1.61E-06	12.57034	1.75	0.46	0.06332
1589	1506	20150508	12.95	128.60	5.14	46.50	2.93E-05	5.63	-1.10E-06	6.196688	1.26	0.38	0.05224
1590	1507	20150510	7.60	159.93	7.35	18.00	8.67E-05	3.98	4.47E-06	-0.94205	1.33	0.21	0.03847
1591	1507	20150511	9.63	159.35	6.78	20.75	6.89E-05	2.33	2.48E-06	0.458043	2.04	0.30	0.04463
1592	1507	20150512	10.05	157.60	7.35	25.25	9.63E-05	2.82	7.41E-07	-4.7513	1.77	0.23	0.04422
1593	1507	20150513	10.48	154.73	6.13	37.00	3.74E-05	5.36	9.90E-07	3.913098	1.53	0.38	0.05051
1594	1507	20150514	12.15	150.30	11.25	40.00	9.795E-05	7.24	5.51E-07	7.844476	2.04	0.48	0.06119
1595	1507	20150515	13.98	145.18	9.04	43.50	8.03E-05	6.04	1.77E-06	20.06323	1.74	0.32	0.05514
1596	1507	20150516	16.18	141.05	13.73	55.75	2.45E-04	5.28	-9.40E-07	-4.79739	2.25	0.31	0.07279
1597	1507	20150517	18.98	138.65	10.40	55.75	7.81E-05	4.16	8.30E-07	3.963962	2.88	0.29	0.06818
1598	1507	20150518	21.67	138.63	12.97	42.67	8.945E-05	3.85	6.88E-06	-0.68375	3.72	0.43	0.07495
1599	1509	20150630	10.05	159.10	7.29	19.00	3.23E-05	5.26	6.48E-07	8.31391	2.10	0.32	0.0407

1600	1509	20150701	11.03	156.00	6.40	22.00	2.84E-05	6.18	-1.30E-07	-14.8506	1.94	0.36	0.04325
1601	1509	20150702	10.70	150.45	7.92	27.75	6.32E-05	5.97	2.95E-06	-6.18636	1.07	0.35	0.0455
1602	1509	20150703	11.03	148.45	7.76	24.75	8.75E-05	2.74	1.71E-06	-7.60711	1.06	0.59	0.05311
1603	1509	20150704	13.03	146.90	7.15	24.00	5.27E-05	4.68	5.62E-07	-1.44539	1.67	0.20	0.03938
1604	1509	20150705	14.75	144.13	7.18	25.75	5.07E-05	4.39	1.25E-06	1.563819	1.78	0.12	0.03853
1605	1509	20150706	17.15	140.45	13.16	30.75	1.98E-04	6.72	3.25E-06	-9.61572	2.74	0.29	0.06028
1606	1509	20150707	18.50	135.90	12.82	34.50	0.0001868	5.07	2.57E-06	-7.58207	2.00	0.38	0.06185
1607	1509	20150708	20.63	132.13	15.02	37.00	0.0002704	6.25	3.97E-06	-11.8495	2.33	0.54	0.07476
1608	1509	20150709	22.50	129.60	12.40	40.00	0.0001465	5.94	-1.86E-06	-16.8536	2.99	0.47	0.06825
1609	1510	20150701	13.30	129.90	5.84	13.00	2.09E-05	3.25	4.52E-07	-11.1814	1.51	0.17	0.03054
1610	1510	20150702	14.43	128.63	7.18	16.00	2.62E-05	4.25	1.25E-06	-13.053	1.14	0.21	0.03329
1611	1511	20150706	12.10	158.33	5.71	35.75	4.10E-05	6.00	1.16E-06	4.847993	2.42	0.37	0.05274
1612	1511	20150707	14.00	153.90	5.40	53.25	3.03E-05	6.61	-3.41E-07	2.430291	2.44	0.15	0.05422
1613	1511	20150708	15.88	149.38	10.79	54.25	1.21E-04	5.42	5.88E-07	-3.14166	2.26	0.32	0.06724
1614	1511	20150709	17.70	145.20	15.39	60.00	2.77E-04	5.38	8.26E-07	-17.8915	2.85	0.39	0.08448
1615	1511	20150710	18.33	141.63	12.43	53.25	2.14E-04	3.34	3.51E-06	-0.90946	3.16	0.32	0.07715
1616	1511	20150711	18.38	139.18	13.56	41.75	2.39E-04	2.73	4.95E-06	0.199483	2.94	0.41	0.07515
1617	1511	20150712	18.70	137.50	13.85	40.00	2.59E-04	1.92	-8.72E-07	-17.4121	2.60	0.29	0.06782
1618	1511	20150713	20.93	136.88	10.98	42.00	9.22E-05	4.17	3.57E-07	-15.9711	3.38	0.20	0.06159
1619	1511	20150714	22.80	136.50	11.59	42.00	9.90E-05	3.11	1.84E-06	-9.65809	3.94	0.24	0.06669
1620	1512	20150717	18.30	158.87	8.68	18.00	2.77E-05	5.16	4.20E-06	1.725144	2.84	0.33	0.04687
1621	1512	20150718	18.03	155.08	7.63	15.00	1.14E-05	5.89	-1.14E-06	3.772427	2.55	0.19	0.03465
1622	1512	20150719	19.53	150.85	8.39	15.00	1.29E-05	5.65	8.72E-08	9.018061	3.28	0.23	0.04014
1623	1512	20150720	21.43	146.80	7.44	22.75	2.51E-05	5.38	-2.84E-06	15.16653	4.31	0.27	0.04818
1624	1512	20150721	22.60	142.83	6.51	34.33	2.10E-05	5.77	7.54E-07	13.72273	5.05	0.21	0.05733
1625	1513	20150730	13.60	159.27	9.48	17.00	1.20E-05	3.02	8.18E-07	-6.75302	2.75	0.34	0.04586
1626	1513	20150731	13.40	156.43	8.96	18.00	2.06E-05	6.06	-1.89E-08	-1.30313	2.67	0.35	0.04413
1627	1513	20150801	14.08	151.43	7.47	21.00	2.95E-05	6.52	6.40E-07	-7.26378	2.32	0.34	0.04408
1628	1513	20150802	15.08	146.43	7.68	36.50	6.92E-05	5.67	1.27E-06	-10.6855	2.10	0.19	0.04951
1629	1513	20150803	17.08	142.38	7.36	57.50	3.99E-05	6.07	2.19E-06	0.135373	2.38	0.25	0.06277
1630	1513	20150804	18.78	137.83	12.12	64.00	1.66E-04	5.98	9.86E-06	2.770773	2.66	0.52	0.08791
1631	1513	20150805	19.90	133.20	11.51	49.25	0.0001424	5.84	3.54E-06	-0.98205	2.73	0.33	0.06927
1632	1513	20150806	20.87	129.20	12.14	47.00	0.0001339	5.11	5.33E-06	-4.30385	2.85	0.37	0.07153
1633	1515	20150814	12.55	149.30	5.23	15.75	2.04E-05	4.32	2.79E-06	-3.5189	2.12	0.21	0.03627
1634	1515	20150815	14.00	146.10	7.38	23.25	7.27E-05	3.46	1.39E-06	-4.45389	1.71	0.27	0.04337
1635	1515	20150816	15.33	143.78	9.70	34.50	1.08E-04	4.39	2.46E-06	2.113513	1.88	0.26	0.05259
1636	1515	20150817	17.18	139.78	10.02	52.00	6.52E-05	7.23	1.17E-07	7.554656	2.67	0.33	0.06386
1637	1515	20150818	18.60	133.70	10.02	50.00	9.49E-05	7.78	3.31E-06	1.018608	2.16	0.23	0.06035
1638	1515	20150819	18.87	128.53	12.19	51.67	0.0001929	6.02	2.46E-07	-18.0012	1.53	0.28	0.065
1639	1516	20150816	14.45	159.70	13.32	33.00	0.0001715	2.06	4.49E-06	-0.03762	2.87	0.37	0.06618
1640	1516	20150817	15.03	158.15	17.68	45.00	0.0004675	4.24	6.17E-06	-5.5585	3.04	0.39	0.08748
1641	1516	20150818	16.80	155.03	17.17	52.75	0.0003397	5.39	6.82E-06	-15.4611	2.49	0.48	0.08823
1642	1516	20150819	19.45	151.58	18.16	59.25	0.000417	5.27	3.08E-06	-13.3248	3.45	0.34	0.09239
1643	1516	20150820	21.90	148.57	18.14	58.33	0.0004223	4.79	1.95E-06	-16.9298	4.03	0.45	0.09788
1644	1518	20150906	20.88	139.25	5.91	13.50	1.70E-05	4.02	-6.46E-07	13.98028	3.46	0.14	0.0357
1645	1518	20150907	22.90	138.60	6.28	18.00	2.07E-05	4.99	3.32E-06	7.616912	4.38	0.24	0.04826
1646	1520	20150914	15.80	150.77	5.74	13.00	1.26E-05	4.85	3.28E-06	-1.37174	2.35	0.24	0.03657
1647	1520	20150915	18.28	150.00	4.99	15.75	1.18E-05	3.57	-4.73E-07	-5.57446	2.71	0.11	0.03366
1648	1520	20150916	19.88	146.90	4.86	27.75	1.30E-05	5.03	-8.23E-08	-6.02025	3.49	0.16	0.04523
1649	1520	20150917	22.03	143.83	7.19	42.00	1.55E-05	4.64	3.37E-06	8.324507	5.08	0.32	0.06785
1650	1521	20150921	15.50	141.10	6.22	13.00	1.52E-05	6.41	5.27E-07	-5.58209	1.99	0.49	0.04162
1651	1521	20150922	17.05	138.45	7.09	15.75	2.97E-05	4.17	8.72E-07	-3.63388	2.52	0.44	0.04572
1652	1521	20150923	18.10	134.80	7.74	18.50	5.46E-05	4.11	2.98E-06	-14.1538	1.75	0.29	0.04246
1653	1521	20150924	18.70	132.65	9.20	25.25	9.20E-05	1.80	6.78E-06	-4.25882	1.99	0.38	0.05469
1654	1521	20150925	19.68	131.48	10.26	34.00	1.32E-04	3.01	3.79E-06	-13.7733	2.34	0.41	0.06261
1655	1521	20150926	21.68	129.28	11.60	46.25	1.42E-04	3.96	-6.63E-07	-13.55	3.17	0.27	0.06671
1656	1521	20150927	22.30	127.50	18.44	55.00	0.0003496	3.38	1.08E-06	-5.36083	3.35	0.34	0.08643
1657	1523	20151004	21.17	157.17	7.88	25.33	3.73E-05	6.98	-2.09E-06	-2.56811	3.07	0.21	0.0437
1658	1523	20151005	22.25	153.23	6.31	28.00	2.79E-05	4.38	1.13E-06	-7.78381	3.97	0.26	0.05314
1659	1524	20151012	14.70	148.08	5.67	13.50	2.69E-05	10.02	5.57E-07	5.915611	2.00	0.19	0.02978
1660	1524	20151013	15.73	140.00	6.12	16.50	3.38E-05	8.07	2.68E-06	4.872789	2.03	0.46	0.04305
1661	1524	20151014	15.63	134.35	6.78	18.00	5.16E-05	5.60	2.28E-06	5.330937	1.72	0.30	0.03934
1662	1524	20151015	15.48	129.73	5.98	27.25	3.35E-05	5.51	1.31E-06	-0.34307	1.44	0.41	0.0455
1663	1524	20151016	15.70	127.30	5.11	38.00	2.49E-05	3.62	1.22E-06	-10.3821	1.12	0.19	0.04369
1664	1525	20151013	13.40	158.60	5.77	16.50	1.38E-05	6.26	7.32E-07	-2.32942	2.76	0.32	0.04098
1665	1525	20151014	14.73	155.53	5.75	18.50	2.82E-05	5.73	-7.02E-07	0.725874	2.73	0.21	0.03801
1666	1525	20151015	15.98	150.55	7.89	23.50	5.03E-05	7.08	-1.67E-07	-10.4624	2.32	0.31	0.04499
1667	1525	20151016	16.23	144.83	10.53	31.50	1.20E-04	5.92	7.52E-08	-10.2658	2.19	0.27	0.05202
1668	1525	20151017	17.98	141.28	8.16	39.50	6.06E-05	3.94	9.54E-08	-13.1007	3.09	0.19	0.05563
1669	1525	20151018	19.68	140.15	11.74	50.00	1.36E-04	1.90	3.68E-06	0.800581	3.53	0.31	0.07406
1670	1525	20151019	20.85	140.25	8.08	47.50	4.77E-05	1.25	4.15E-06	-7.98695	3.82	0.28	0.06899
1671	1525	20151020	21.90	139.93	8.41	42.25	5.05E-05	1.75	1.85E-06	-8.85037	4.28	0.29	0.06689
1672	1525	20151021	22.80	139.85	7.98	40.00	4.44E-05	1.13	9.93E-07	-7.6434	4.72	0.33	0.06811
1673	1526	20151117	5.20	158.30	5.16	20.00	3.94E-05	6.35	1.55E-06	-2.85767	0.12	0.49	0.03801
1674	1526	20151118	6.18	155.35	4.74	24.50	2.90E-05	6.35	6.89E-07	-3.59255	0.42	0.26	0.03345
1675	1526	20151119	8.83	151.08	6.56	27.75	3.70E-05	5.95	6.13E-07	6.363586	0.33	0.13	0.03144
1676	1526	20151120	10.55	146.65	7.70	41.75	3.10E-05	7.74	1.92E-06	1.636926	0.83	0.40	0.05097
1677	1526	20151121	12.13	140.43	8.62	51.50	3.75E-05	8.34	1.57E-06	6.119332	1.91	0.34	0.05939
1678	1526	20151122	14.43	134.58	10.73	45.75	5.88E-05	6.56	4.91E-06	3.272263	1.87	0.32	0.06017
1679	1526	20151123	16.93	131.55	9.97	42.00	1.14E-05	3.93	4.39E-07	13.8483	2.32	0.29	0.05473
1680	1526	20151124	19.10	133.23	7.90	35.75	3.02E-05	4.27	2.13E-06	-11.5909	2.32	0.27	0.0526
1681	1526	20151125	20.48	136.38	7.80	22.25	3.42E-05	5.51	3.37E-07	-20.4789	3.41	0.41	0.05332
1682	1526	20151126	22.50	138.50	10.21	15.00	3.94E-05	6.56	-8.85E-07	-17.4986	4.09	0.39	0.05164
1683	1527	20151210	7.70	141.85	7.50	14.00	2.03E-05	8.18	-3.49E-07	-3.60367	1.52	0.54	0.04143
1684	1527	20151211	9.33	137.43	6.12	17.75	2.96E-05	7.05	1.30E-06	2.395756	1.42	0.16	0.03102
1685	1527	20151212	10.98	132.85	7.38	24.00	2.41E-05	5.65	-9.01E-07	-3.87972	1.75	0.41	0.04435
1686	1527	20151213	12.20	128.60	10.59	41.75	8.88E-05	5.48	-2.86E-07	0.19286	1.40	0.18	0.04987
1687	1601	20160702	8.35	145.25	6.28	15.00	1.07E-05	1.73	6.51E-07	-19.6114	1.17	0.11	0.02969
1688	1601	20160703	9.70	144.38	11.27	19.00	6.02E-05	5.21	-3.54E-07	-9.17542	1.53	0.19	0.038
1689	1601	20160704	13.15	140.30	11.37	24.00	3.33E-05	8.47	-4.82E-07	3.701959	1.99	0.36	0.04515
1690	1601	20160705	16.68	134.30	11.82	46.75	2.14E-05	9.03	-5.34E-07	-0.94072	1.63	0.22	0.05183
1691	1601	20160706	19.43	128.57	9.20	70.67	3.19E-05	8.35	-1.33E-06	0.639246	1.87	0.29	0.06713
1692	1605	20160803	17.70	148.23	5.89	13.67	-2.59E-08	2.14	1.88E-07	-1.13835	2.55	0.17	0.03453
1693	1605	20160804	19.08	148.48	6.70	17.25	1.03E-05	2.95	1.99E-06	-2.38108	2.63	0.18	0.0389
1694	1605	20160805	21.43	147.57	5.67	22.67	2.43E-06	5.50	-3.98E-06	-9.99561	4.23	0.20	0.04456
1695	1606	20160809	17.25	158.18	11.60	20.25	4.58E-05	4.52	-2.92E-06	-6.49536	2.98	0.20	0.04366
1696	1606	20160810	18.83	154.85	9.30	23.00	3.15E-05	3.97	9.17E-07	2.946679	2.61	0.15	0.04181
1697	1606	20160811	20.80	154.03	6.78	23.00	2.79E-05	5.60	-2.47E-06	13.31873	3.25	0.25	0.04287
1698	1607	20160813	18.88	140.93	7.68	15.25	4.36E-05	5.77	9.62E-07	1.034503	3.53	0.16	0.04049
1699	1607	20160814	21.90	143.40	9.84	19.00	2.80E-05	10.39	2.62E-06	7.318879	4.92	0.39	0.0557

1700	1609	20160817	15.18	140.18	8.25	12.75	2.78E-05	3.91	-5.80E-08	1.089871	1.90	0.21	0.0337
1701	1609	20160818	16.03	142.73	5.65	15.00	2.15E-05	3.56	5.81E-07	-16.1523	2.06	0.14	0.03361
1702	1609	20160819	18.35	141.45	6.40	18.00	2.37E-05	4.91	1.52E-06	-5.13278	3.23	0.16	0.04067
1703	1609	20160820	21.30	140.90	6.70	21.50	2.84E-05	9.28	1.16E-06	4.708646	4.20	0.25	0.04749
1704	1614	20160909	13.60	142.08	4.83	14.00	1.29E-05	4.01	6.61E-07	0.989467	1.80	0.09	0.02819
1705	1614	20160910	15.15	138.55	8.03	18.50	2.64E-05	6.00	2.03E-06	-4.60135	2.15	0.14	0.03643
1706	1614	20160911	17.00	133.75	7.68	35.25	2.36E-05	6.88	7.85E-07	-10.4737	1.82	0.28	0.04797
1707	1614	20160912	18.40	129.33	8.52	57.33	2.91E-05	6.13	2.97E-06	1.187096	1.41	0.23	0.05858
1708	1616	20160911	12.50	144.70	4.76	13.00	2.21E-05	7.70	2.21E-07	-1.16602	1.79	0.15	0.02801
1709	1616	20160912	13.33	141.35	4.98	14.50	2.25E-05	6.89	1.28E-06	3.565791	1.89	0.16	0.03028
1710	1616	20160913	14.63	136.18	8.21	21.50	2.18E-05	6.00	1.25E-06	4.595526	1.84	0.15	0.03593
1711	1616	20160914	16.10	131.63	10.72	34.00	6.31E-05	6.09	1.34E-06	7.86302	2.58	0.19	0.05028
1712	1616	20160915	17.35	128.25	7.16	38.00	4.50E-05	5.12	1.00E-06	0.351472	1.22	0.17	0.044
1713	1617	20160922	15.40	141.20	8.90	13.00	2.27E-05	3.63	-8.86E-08	-6.04939	1.97	0.17	0.03386
1714	1617	20160923	15.98	139.08	9.95	18.50	3.31E-05	5.62	5.95E-07	-11.5422	2.29	0.20	0.03997
1715	1617	20160924	18.25	134.63	10.76	27.75	4.80E-05	6.55	-5.82E-07	-8.90657	1.87	0.15	0.04154
1716	1617	20160925	19.95	129.93	9.28	38.00	5.86E-05	5.51	4.89E-07	-5.65599	2.36	0.30	0.0547
1717	1617	20160926	20.80	127.30	11.08	42.00	9.78E-05	5.06	2.57E-06	-10.1617	3.14	0.34	0.06571
1718	1618	20160925	15.70	159.70	4.88	13.00	1.27E-05	4.91	4.03E-06	-20.2254	2.73	0.26	0.04017
1719	1618	20160926	15.80	156.88	6.77	13.50	2.13E-05	7.01	1.98E-07	-16.2702	2.83	0.16	0.03562
1720	1618	20160927	14.53	151.60	7.11	15.75	2.47E-05	6.27	1.36E-06	-1.48486	2.39	0.13	0.03433
1721	1618	20160928	14.48	146.18	6.20	18.00	2.69E-05	6.49	3.21E-06	-9.01521	1.88	0.22	0.03734
1722	1618	20160929	13.98	141.23	5.88	20.25	2.34E-05	6.47	1.45E-07	-20.7412	1.89	0.13	0.03453
1723	1618	20160930	14.43	137.08	5.64	25.75	1.78E-05	4.86	2.95E-06	-18.722	1.90	0.30	0.04487
1724	1618	20161001	17.25	134.10	9.17	33.25	5.72E-05	6.49	-4.24E-07	17.069	1.66	0.14	0.04121
1725	1618	20161002	21.20	129.90	7.55	52.00	4.68E-05	10.23	-2.13E-06	22.18182	3.00	0.16	0.05416
1726	1620	20161007	19.30	158.70	6.73	13.00	1.67E-05	7.31	-1.59E-06	-2.49579	2.91	0.18	0.03389
1727	1620	20161008	19.88	155.05	5.88	16.50	1.28E-05	7.72	2.22E-06	-2.16915	2.95	0.24	0.03956
1728	1620	20161009	21.60	150.50	7.64	21.50	2.17E-05	7.71	8.88E-07	-4.42162	4.41	0.26	0.0501
1729	1621	20161012	12.50	129.70	6.65	15.00	3.65E-06	2.28	2.74E-07	-14.7308	1.47	0.10	0.02997
1730	1621	20161013	13.38	128.70	5.27	16.50	1.73E-05	4.01	8.69E-08	-7.76076	1.18	0.12	0.02837
1731	1622	20161014	7.55	144.15	6.03	14.00	2.41E-05	4.37	2.16E-06	-4.87687	1.02	0.12	0.02769
1732	1622	20161015	9.28	141.75	6.64	21.50	4.70E-05	5.16	7.87E-07	-9.30382	1.78	0.09	0.0347
1733	1622	20161016	10.95	138.80	8.90	35.25	0.0001069	5.25	2.07E-06	-5.32902	1.82	0.24	0.05122
1734	1622	20161017	13.60	135.05	8.21	53.25	7.12E-05	6.19	8.06E-07	-2.03086	1.95	0.19	0.05747
1735	1622	20161018	15.45	129.93	8.75	64.50	5.88E-05	7.82	2.30E-06	11.50405	1.53	0.31	0.065
1736	1623	20161031	10.30	143.85	5.14	13.00	7.85E-06	4.74	3.10E-07	-16.2156	1.68	0.16	0.0299
1737	1623	20161101	11.38	140.60	5.40	14.50	9.58E-06	5.93	5.52E-07	-12.6853	1.83	0.16	0.03101
1738	1623	20161102	12.65	137.73	4.28	15.00	1.68E-05	1.54	-1.80E-06	-14.1246	1.83	0.17	0.03174
1739	1623	20161103	13.78	137.35	4.27	18.50	1.55E-05	2.96	-2.60E-08	-21.665	1.85	0.16	0.0343
1740	1623	20161104	15.80	139.00	6.26	25.25	2.74E-05	3.66	1.07E-06	-10.2217	2.27	0.14	0.04049
1741	1623	20161105	17.93	140.65	7.04	36.00	3.24E-05	4.27	1.01E-06	-8.84533	3.22	0.14	0.05088
1742	1623	20161106	20.80	143.67	9.87	41.33	6.148E-05	9.44	9.99E-07	-3.97588	4.68	0.09	0.05887
1743	1624	20161109	15.67	158.77	9.46	13.67	2.48E-05	6.35	3.53E-06	-8.90403	2.95	0.20	0.04092
1744	1624	20161110	17.48	155.13	8.10	16.50	1.91E-05	6.18	4.15E-07	-13.083	2.53	0.24	0.03941
1745	1624	20161111	19.50	149.53	8.66	18.00	1.67E-05	8.64	6.68E-07	-2.27414	2.79	0.25	0.04047
1746	1624	20161112	21.40	144.57	7.84	14.33	9.40E-06	5.85	-1.32E-06	-14.6828	4.54	0.24	0.04587
1747	1626	20161220	6.43	144.50	7.74	13.00	1.57E-05	4.23	2.23E-06	7.051048	0.61	0.19	0.0276
1748	1626	20161221	6.83	141.30	6.24	15.75	5.91E-05	5.46	1.83E-06	20.14672	1.61	0.22	0.03371
1749	1626	20161222	9.80	137.03	7.46	22.75	4.27E-05	8.34	1.41E-06	16.44264	1.54	0.20	0.0355
1750	1626	20161223	12.38	131.55	10.93	33.25	3.85E-05	5.58	2.59E-06	-6.7954	1.69	0.30	0.05071
1751	1626	20161224	13.30	128.23	13.42	56.00	0.0001241	3.82	2.33E-06	7.517038	1.14	0.43	0.06942
1752	1701	20170422	8.90	144.10	5.14	13.00	4.66E-06	5.25	8.39E-07	-3.26537	1.41	0.16	0.02774
1753	1701	20170423	10.38	142.05	6.45	13.00	7.17E-06	5.39	-1.35E-07	1.271075	1.77	0.22	0.03121
1754	1701	20170424	12.28	138.58	8.93	15.00	7.99E-06	3.70	-1.18E-06	-13.1298	1.77	0.28	0.03694
1755	1701	20170425	13.05	136.55	8.93	15.75	2.03E-05	1.89	-8.80E-07	5.862892	1.84	0.23	0.03612
1756	1701	20170426	13.75	134.85	8.08	18.00	1.19E-05	3.30	-5.82E-07	4.328033	1.94	0.22	0.03624
1757	1701	20170427	16.40	134.60	7.48	16.00	8.87E-06	4.90	-7.19E-07	2.710902	1.58	0.22	0.03269
1758	1701	20170428	19.53	137.18	8.17	13.00	8.73E-06	7.06	6.88E-07	0.12716	2.87	0.41	0.04299
1759	1701	20170429	22.25	141.15	7.73	13.00	1.41E-05	10.50	7.26E-07	-0.03382	4.62	0.33	0.04667
1760	1705	20170730	22.85	141.15	5.62	47.00	2.18E-05	2.40	-1.72E-06	-1.14974	5.00	0.29	0.06747
1761	1705	20170731	22.90	139.87	5.60	52.00	2.35E-05	2.74	3.58E-06	-5.65493	4.78	0.30	0.07279
1762	1709	20170725	13.68	128.53	7.92	13.00	4.40E-05	3.66	-6.75E-07	-29.5158	1.12	0.17	0.03137
1763	1709	20170726	16.30	127.88	9.24	19.00	2.42E-05	2.76	3.81E-06	-14.5699	1.11	0.36	0.04311
1764	1709	20170727	18.07	127.37	5.94	26.00	1.92E-05	6.28	-3.82E-06	-9.44246	1.47	0.31	0.03848
1765	1715	20170827	18.95	147.05	3.74	13.00	6.10E-06	1.58	3.42E-06	-6.5387	2.91	0.28	0.04065
1766	1715	20170828	20.03	147.25	5.35	17.75	1.09E-05	3.10	4.31E-06	-10.5538	3.53	0.29	0.0476
1767	1715	20170829	22.15	147.80	7.89	21.50	1.18E-05	4.61	3.11E-07	-9.08429	4.34	0.32	0.05266
1768	1718	20170909	15.23	143.48	6.42	16.50	1.49E-05	6.02	-1.67E-06	-1.99721	1.92	0.15	0.03089
1769	1718	20170910	16.60	139.00	7.53	22.75	1.45E-05	6.11	-2.76E-07	-2.61264	2.48	0.26	0.04181
1770	1718	20170911	19.35	133.93	8.23	30.25	1.44E-05	7.95	1.55E-06	0.139612	2.83	0.22	0.04679
1771	1718	20170912	21.93	129.03	6.81	36.00	2.43E-05	6.88	2.26E-06	-2.79091	3.15	0.23	0.05246
1772	1721	20171015	9.20	136.90	6.26	15.33	4.58E-05	3.49	1.62E-06	-8.99553	1.31	0.21	0.03389
1773	1721	20171016	10.48	134.78	9.54	19.50	3.88E-05	4.04	2.04E-06	-0.67395	1.67	0.32	0.04254
1774	1721	20171017	10.43	132.53	8.21	26.50	0.0001041	3.24	2.24E-06	-16.7812	1.62	0.17	0.04456
1775	1721	20171018	13.40	131.70	10.85	32.00	0.0001576	5.22	4.28E-06	-17.3706	1.92	0.29	0.05666
1776	1721	20171019	16.60	130.05	11.62	38.00	0.0001646	4.51	2.69E-06	0.506787	1.45	0.21	0.05422
1777	1721	20171020	19.90	130.23	12.16	46.75	0.0001486	3.72	2.90E-06	-10.6878	2.43	0.38	0.07006
1778	1721	20171021	21.80	131.60	15.12	58.00	0.0001793	6.40	1.63E-07	-16.222	3.11	0.51	0.08394
1779	1722	20171022	9.07	148.13	6.81	13.00	3.76E-05	1.22	1.54E-06	-9.28271	0.86	0.16	0.03003
1780	1722	20171023	9.55	146.88	7.25	13.50	3.08E-05	6.00	2.53E-06	-16.3348	0.97	0.20	0.03119
1781	1722	20171024	13.15	140.58	8.48	17.25	3.69E-05	11.21	-4.87E-07	-2.70534	2.00	0.14	0.03181
1782	1722	20171025	15.88	134.78	5.88	20.75	2.39E-05	6.12	1.42E-06	21.09539	1.59	0.32	0.03767
1783	1722	20171026	19.08	131.58	6.79	23.00	3.27E-05	5.99	3.87E-06	9.849108	2.29	0.39	0.04731
1784	1722	20171027	22.05	129.40	9.09	25.00	5.95E-05	6.81	1.05E-06	2.152721	3.10	0.26	0.04843
1785	1726	20171213	11.18	128.75	6.08	13.50	3.78E-05	1.66	3.44E-08	2.350879	1.37	0.18	0.03105
1786	1726	20171214	11.43	127.85	7.66	17.25	6.60E-05	0.87	1.67E-06	-1.98476	1.36	0.31	0.04078
1787	1726	20171215	11.88	127.50	7.41	21.50	5.68E-05	1.90	2.53E-06	7.343262	1.35	0.27	0.0406
1788	1727	20171220	8.63	132.18	8.40	14.75	3.61E-05	5.41	1.99E-06	-5.74712	1.40	0.46	0.04235
1789	1727	20171221	8.23	128.73	9.56	22.67	9.662E-05	5.42	3.06E-06	-2.17736	0.81	0.35	0.04383
1790	1801	20171230	9.60	135.10	8.12	13.00	2.70E-05	5.08	3.27E-07	6.605456	1.48	0.34	0.03549
1791	1801	20171231	9.30	132.63	5.58	13.00	2.13E-05	5.59	1.24E-06	2.206499	1.47	0.37	0.03562
1792	1801	20180101	8.85	129.05	5.08	14.00	3.10E-05	7.66	3.91E-06	-0.63315	1.03	0.35	0.03429
1793	1802	20180210	7.43	141.40	9.17	14.50	5.70E-05	8.43	3.67E-06	-1.14365	1.64	0.37	0.04089
1794	1802	20180211	6.88	135.20	8.70	17.25	1.18E-04	6.98	7.77E-06	0.153945	1.53	0.40	0.04772
1795	1802	20180212	7.50	129.70	6.22	18.00	5.10E-05	8.30	5.33E-06	4.480372	0.92	0.35	0.0376
1796	1803	20180324	5.75	143.15	6.40	13.00	3.00E-05	5.45	-7.37E-07	-4.66297	0.71	0.18	0.02616
1797	1803	20180325	6.58	139.90	6.74	17.25	4.50E-05	5.54	2.38E-07	12.06859	1.80	0.22	0.03454
1798	1803	20180326	8.35	136.43	7.76	18.00	8.054E-05	4.06	5.22E-06	-4.62554	1.18	0.32	0.0421
1799	1803	20180327	10.30	135.13	7.14	18.50	6.15E-05	3.85	1.70E-06	-2.94429	1.62	0.41	0.0442

1800	1803	20180328	13.95	135.23	8.16	25.25	2.62E-05	3.64	1.21E-06	-8.36716	1.91	0.18	0.04131
1801	1803	20180329	15.40	135.98	9.09	35.75	7.59E-05	2.42	2.53E-06	-1.71534	1.67	0.29	0.05274
1802	1803	20180330	16.58	138.55	10.61	56.00	6.25E-05	5.40	-1.54E-07	5.35584	2.47	0.25	0.06378
1803	1803	20180331	18.75	141.88	9.83	32.75	4.98E-06	4.84	-3.09E-07	-10.3242	3.36	0.36	0.05679
1804	1803	20180401	19.40	145.15	10.01	14.00	1.92E-05	6.65	-2.16E-06	-20.271	3.45	0.17	0.03977
1805	1805	20180606	14.10	130.10	5.10	13.00	1.27E-05	4.48	5.67E-08	-14.7503	1.68	0.22	0.03192
1806	1805	20180607	16.38	128.28	5.37	15.00	1.52E-05	3.02	1.64E-07	-14.0157	1.06	0.23	0.03148
1807	1805	20180608	18.25	127.35	5.98	18.00	3.03E-05	10.00	-1.04E-06	-12.1057	1.46	0.29	0.03406
1808	1805	20180609	22.17	127.57	7.62	24.33	4.12E-05	4.13	5.92E-07	-3.6103	3.37	0.36	0.05242
1809	1807	20180628	19.83	131.80	4.90	14.00	1.28E-05	3.16	4.06E-06	-5.13357	2.27	0.44	0.04421
1810	1807	20180629	20.03	130.03	4.84	19.00	1.46E-05	1.60	3.03E-06	-2.40095	2.46	0.37	0.04546
1811	1807	20180630	21.63	128.95	7.27	23.00	2.44E-05	4.67	4.95E-06	-3.41717	3.25	0.27	0.04961
1812	1808	20180703	10.78	147.48	3.60	13.00	8.57E-06	2.91	4.00E-07	-15.9767	0.85	0.11	0.02456
1813	1808	20180704	12.45	145.85	5.73	17.75	2.03E-05	4.48	-8.70E-08	-6.60413	1.52	0.20	0.03307
1814	1808	20180705	14.75	143.10	7.31	36.75	4.27E-05	3.68	8.76E-07	-2.75859	1.85	0.21	0.04798
1815	1808	20180706	16.53	141.43	8.40	57.25	4.44E-05	2.27	2.47E-06	-14.8727	2.41	0.23	0.06587
1816	1808	20180707	17.78	140.33	11.09	52.00	4.48E-05	3.28	8.40E-07	-1.91959	3.12	0.22	0.06484
1817	1808	20180708	20.10	137.10	11.24	56.50	5.14E-05	7.37	5.27E-06	3.649505	3.04	0.40	0.07374
1818	1808	20180709	22.37	132.00	10.84	57.00	5.90E-05	8.10	2.38E-06	2.927997	3.34	0.30	0.07017
1819	1810	20180718	19.43	129.60	7.09	16.50	3.38E-05	3.16	-1.95E-06	-10.4015	1.93	0.39	0.04158
1820	1810	20180719	21.15	131.40	8.81	21.50	5.02E-05	3.81	3.95E-06	-0.56295	2.71	0.34	0.05019
1821	1810	20180720	22.60	130.80	7.03	25.00	2.94E-05	3.20	3.43E-06	4.349418	3.32	0.18	0.04748
1822	1812	20180723	16.90	136.75	4.51	14.00	9.52E-06	4.62	-2.99E-06	-6.90049	1.88	0.19	0.02953
1823	1812	20180724	19.33	136.68	6.06	16.50	9.35E-06	3.73	-1.40E-08	-1.44115	2.81	0.25	0.03947
1824	1812	20180725	21.68	137.18	5.64	21.50	1.94E-05	2.56	1.53E-06	-6.38981	3.45	0.37	0.05096
1825	1812	20180726	22.55	138.75	6.39	29.00	1.33E-05	4.99	-1.62E-06	-0.98244	4.26	0.35	0.05509
1826	1813	20180802	18.03	152.03	4.30	13.50	1.05E-05	2.57	-1.47E-06	-11.0518	2.83	0.21	0.03576
1827	1813	20180803	18.35	150.25	7.26	22.75	2.97E-05	3.70	2.26E-08	-11.7331	2.80	0.17	0.0427
1828	1813	20180804	21.30	148.50	6.42	32.75	1.76E-05	4.26	6.36E-07	-14.4681	3.75	0.20	0.05264
1829	1813	20180805	22.90	147.40	7.95	38.00	2.38E-05	2.50	5.82E-07	-12.8143	4.63	0.14	0.05942
1830	1814	20180807	19.03	132.75	6.33	14.50	2.55E-05	1.28	-3.01E-06	-8.35953	2.19	0.43	0.04199
1831	1814	20180808	19.78	133.50	8.02	17.25	2.89E-05	3.38	-2.85E-06	-4.40306	2.91	0.44	0.04724
1832	1814	20180809	21.25	133.45	9.31	18.00	3.86E-05	3.39	1.76E-08	-3.26904	3.06	0.32	0.04684
1833	1814	20180810	22.80	132.10	6.96	18.00	3.04E-05	0.95	1.73E-06	-2.92866	3.44	0.19	0.04467
1834	1815	20180810	17.65	144.60	5.60	14.00	1.06E-05	3.32	-6.85E-07	-2.27617	2.74	0.18	0.03511
1835	1815	20180811	19.63	143.65	8.74	17.00	2.34E-05	4.44	-1.76E-06	1.534403	3.83	0.23	0.04401
1836	1815	20180812	22.20	142.35	10.14	23.00	2.30E-05	6.12	-4.51E-06	0.138287	4.71	0.34	0.05358
1837	1819	20180815	12.80	144.20	6.64	13.67	2.78E-05	6.54	2.86E-07	-9.10362	1.79	0.27	0.03475
1838	1819	20180816	17.75	142.10	9.23	21.00	4.16E-05	8.61	-4.15E-07	14.23282	2.76	0.34	0.04437
1839	1819	20180817	22.20	140.65	9.71	26.50	4.37E-05	7.56	-6.11E-07	-2.93794	4.53	0.29	0.0555
1840	1820	20180817	12.60	156.77	3.25	13.00	1.01E-05	6.21	2.91E-07	-4.06755	2.48	0.26	0.03401
1841	1820	20180818	14.05	153.83	5.32	16.50	1.96E-05	3.84	4.56E-07	-13.0893	2.44	0.15	0.03573
1842	1820	20180819	15.80	150.98	7.92	21.50	3.75E-05	4.66	1.04E-06	-9.03475	2.37	0.29	0.04471
1843	1820	20180820	18.03	147.65	7.85	29.00	3.08E-05	6.06	3.77E-07	-3.39174	2.68	0.10	0.04235
1844	1820	20180821	21.53	143.38	10.69	39.00	1.20E-04	8.15	4.34E-06	0.878202	4.75	0.27	0.06835
1845	1821	20180827	13.35	158.90	4.99	14.50	6.53E-06	5.56	-1.22E-06	-0.17379	2.74	0.10	0.03082
1846	1821	20180828	16.20	155.75	8.83	21.50	2.24E-05	5.05	7.60E-08	-5.02157	2.64	0.18	0.04117
1847	1821	20180829	17.25	151.88	9.42	35.25	1.53E-05	5.24	-2.56E-06	-6.27817	2.51	0.21	0.04795
1848	1821	20180830	17.73	147.40	8.32	50.00	1.94E-05	6.15	8.26E-07	-3.94701	2.70	0.15	0.05601
1849	1821	20180831	18.45	142.20	8.62	64.25	4.24E-05	6.54	-1.84E-06	2.129408	3.10	0.24	0.06774
1850	1821	20180901	20.65	137.85	8.19	57.75	3.54E-05	5.58	2.86E-06	10.451	3.01	0.17	0.06339
1851	1821	20180902	22.60	135.90	8.81	50.00	3.32E-05	5.43	-1.81E-06	10.69684	4.22	0.21	0.06304
1852	1822	20180908	14.70	158.15	5.96	25.50	1.91E-05	10.00	1.64E-06	1.892042	3.01	0.13	0.03993
1853	1822	20180909	15.00	152.18	7.51	34.75	2.08E-05	9.63	-3.73E-07	3.532677	2.45	0.27	0.0472
1854	1822	20180910	14.28	145.08	6.51	43.25	3.96E-05	8.05	1.00E-06	-6.87556	1.78	0.17	0.04825
1855	1822	20180911	13.93	139.30	7.35	60.00	5.59E-05	6.23	1.36E-06	-0.64375	2.04	0.34	0.06592
1856	1822	20180912	14.15	134.50	12.44	65.00	2.53E-04	6.19	-3.37E-08	-11.1372	1.92	0.33	0.07739
1857	1822	20180913	14.85	129.60	9.45	65.00	1.04E-04	5.85	2.84E-06	-10.943	1.47	0.39	0.07251
1858	1824	20180920	13.35	145.55	7.73	13.00	3.80E-05	5.94	-1.80E-07	-3.48115	1.72	0.16	0.03089
1859	1824	20180921	15.10	143.03	9.22	17.75	6.45E-05	5.62	2.59E-06	-1.81786	1.91	0.20	0.03916
1860	1824	20180922	16.58	138.15	9.93	27.75	3.60E-05	6.53	-1.86E-06	4.543072	2.40	0.30	0.04632
1861	1824	20180923	17.50	133.70	6.83	43.75	2.81E-05	4.99	1.12E-06	4.345836	1.82	0.33	0.05402
1862	1824	20180924	19.05	130.13	5.71	57.00	2.59E-05	3.58	4.63E-06	4.481347	2.12	0.34	0.06532
1863	1824	20180925	19.93	128.83	11.52	56.75	1.99E-04	1.48	4.76E-07	3.423266	2.26	0.25	0.07058
1864	1824	20180926	21.10	129.15	14.99	48.00	0.0002785	1.25	3.43E-07	2.639109	3.00	0.18	0.07135
1865	1824	20180927	21.85	128.85	14.16	45.00	0.0002278	1.86	2.56E-06	-6.65155	3.27	0.33	0.07512
1866	1824	20180928	22.50	127.90	11.29	45.00	0.0001083	3.25	5.57E-06	-0.5356	3.30	0.46	0.07438
1867	1825	20180928	9.18	148.73	5.65	14.00	3.30E-05	10.02	2.69E-06	-2.91648	0.79	0.18	0.02652
1868	1825	20180929	12.68	141.95	4.70	19.50	2.10E-05	7.71	-1.51E-06	-4.20106	1.74	0.20	0.03205
1869	1825	20180930	14.60	137.60	4.66	35.75	2.10E-05	4.53	1.29E-06	-16.3878	1.99	0.31	0.04994
1870	1825	20181001	16.43	134.75	9.14	56.00	1.62E-04	4.65	7.87E-08	-13.2361	1.54	0.37	0.06773
1871	1825	20181002	18.53	131.60	8.08	61.00	7.05E-05	4.86	-4.62E-07	-12.3942	2.18	0.25	0.06523
1872	1825	20181003	21.25	128.95	8.61	45.50	6.31E-05	5.06	3.79E-06	15.98839	3.15	0.40	0.06609
1873	1826	20181021	8.48	158.18	3.25	14.00	1.11E-05	4.40	6.68E-07	-4.02198	1.23	0.15	0.0269
1874	1826	20181022	10.35	154.48	7.77	22.75	2.57E-05	6.64	1.33E-06	-2.42506	1.44	0.25	0.03824
1875	1826	20181023	12.15	150.20	10.79	40.75	1.43E-04	5.17	9.94E-07	-7.36312	2.05	0.24	0.05741
1876	1826	20181024	14.33	146.68	11.01	67.50	0.0001233	5.75	3.61E-06	-4.31975	1.85	0.26	0.07295
1877	1826	20181025	16.18	142.78	11.01	60.75	0.0001254	4.91	2.59E-06	-1.49122	2.08	0.38	0.0734
1878	1826	20181026	17.18	138.38	12.28	59.00	0.0001615	7.01	4.19E-06	14.45947	2.54	0.36	0.07477
1879	1826	20181027	17.95	132.85	17.85	62.00	0.0003615	5.78	-1.95E-06	14.03481	1.84	0.30	0.07842
1880	1826	20181028	17.83	128.60	20.73	51.25	0.0003924	5.05	6.37E-06	18.54158	1.21	0.42	0.08108
1881	1828	20181120	5.50	153.10	4.06	18.00	1.84E-05	7.71	1.28E-06	-4.69578	0.85	0.51	0.03869
1882	1828	20181121	8.05	147.75	8.30	23.25	5.95E-05	12.83	-6.14E-07	14.96195	0.71	0.11	0.02734
1883	1828	20181122	12.03	139.13	10.26	33.25	6.19E-05	8.69	1.03E-07	10.52862	1.76	0.21	0.04459
1884	1828	20181123	15.90	135.73	10.27	38.00	4.62E-05	4.77	4.36E-07	17.22547	1.56	0.17	0.04581
1885	1828	20181124	18.40	136.00	10.14	41.00	7.39E-05	2.08	4.60E-07	0.908943	1.95	0.42	0.06068
1886	1828	20181125	18.50	135.23	11.50	36.25	3.75E-05	4.12	-1.26E-06	17.51686	2.00	0.42	0.05476
1887	1828	20181126	20.65	132.38	8.16	19.00	1.06E-05	4.26	2.15E-06	23.4166	2.35	0.25	0.03937
1888	1828	20181127	22.60	132.30	7.32	15.00	1.10E-05	4.34	-4.02E-07	-19.9388	3.40	0.21	0.04166
1889	1829	20181117	7.00	136.80	8.31	13.00	6.53E-05	6.33	3.26E-06	4.749698	1.57	0.17	0.0331
1890	1829	20181118	6.78	134.23	6.93	13.00	1.03E-04	4.85	4.52E-06	9.54434	1.40	0.25	0.03658
1891	1829	20181119	8.90	130.73	7.47	13.50	4.86E-05	5.44	2.16E-06	11.3904	1.39	0.34	0.03641
1892	1829	20181120	10.15	128.15	6.67	15.00	5.11E-05	4.82	2.86E-06	2.419228	1.32	0.28	0.03604
1893	1902	20190219	5.30	159.90	7.53	13.00	5.51E-05	4.82	3.50E-07	-2.45685	0.27	0.20	0.0273
1894	1902	20190220	5.35	152.10	9.52	29.00	0.0002198	5.81	1.25E-06	26.20141	0.68	0.21	0.04321
1895	1902	20190221	6.80	149.23	13.24	36.00	0.0003629	5.59	5.17E-06	22.82451	0.36	0.33	0.05935
1896	1902	20190222	9.45	145.53	14.91	43.00	8.80E-05	5.29	5.69E-06	4.839515	1.15	0.55	0.06719
1897	1902	20190223	11.63	143.00	14.34	54.00	0.0002604	3.27	8.13E-06	-0.02085	1.68	0.66	0.08756
1898	1902	20190224	12.98	141.43	14.97	52.50	0.0003028	2.34	7.46E-06	2.279086	1.88	0.53	0.08485
1899	1902	20190225	14.00	140.10	12.23	57.25	0.0001609	2.07	6.26E-06	2.748255	1.95	0.38	0.07589

1900	1902	20190226	15.30	140.00	12.75	49.25	1.24E-04	1.53	2.60E-06	-5.48062	1.93	0.39	0.06928
1901	1902	20190227	16.30	139.15	9.72	30.50	1.84E-05	4.43	5.57E-08	13.4043	2.38	0.32	0.04906
1902	1902	20190228	17.88	135.48	7.50	15.75	1.04E-05	4.10	-2.17E-06	28.49348	1.56	0.17	0.02839
1903	1902	20190301	19.43	134.13	7.17	13.50	1.31E-05	1.06	3.45E-08	7.663007	2.96	0.30	0.0415
1904	1902	20190302	19.15	133.50	8.87	13.00	1.93E-05	3.89	-8.29E-07	4.704683	2.50	0.22	0.03637
1905	1905	20190714	13.85	134.88	4.44	13.00	1.77E-05	5.22	-7.10E-07	-27.5188	1.93	0.18	0.03153
1906	1905	20190715	16.50	130.48	5.88	15.00	1.98E-05	9.84	3.47E-06	-13.848	1.71	0.35	0.03767
1907	1908	20190801	18.77	154.13	8.55	13.67	2.65E-05	4.71	5.63E-06	-7.15596	2.77	0.49	0.05076
1908	1908	20190802	21.38	151.48	11.50	19.00	0.0001203	6.84	4.50E-07	-5.36405	4.65	0.41	0.06049
1909	1909	20190803	15.80	131.50	8.94	13.00	4.49E-05	4.31	3.41E-06	-20.422	2.55	0.23	0.04171
1910	1909	20190804	17.10	130.43	9.15	16.50	7.357E-05	2.26	3.28E-06	-17.7566	1.66	0.30	0.04381
1911	1909	20190805	18.23	129.83	13.56	22.75	0.0001588	1.62	2.94E-06	-17.666	1.59	0.26	0.0514
1912	1909	20190806	19.35	128.78	14.93	29.00	0.000196	2.46	7.09E-06	-6.66639	1.76	0.42	0.06405
1913	1909	20190807	20.97	127.60	21.68	42.67	0.000425	4.35	4.81E-06	-9.03528	3.20	0.39	0.0871
1914	1910	20190805	17.00	144.80	8.93	15.00	4.53E-05	5.00	1.19E-07	-25.8293	2.51	0.19	0.03944
1915	1910	20190806	18.73	142.73	12.98	19.00	0.0001429	4.31	4.24E-06	-9.22867	3.19	0.31	0.05598
1916	1910	20190807	21.15	141.15	19.47	29.00	0.0004147	2.24	1.25E-06	2.923101	4.20	0.35	0.07858
1917	1910	20190808	22.05	140.65	28.86	41.00	0.0008234	0.88	6.15E-06	2.479894	4.44	0.44	0.11212
1918	1910	20190809	22.25	141.30	27.27	39.25	0.0006529	0.96	7.56E-06	-3.00251	4.64	0.44	0.10651
1919	1910	20190810	22.88	140.90	18.94	33.25	0.000302	1.44	8.33E-06	-3.32551	4.99	0.44	0.08803
1920	1911	20190820	13.93	133.25	8.23	15.00	4.85E-05	2.55	2.63E-06	-20.3617	1.97	0.20	0.03928
1921	1911	20190821	15.10	130.90	7.00	17.25	3.44E-05	3.92	-4.50E-07	-27.8193	2.17	0.21	0.03874
1922	1911	20190822	16.40	128.35	9.57	22.00	7.86E-05	4.83	1.45E-06	-3.8164	1.04	0.25	0.03988
1923	1912	20190825	12.20	135.95	6.47	13.00	1.98E-05	6.54	-9.39E-08	-14.1447	1.80	0.33	0.03616
1924	1912	20190826	13.40	130.43	6.06	13.00	2.94E-05	8.18	1.06E-06	-14.1781	1.68	0.24	0.03264
1925	1913	20190831	7.80	132.30	7.87	13.00	4.32E-05	7.58	1.08E-06	-2.39539	1.36	0.13	0.02853
1926	1913	20190901	12.78	128.50	8.86	14.00	4.86E-05	6.96	2.10E-06	8.284758	1.30	0.19	0.0319
1927	1915	20190903	17.40	160.00	8.54	13.00	4.81E-05	3.59	5.70E-07	-10.891	2.58	0.18	0.03819
1928	1915	20190904	18.20	158.03	8.28	14.50	5.80E-05	4.53	-3.53E-07	-0.03153	2.95	0.12	0.03702
1929	1915	20190905	20.30	154.83	10.59	19.00	8.882E-05	6.70	-1.88E-06	6.873288	3.09	0.24	0.04412
1930	1915	20190906	22.50	151.80	19.75	20.00	0.0003216	8.13	1.03E-06	-0.22457	4.96	0.39	0.07272
1931	1916	20190913	12.23	156.08	4.78	13.00	1.12E-05	7.20	-4.57E-07	-7.47677	2.30	0.20	0.03156
1932	1916	20190914	13.78	151.50	8.70	13.50	7.945E-05	5.10	1.68E-06	-8.51924	2.29	0.17	0.03792
1933	1916	20190915	17.38	148.33	9.45	18.00	7.12E-05	9.29	-9.25E-07	18.31084	2.52	0.13	0.03507
1934	1916	20190916	21.30	145.30	9.16	18.00	5.33E-05	11.03	1.48E-06	7.929779	4.21	0.16	0.04384
1935	1917	20190917	20.80	128.83	7.55	13.00	3.22E-05	1.80	3.19E-06	1.120337	2.96	0.23	0.04185
1936	1917	20190918	22.05	129.15	8.23	14.50	4.67E-05	2.50	2.92E-06	-8.37299	3.08	0.25	0.0448
1937	1917	20190919	22.65	128.15	9.99	18.25	7.79E-05	2.41	1.05E-06	-14.5562	3.28	0.27	0.05002
1938	1917	20190920	23.00	127.10	11.20	23.00	8.14E-05	1.13	-1.00E-06	-9.756	3.49	0.29	0.0541
1939	1918	20190927	13.98	136.55	5.23	13.00	1.99E-05	9.28	8.75E-07	1.893101	1.88	0.15	0.02795
1940	1918	20190928	17.15	129.90	10.24	19.75	9.874E-05	8.13	2.70E-07	5.12907	1.38	0.21	0.03718
1941	1919	20191005	15.10	159.10	8.51	16.50	4.64E-05	10.79	9.44E-07	-3.47439	2.96	0.22	0.03947
1942	1919	20191006	14.63	153.45	15.19	28.25	0.0002514	7.87	3.90E-06	1.5752	2.42	0.26	0.05858
1943	1919	20191007	15.83	147.28	33.57	57.50	0.0014589	7.40	3.64E-06	-8.02965	2.27	0.32	0.12914
1944	1919	20191008	18.08	142.28	36.52	58.75	0.0014082	5.60	3.99E-06	-0.89675	2.82	0.50	0.13882
1945	1919	20191009	20.90	139.93	38.22	60.00	0.0013682	4.05	4.01E-06	2.136031	3.72	0.33	0.13772
1946	1920	20191015	16.33	134.83	7.49	13.50	2.13E-05	4.35	-1.09E-06	-1.08325	1.52	0.20	0.03083
1947	1920	20191016	17.98	131.68	10.49	15.00	2.95E-05	3.51	-6.17E-07	-5.2965	2.07	0.30	0.04031
1948	1920	20191017	19.20	130.00	11.11	18.50	5.90E-05	1.60	3.08E-06	-0.94836	2.09	0.35	0.04794
1949	1920	20191018	20.25	129.30	12.95	22.75	0.0001101	2.90	3.98E-06	5.14186	2.46	0.21	0.04995
1950	1920	20191019	21.68	127.45	17.82	33.75	0.000149	3.25	8.31E-07	-0.23578	3.34	0.24	0.06356
1951	1921	20191018	9.10	158.70	4.55	13.00	1.86E-05	8.80	1.18E-06	-0.07974	1.61	0.14	0.0267
1952	1921	20191019	10.60	154.45	13.49	19.00	0.0001486	6.48	1.23E-06	2.443865	1.55	0.15	0.04069
1953	1921	20191020	12.33	150.25	19.11	30.25	0.0003302	5.33	9.82E-07	8.016196	2.13	0.28	0.06326
1954	1921	20191021	15.08	147.15	26.24	44.50	0.000439	5.63	3.00E-06	17.99347	2.04	0.26	0.07827
1955	1921	20191022	18.65	144.03	28.64	55.25	0.0004723	6.61	-9.85E-07	17.19474	2.96	0.29	0.08917
1956	1921	20191023	22.15	142.20	28.52	49.00	0.0004532	5.95	1.97E-06	17.04321	4.69	0.15	0.08989
1957	1923	20191102	13.08	158.00	10.83	15.75	0.0001368	6.83	1.65E-06	-10.126	2.67	0.28	0.0468
1958	1923	20191103	15.68	154.45	15.97	22.75	0.0002369	3.56	3.22E-06	-4.24451	2.51	0.20	0.05577
1959	1923	20191104	17.98	152.93	22.36	39.00	0.000368	3.40	1.62E-06	-9.28245	2.76	0.21	0.07417
1960	1923	20191105	19.75	151.10	30.11	60.00	0.0003082	2.92	4.16E-06	10.34552	3.46	0.39	0.09778
1961	1923	20191106	21.45	150.65	33.61	59.25	0.0003588	2.79	2.82E-06	14.19296	4.44	0.23	0.09901
1962	1925	20191112	16.45	158.40	9.76	19.00	6.75E-05	7.95	1.49E-06	-6.68122	2.95	0.27	0.04564
1963	1925	20191113	16.93	153.93	14.07	24.50	0.0001729	7.67	3.25E-06	-2.30001	2.51	0.36	0.05675
1964	1925	20191114	17.50	147.50	15.34	29.50	0.000194	7.96	-1.35E-06	24.11866	2.68	0.17	0.05109
1965	1925	20191115	20.68	142.78	22.37	48.00	0.0002607	6.63	4.48E-07	-1.50447	4.40	0.31	0.08351
1966	1925	20191118	22.30	157.70	10.71	13.00	2.44E-05	6.73	1.29E-06	-7.95442	3.39	0.25	0.04305
1967	1926	20191111	12.73	133.50	6.27	13.00	3.81E-05	3.79	1.16E-06	-5.98519	1.95	0.23	0.03522
1968	1926	20191112	12.00	131.03	6.76	13.50	4.81E-05	4.23	-2.16E-09	1.568397	1.55	0.41	0.03902
1969	1926	20191113	13.15	128.15	6.25	15.00	3.72E-05	4.00	9.80E-07	0.109676	1.18	0.17	0.0303
1970	1927	20191118	12.08	137.58	7.83	13.00	3.77E-05	9.35	3.68E-07	8.961369	1.87	0.32	0.03491
1971	1927	20191119	14.20	130.58	8.83	14.00	6.04E-05	7.83	8.68E-07	2.978999	1.89	0.22	0.03488
1972	1927	20191120	17.15	127.50	11.45	18.00	0.0001013	7.74	2.70E-06	8.283233	1.43	0.21	0.0387
1973	1928	20191125	9.25	152.30	5.38	14.00	3.74E-05	9.18	3.55E-07	-2.13935	0.75	0.13	0.02374
1974	1928	20191126	11.15	146.60	10.91	18.50	0.0001323	9.95	1.68E-06	11.81771	0.90	0.29	0.03826
1975	1928	20191127	11.63	140.70	16.33	26.00	0.0003893	4.15	2.49E-06	-3.89138	1.84	0.32	0.06348
1976	1928	20191128	13.13	138.38	16.30	30.00	0.000304	3.51	3.03E-06	-4.49353	1.87	0.25	0.06122
1977	1928	20191129	14.35	137.23	16.74	30.00	0.0002978	2.96	5.78E-06	5.327758	1.89	0.47	0.06958
1978	1928	20191130	13.58	134.28	17.02	31.50	0.0003401	6.32	3.19E-06	18.58053	1.97	0.43	0.06728
1979	1928	20191201	13.15	129.48	19.92	37.00	0.0004584	5.68	2.98E-06	14.41099	1.35	0.38	0.07236
1980	1929	20191220	5.00	146.17	6.58	13.00	3.94E-05	6.33	2.42E-07	5.926695	0.67	0.42	0.03367
1981	1929	20191221	5.78	141.85	8.78	14.00	0.0001456	6.81	5.58E-06	9.623398	1.28	0.41	0.04412
1982	1929	20191222	8.18	137.23	11.42	18.50	0.0001813	7.49	2.28E-06	5.765345	1.24	0.22	0.04119
1983	1929	20191223	10.20	131.58	13.52	22.75	0.000223	7.38	1.77E-06	2.933963	1.59	0.35	0.05186
1984	1929	20191224	10.80	128.00	22.07	30.00	0.000584	8.10	2.79E-06	-0.1086	1.36	0.31	0.0716
1985	2001	20200509	6.88	132.33	6.15	13.50	5.45E-05	2.50	5.10E-06	-5.66712	1.18	0.45	0.04266
1986	2001	20200510	7.90	130.58	6.56	15.00	5.62E-05	2.25	6.08E-06	-3.82859	1.12	0.33	0.04008
1987	2001	20200511	8.93	129.30	6.12	15.00	4.64E-05	2.61	1.82E-06	-4.57354	1.09	0.15	0.03113
1988	2001	20200512	10.95	129.48	11.25	17.00	0.0001031	2.69	3.79E-06	-3.15411	1.39	0.24	0.04229
1989	2001	20200513	12.10	128.03	21.56	33.75	1.77E-04	3.46	1.45E-06	-0.60481	1.37	0.37	0.06272
1990	2009	20200828	16.80	130.18	9.19	16.50	0.0001014	2.32	3.81E-06	-12.8787	1.52	0.31	0.04458
1991	2009	20200829	16.70	129.03	16.68	27.75	0.0002679	1.52	3.65E-06	-6.07615	1.04	0.29	0.05785
1992	2009	20200830	19.10	128.95	23.76	40.00	0.0004741	6.58	2.01E-06	9.529087	1.61	0.29	0.07441
1993	2009	20200831	22.50	128.50	26.63	42.00	0.0006126	8.33	2.29E-06	10.65487	3.20	0.52	0.09568
1994	2010	20200831	22.70	145.65	9.01	15.00	4.82E-05	3.98	-7.28E-08	-12.0553	4.68	0.33	0.05301
1995	2010	20200901	20.75	144.30	12.79	17.75	0.000131	4.49	1.81E-06	-13.9246	4.36	0.28	0.05775
1996	2010	20200902	19.53	140.88	20.13	29.50	0.0003991	5.04	4.37E-06	-0.01881	3.70	0.34	0.07711
1997	2010	20200903	20.78	137.05	28.48	40.75	0.0008229	4.42	7.85E-06	-5.92144	3.29	0.43	0.10683
1998	2010	20200904	22.20	134.30	37.81	56.67	0.0013475	4.60	5.70E-06	-5.52857	3.94	0.68	0.1488
1999	2013	20200926	18.98	159.40	5.99	14.00	2.49E-05	4.18	3.18E-06	-7.72765	2.85	0.24	0.04061

2000	2013	20200927	21.45	158.15	10.99	18.00	6.05E-05	7.72	1.18E-06	4.126092	3.22	0.34	0.04823
2001	2014	20201003	21.60	140.30	4.73	13.00	8.62E-06	1.53	-6.56E-07	-7.86699	4.16	0.23	0.04282
2002	2014	20201004	21.85	139.70	6.82	14.50	1.80E-05	0.99	-2.20E-07	-7.64314	4.22	0.16	0.04377
2003	2014	20201005	22.53	139.23	11.12	18.00	8.494E-05	2.41	9.98E-08	-9.63267	4.42	0.22	0.05294
2004	2018	20201023	10.30	133.48	4.67	13.50	-1.04E-06	5.90	1.62E-06	9.795594	1.69	0.20	0.02927
2005	2018	20201024	13.13	129.50	11.08	16.50	1.34E-04	6.28	2.36E-06	2.218696	1.37	0.16	0.03761
2006	2019	20201026	13.90	141.85	6.74	13.00	2.82E-05	5.15	7.17E-07	0.648518	1.85	0.19	0.03207
2007	2019	20201027	16.05	141.30	9.69	14.50	2.05E-05	2.83	-4.61E-07	4.235768	2.19	0.17	0.03533
2008	2019	20201028	16.63	138.98	11.69	17.75	8.55E-05	3.96	-1.16E-06	4.873425	2.49	0.15	0.0403
2009	2019	20201029	16.60	135.23	20.18	34.75	0.0002584	5.23	2.51E-06	14.66743	1.44	0.22	0.0594
2010	2019	20201030	16.13	131.30	31.15	62.50	0.0003699	4.95	4.51E-07	11.2439	2.44	0.28	0.09072
2011	2019	20201031	15.00	128.30	34.00	68.00	0.0004206	6.72	1.46E-06	6.909391	1.12	0.32	0.09306
2012	2020	20201029	7.28	148.53	9.80	14.00	0.0001428	8.45	1.12E-06	11.49054	0.49	0.13	0.02873
2013	2020	20201030	11.30	142.80	4.94	15.00	2.83E-05	8.61	6.96E-07	7.901698	1.66	0.18	0.02909
2014	2020	20201031	14.13	137.90	6.55	15.75	3.37E-05	7.08	1.90E-06	14.34158	1.96	0.13	0.03129
2015	2020	20201101	16.53	132.28	7.79	18.00	3.86E-05	7.69	-2.34E-06	7.094759	2.48	0.26	0.03779
2016	2020	20201102	19.20	127.95	10.11	18.50	5.146E-05	3.70	1.36E-07	-9.31848	1.77	0.15	0.03732
2017	2020	20201103	19.78	128.28	10.96	20.00	7.44E-05	2.05	1.04E-06	4.989724	2.29	0.26	0.04551
2018	2020	20201104	20.13	128.75	14.58	24.75	0.0001815	2.87	3.17E-06	11.62297	2.45	0.27	0.05549
2019	2022	20201108	9.33	133.63	6.56	14.00	5.43E-05	6.06	2.11E-06	-9.50471	1.53	0.14	0.03157
2020	2022	20201109	12.13	130.60	8.91	17.25	7.678E-05	4.43	2.18E-06	-4.66255	1.52	0.24	0.03911
2021	2022	20201110	13.55	128.35	11.79	21.50	0.0001632	4.34	3.51E-06	3.507509	1.11	0.32	0.04719
2022	2101	20210216	6.70	135.80	8.91	13.00	1.03E-04	5.42	6.84E-07	-0.37486	1.67	0.41	0.04223
2023	2101	20210217	6.60	133.38	8.84	14.00	0.000116	2.35	4.52E-06	3.132275	1.29	0.44	0.04589
2024	2101	20210218	7.15	132.00	12.85	19.00	0.0002988	1.96	9.45E-06	2.809483	1.17	0.74	0.06954
2025	2101	20210219	6.60	130.75	14.17	21.50	3.59E-04	3.02	3.33E-06	1.695558	1.11	0.46	0.06048
2026	2101	20210220	7.33	130.70	11.50	19.00	0.0002447	3.83	3.91E-06	0.351016	1.10	0.66	0.06011
2027	2101	20210221	9.37	128.53	9.12	17.00	7.83E-05	8.25	4.13E-06	9.03846	1.16	0.36	0.04018
2028	2102	20210412	7.80	138.00	7.33	13.00	2.40E-05	1.02	3.66E-06	-16.0869	1.41	0.40	0.04192
2029	2102	20210413	7.88	137.50	7.34	15.25	7.068E-05	1.11	5.00E-06	-19.7015	1.34	0.56	0.05063
2030	2102	20210414	8.43	136.80	14.35	21.50	0.0003038	1.22	5.12E-06	-17.6575	1.16	0.48	0.06283
2031	2102	20210415	8.70	135.83	18.83	26.50	0.0005983	2.30	5.53E-06	-1.63799	1.21	0.49	0.07764
2032	2102	20210416	9.35	133.40	24.01	40.75	0.0009748	4.96	4.59E-06	2.981383	1.52	0.41	0.09826
2033	2102	20210417	11.65	129.70	33.11	64.50	0.0014683	5.28	5.24E-06	-7.34882	1.44	0.60	0.14071
2034	2102	20210418	13.25	127.40	39.43	70.00	0.0018411	3.38	7.72E-06	-25.2853	1.26	0.64	0.16402
2035	2102	20210422	21.50	127.40	25.23	40.00	0.0004712	7.01	2.36E-06	-7.16142	3.31	0.44	0.08868
2036	2102	20210423	22.50	128.40	24.45	33.00	0.0005232	16.25	6.44E-07	-13.8752	3.22	0.40	0.07989
2037	2102	20210424	22.23	132.87	15.10	19.33	0.0001871	7.03	3.40E-06	-13.4046	3.28	0.30	0.05766
2038	2102	20210425	22.15	136.90	12.89	18.00	1.17E-04	10.61	-2.64E-06	-22.2716	3.60	0.23	0.04773
2039	2103	20210529	6.13	135.35	6.49	13.00	6.906E-05	3.85	5.55E-06	-3.41214	1.58	0.47	0.0449
2040	2103	20210530	6.53	132.35	12.27	15.75	0.0001685	4.35	7.38E-06	0.436584	1.14	0.47	0.05213
2041	2103	20210531	9.05	129.53	13.38	18.50	0.0002241	5.33	5.08E-06	-6.1339	1.20	0.60	0.05924
2042	2103	20210601	10.10	127.30	13.94	20.00	0.0001363	4.82	2.43E-06	6.032969	1.23	0.30	0.04558
2043	2105	20210620	10.53	149.78	7.36	13.00	1.92E-05	4.51	8.47E-07	5.691755	0.94	0.27	0.03098
2044	2105	20210621	12.08	146.18	11.07	13.00	6.25E-05	5.96	1.03E-06	6.206225	1.39	0.28	0.0361
2045	2105	20210622	14.45	142.40	11.53	14.50	5.35E-05	4.79	2.62E-06	5.529626	1.88	0.25	0.03955
2046	2105	20210623	17.28	140.65	12.83	21.00	8.596E-05	3.48	3.84E-07	9.636909	2.83	0.23	0.04764
2047	2105	20210624	19.40	139.78	14.52	24.25	0.0001033	3.00	1.51E-06	8.92481	3.38	0.24	0.05466
2048	2105	20210625	21.87	139.13	17.52	31.00	0.0001659	4.79	6.52E-06	3.016352	4.09	0.47	0.07557
2049	2106	20210716	19.30	134.45	7.24	13.00	3.84E-05	5.38	6.78E-07	-11.5756	2.85	0.27	0.04056
2050	2106	20210717	21.35	132.98	7.70	15.75	2.89E-05	2.89	2.57E-06	-6.19536	2.78	0.31	0.04482
2051	2106	20210718	22.65	132.35	10.50	19.00	6.39E-05	1.61	3.53E-06	-0.76588	3.41	0.35	0.05407
2052	2112	20210818	15.90	136.90	9.10	13.00	2.50E-05	8.48	1.35E-06	-1.44751	1.89	0.29	0.03586
2053	2112	20210819	17.63	133.63	9.07	13.50	3.80E-05	5.45	2.92E-06	0.80482	1.85	0.40	0.04228
2054	2112	20210820	19.00	130.35	10.37	16.50	5.53E-05	5.50	1.69E-06	1.7098	2.20	0.15	0.03773
2055	2112	20210821	21.97	127.67	14.31	21.00	0.000126	4.63	-6.57E-07	2.40278	3.38	0.32	0.0547
2056	2114	20210905	13.05	139.75	7.03	13.00	2.52E-05	2.94	1.50E-06	8.465581	1.98	0.15	0.03247
2057	2114	20210906	14.30	138.25	7.95	17.75	3.95E-05	3.95	8.71E-07	20.924	2.00	0.15	0.03456
2058	2114	20210907	16.08	135.18	15.69	39.25	0.000164	5.66	4.03E-08	-1.54738	1.50	0.18	0.05462
2059	2114	20210908	15.68	130.75	21.48	61.00	0.0002453	5.48	3.15E-06	-19.3945	2.06	0.30	0.08255
2060	2114	20210909	15.65	127.50	26.17	60.00	0.0001101	5.20	1.81E-06	-2.75404	1.11	0.35	0.07577
2061	2116	20210922	11.60	148.00	6.88	14.00	4.39E-05	7.05	4.81E-08	-1.30009	1.23	0.17	0.02906
2062	2116	20210923	13.45	144.05	10.05	16.50	0.000103	6.90	1.66E-06	-3.72433	1.77	0.28	0.0412
2063	2116	20210924	15.53	139.88	15.80	25.25	0.0002107	4.93	1.97E-06	-17.4242	2.01	0.21	0.05413
2064	2116	20210925	17.83	137.55	24.77	43.75	0.0003873	2.53	8.93E-07	-13.0819	2.46	0.35	0.08228
2065	2116	20210926	18.95	136.73	32.99	59.50	0.0007307	1.31	8.71E-06	-8.36318	2.59	0.70	0.1244
2066	2116	20210927	20.05	136.50	27.68	49.00	0.0008137	1.91	9.36E-06	-7.946	3.20	0.72	0.12157
2067	2116	20210928	21.80	135.73	26.55	50.00	0.000966	3.47	7.32E-06	6.829576	4.04	0.73	0.12794
2068	2118	20211008	14.40	132.73	4.14	15.00	5.99E-06	4.02	9.43E-07	-20.1152	2.07	0.10	0.03108
2069	2118	20211009	16.13	130.25	7.64	18.00	3.49E-05	3.37	-1.17E-06	-14.2153	1.65	0.33	0.04001
2070	2118	20211010	17.30	128.45	7.71	20.00	3.20E-05	5.39	5.52E-06	-13.7445	1.17	0.34	0.04233
2071	2119	20211010	17.33	158.97	12.55	20.33	0.0001122	3.93	3.20E-06	10.89235	2.82	0.23	0.04936
2072	2119	20211011	18.73	155.65	13.06	23.00	1.20E-04	6.07	4.56E-06	14.81393	2.53	0.28	0.05137
2073	2119	20211012	20.08	151.93	10.77	20.50	8.02E-05	4.35	4.14E-06	14.23853	3.75	0.32	0.05442
2074	2119	20211013	22.60	152.77	10.54	15.00	5.21E-05	5.45	-1.38E-06	21.13857	4.49	0.17	0.04423
2075	2120	20211023	11.23	141.08	3.82	13.00	1.30E-05	4.46	1.29E-06	-19.2084	1.78	0.09	0.02831
2076	2120	20211024	13.53	139.25	5.02	15.00	1.19E-05	5.43	4.04E-07	-22.1539	2.01	0.13	0.0319
2077	2120	20211025	17.75	138.35	9.05	18.50	7.467E-05	3.80	1.63E-06	-7.58639	2.62	0.19	0.04316
2078	2120	20211026	19.73	138.80	15.89	24.50	0.0002443	2.84	2.98E-06	-4.61539	2.93	0.21	0.05936
2079	2120	20211027	21.60	139.68	21.94	31.00	0.0003934	3.27	2.71E-06	0.102523	4.07	0.32	0.07944
2080	2121	20211129	11.98	141.70	8.08	14.75	2.31E-05	5.31	9.35E-07	-11.0844	1.71	0.25	0.03615
2081	2121	20211130	13.05	137.95	12.71	22.25	0.0001577	3.92	2.01E-06	-12.6297	1.84	0.21	0.04814
2082	2121	20211201	14.83	135.93	20.45	29.75	0.0004712	3.64	4.45E-06	13.46417	1.78	0.33	0.07098
2083	2121	20211202	17.90	136.45	25.37	44.25	0.0006921	6.70	1.54E-06	-22.9424	1.96	0.31	0.08931
2084	2121	20211203	21.65	139.50	28.76	55.00	0.0005541	12.26	3.29E-06	-19.8698	4.06	0.40	0.10297
2085	2122	20211212	5.10	142.80	7.32	15.00	0.0001435	4.36	4.08E-06	2.465853	0.95	0.39	0.04225
2086	2122	20211213	5.78	140.18	9.54	19.00	0.0001907	6.27	7.00E-06	8.877125	1.76	0.40	0.05188
2087	2122	20211214	7.83	135.38	16.10	28.25	0.0003706	6.49	5.86E-06	5.87737	1.31	0.42	0.06526
2088	2122	20211215	9.03	130.68	20.03	42.50	0.0005089	5.97	4.42E-06	6.822159	1.41	0.53	0.08368
2089	2122	20211216	9.60	127.70	30.39	62.00	0.0007243	6.69	7.69E-06	15.66564	1.22	0.45	0.10556
